# Smart Face Masks as Wearable Respiratory Sensors: A Review of Sensor Technologies, Materials, and Future Directions

**DOI:** 10.1002/adhm.202504991

**Published:** 2026-04-06

**Authors:** Negin Faramarzi, Naseeh Backer Kannanthodi, Alice Nicole Casling, Babar Ali, Samira Lakouraj Mansouri, Alessandro Giuseppe D'aloia, Hossein Cheraghi Bidsorkhi, Maria Sabrina Sarto

**Affiliations:** ^1^ Department of Electrical and Energy Engineering (DIEE) and Energy Engineering (DIAEE) Sapienza University of Rome via Eudossiana 18 Rome Italy

**Keywords:** health monitoring, innovations, nanomaterials, respiration monitoring, smart mask, wearable sensors

## Abstract

Smart face masks are rapidly evolving into versatile wearable devices capable of simultaneously tracking a variety of physical and biochemical signals for both personal healthcare and environmental monitoring. This review focuses on respiration monitoring and highlights recent advances in materials, device architectures, and sensor design for mask‐integrated platforms. We categorize current approaches into six sensor types, humidity, gas, temperature, pressure, strain, and triboelectric, and summarize their transduction principles, material/fabrication strategies, and representative demonstrations for quantifying respiratory metrics. Finally, we discuss key challenges for real‐world use, including sensor cross‐sensitivity, long‐term stability, and user safety, noting that everyday activities like talking or head movement can affect measurements. We also highlight future opportunities in cost‐effective manufacturing, modular designs, and AI‐enabled data analysis to improve accuracy and reliability in respiratory monitoring.

## Graphical abstract

This review highlights recent advances in smart face masks that actively monitor breathing. By integrating humidity, gas, temperature, pressure, strain, and triboelectric sensors, these masks track key respiratory parameters in real time. The article summarizes sensor mechanisms, compares performance across studies, and discusses challenges and future directions for improving respiratory health monitoring and user adaptability.

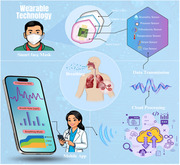

## 1 Introduction

Respiration is one of the most critical physiological processes, serving as a vital indicator of individual health and well‐being. The human respiratory system, essential for oxygen exchange, is highly vulnerable to airborne pathogens such as viruses and bacteria, which can compromise its function and lead to serious health risks [1, 2, 3, 4, 5, 6]. Face masks have emerged as essential protective barriers, covering the nose and mouth to prevent the inhalation of harmful pathogens [7, 8]. By shielding the respiratory system, face masks not only safeguard individual health but also reduce the transmission of infectious diseases. Although masks have been used for centuries to control the spread of airborne illnesses, their widespread adoption became particularly prominent with the emergence of the COVID‐19 pandemic, underscoring their critical role in public health protection [7, 8, 9, 10, 11, 12].

Recent innovations have led to the development of “smart face masks,” which integrate wearable sensor technologies to enhance respiratory health monitoring [9, 13, 14]. Wearable sensor technologies have already revolutionized health monitoring by enabling the detection of a wide range of physiological signals, from biophysical indicators like motion [15, 16, 17, 18, 19, 20], heart rate [21, 22], and respiration [23, 24, 25, 26, 27, 28, 29] to biochemical markers such as glucose [30, 31, 32, 33, 34] and lactate [35, 36, 37] levels. In the specific domain of respiration monitoring, wearable technologies play a transformative role by detecting key parameters such as breath rate, depth, and patterns in real time [38, 39, 40, 41]. This capability is particularly critical for identifying irregularities in breathing, which can serve as early warning signs of respiratory or systemic health issues [42]. Unlike conventional masks, smart face masks represent a significant leap forward in safeguarding respiratory health. By integrating advanced sensing mechanisms, they actively monitor respiratory parameters in real time, enabling continuous assessment of the respiratory system's condition and enhancing individual well‐being.

These masks represent a breakthrough in wearable health technology by transforming exhaled breath into a continuous, noninvasive data stream, eliminating the need for bulky equipment. Their true innovation lies with their dual‐sensing capability, offering comprehensive health and environmental monitoring. On the inside, they can track physical signals such as breathing patterns, cough frequency, body temperature, and even heart rate, while simultaneously analyzing exhaled biomarkers (e.g., viruses or chemical compounds) for early disease detection. On the outside, they act as environmental sentinels, detecting pollutants, harmful gases, and allergens, and providing real‐time alerts about air quality. This unique combination of personal health monitoring and environmental protection offers a holistic approach to safety, unlike traditional systems that rely on multiple devices or complicated setups. Smart face masks are lightweight, portable, and multifunctional, designed for practical daily use. By empowering individuals to take charge of their health while contributing to a safer, healthier environment, these masks blend advanced technology with everyday convenience, paving the way for a smarter, more connected approach to well‐being [14, 43, 44, 45, 46, 47, 48, 49].

The operation of these functionalized masks relies on three key sensing mechanisms. (1) Physical signal sensing, this category involves monitoring physical parameters related to respiration, such as breathing rate, intensity, and patterns. Sensors like humidity, pressure/strain, temperature, and triboelectric sensors fall under this category. They provide real‐time data on respiratory activity, enabling the detection of abnormalities such as sleep apnea or asthma. (2) Exhalation biochemical sensing, here, the focus shifts to detecting biochemical markers in exhaled breath, such as CO_2_, acetone, or NH_3_. Gas sensors are commonly used in this category. While still related to respiration, these sensors go beyond physical monitoring to analyze biomarkers, offering insights into metabolic or respiratory health for disease diagnosis and management. (3) Inhalation environmental sensing, this category is centered on monitoring inhaled pollutants, such as volatile organic compounds (VOCs) or particulate matter. While its primary goal is environmental evaluation, it also provides valuable context for respiratory health by assessing air quality and potential exposure to harmful substances.

Since smart face masks capture diverse signals, including physical, biological, and environmental data, the raw data (e.g., electrochemical voltage, resistance, current) is first conditioned and preprocessed on the device's integrated chip. This involves filtering out noise, enhancing weak signals, and organizing the data for transmission [51, 52]. Preprocessing is essential due to the limited power and computing capabilities of smart face masks. Once the data is ready, it is sent wirelessly via technologies such as Bluetooth, Wi‐Fi, or NFC to a smartphone or cloud platform [53, 54, 55]. Following transmission, the data is further analyzed, typically in the cloud or at the edge, using advanced techniques such as machine learning and big data analytics. These methods help identify and quantify specific markers within the signals, transforming raw data into clear, numerical information. This refined data is then used to provide real‐time insights to the wearer regarding their health status and other physiological conditions [56, 57, 58].

To enable high‐performance sensing, smart masks leverage advanced functional materials, such as nanomaterials, that enhance sensitivity and selectivity. These innovations allow for the precise detection of weak physical signals and trace biochemical markers. A detailed overview of the advanced materials utilized in the design of smart mask sensors is discussed in the following section. The synergy between advanced materials and modern communication systems enables the creation of intelligent, wearable devices poised for commercialization. Such sensors offer enhanced precision, sensitivity, and the ability to detect a diverse spectrum of biomarkers. Coupled with AI‐driven communication technologies, these systems streamline data transmission to centralized platforms, empowering real‐time analysis for health monitoring and early disease diagnosis. By bridging cutting‐edge materials science with robust communication frameworks, this integration transforms theoretical innovations into practical tools for proactive healthcare.

**FIGURE 1:**
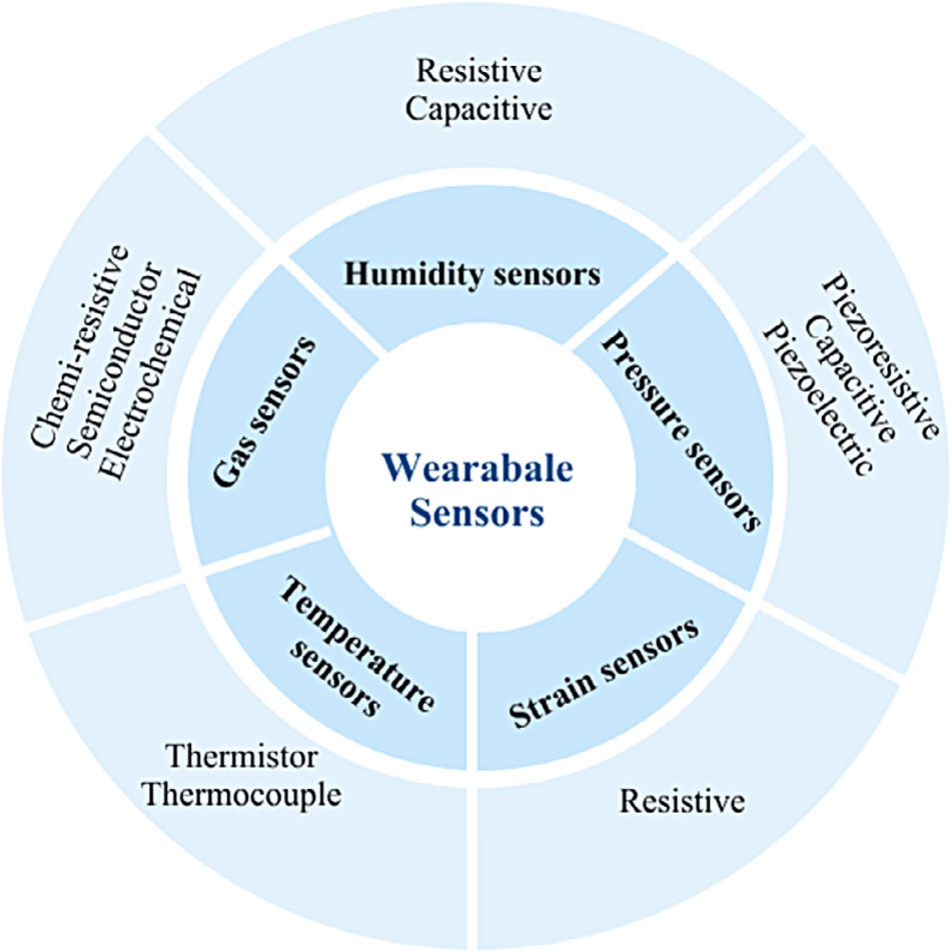
Different types of integrated sensors and their modes of operations, created by the authors using Figma.

The functionalization of face masks has seen explosive growth, with nearly 10,000 publications since 2019 (Science Citation Index), reflecting the rapid advancements in this field. Among these innovations, the first smart face mask is developed by Nahal et al. in 2016 to overcome the limitations of traditional preoperative pulmonary function testing for lung cancer patients [59]. This pioneering design featured gas sensors embedded in a rubber mask to measure oxygen and carbon dioxide levels in exhaled breath. The data was wirelessly transmitted to a smartphone, enabling real‐time monitoring through a dedicated mobile application. This breakthrough laid the foundation for modern smart face masks, which now integrate advanced sensing technologies to monitor respiratory health more effectively (Figure 1).

Since then, the field has rapidly evolved from simple filtration toward multifunctional, intelligent masks. For example, copper‐nanowire transparent filters enable reusable masks by combining mechanical filtration, antimicrobial activity, and Joule‐heating self‐sterilization for rapid pathogen decontamination [60]. At a larger scale, biomimetic air purification systems inspired by human respiratory processes have been developed to continuously remove particulate matter and regulate indoor CO_2_ via microbubble‐based gas exchange [61]. Meanwhile, machine‐learning‐assisted masks optimize stretchable nanofiber filters, dynamically adjusting pore size to balance filtration efficiency and breathability [62]. Other studies focus on wearable respiratory monitoring, integrating a laser‐induced graphene humidity sensor with a dielectric elastomeric pressure array to track breath patterns and mask fit, highlighting the potential of multifunctional materials for real‐time health monitoring [63].

Several review papers have explored various aspects of smart face masks, including functional classifications [64, 65, 66], manufacturing methods [67], security considerations [68, 69], and multimodal and multiplexed sensing for applications in health management and environmental monitoring [13]. Recent comprehensive reviews, such as those by Liang et al. [70], have provided excellent overviews of the field. However, these recent works predominantly emphasize complex biosensing mechanisms, such as the use of biological probes to detect specific analytes or treat physical respiration monitoring as a secondary application within generalized wearable systems instead of primary objective. This presents a significant gap in the literature regarding the specific engineering of purely physical and chemical sensors for continuous respiratory telemetry.

Addressing this gap, our review distinctly differs from prior articles by making our primary focus the material innovation driving physical and chemical respiratory sensors. We establish this through a detailed explanation of material choices, beginning by categorizing functional nanomaterials based on their dimensionality (0D to 3D). We subsequently detail the fundamental operating principles of six specific sensor types: humidity, pressure, strain, gas, temperature, and self‐powered sensors. Within each of these categories, we elaborate extensively on the material innovations that enhance sensitivity, selectivity, and response times for breath analysis, while critically evaluating the specific advantages and inherent limitations of each sensing mechanism. Finally, beyond isolated material innovations, we critically evaluate how these sensors are practically embedded into mask architectures via hybrid or monolithic integration, assess the energy efficiency of communication protocols like Bluetooth Low Energy (BLE), and discuss the role of AI‐driven data processing.

Finally, we present a new perspective on the synergistic integration of multiple sensing modalities, highlighting how combined, multisensor systems can deliver a holistic, multidimensional view of respiratory health. Together, these elements position this review as a distinctive and timely resource for researchers, clinicians, and engineers aiming to advance smart mask technology beyond current capabilities.

## 2 Functional Material

Wearable sensors, including smart face masks, consist of various components, including substrates that support sensing materials and electronic components, and sensing materials that detect target signals [71, 72]. Choosing the right materials for each part is essential for optimizing sensor performance research has examined various sensitive materials, including carbon‐based materials (e.g., graphene, GO, carbon nanotubes (CNTs), reduced graphene oxide (rGO), and graphitic carbon nitride), polymers, oxide‐based, novel 2D materials like Ti_3_C_2_T*_x_*, MoS_2_, and WS_2_, and composite materials. In this section, we explore the most commonly used sensitive and substrate materials for developing smart mask sensors.

### 2.1 Flexible Substrate

The flexible substrate serves as the primary base of a sensor, providing a skeleton onto which functional sensing elements are integrated. These substrates must exhibit inherent mechanical compliance, including stretchability and elasticity, to achieve conformal adaptability in sensor design. Beyond mechanical robustness, substrates intended for wearable and mask‐based sensors must also demonstrate excellent biocompatibility and biodegradability [71, 72, 73, 74]. Biocompatibility is especially important for interfaces that contact the skin and respiratory airflow, reducing the risk of irritation, inflammation, or other adverse biological responses during prolonged use, while biodegradable materials help mitigate environmental impact, particularly for disposable health devices. For example, biodegradable smart face‐mask systems that employ air‐permeable sensing elements have been proposed to improve eco‐friendliness in continuous respiratory monitoring applications.

In addition to these properties, breathability is a critical design parameter for smart mask substrates, as the material directly interfaces with inhaled and exhaled airflow. Breathability, often quantified by air and water‐vapor transmission rates, enables efficient gas exchange, preventing the substrate from impeding normal respiration, reducing heat and moisture buildup, and maintaining wearer comfort during extended periods of use. Many wearable sensor designs deliberately employ porous, fibrous, or microstructured architectures (e.g., electrospun nanofiber membranes or perforated textile matrices) to enhance permeability without substantially sacrificing mechanical strength or signal fidelity. Such structures not only facilitate airflow but also improve moisture management by allowing water vapor to escape, helping avoid condensation that could otherwise impair sensing performance and user comfort. The literature on flexible, breathable electronics highlights the importance of engineered porosity and interlaced fiber networks in balancing breathability with mechanical and electrical functionality for health monitoring [75, 76]. Together, biocompatibility, biodegradability, and breathability ensure that wearable mask sensors can be worn safely and comfortably over extended periods, integrate seamlessly with the user's respiratory dynamics, and address sustainability concerns in disposable or single‐use formats.

Commonly employed substrate materials for wearable and mask‐integrated sensors include textile‐based platforms [77, 78, 79, 80, 81], (e.g., conductive fabrics, polyester, and cotton), paper‐derived materials [82, 83, 84, 85], and elastomeric polymers such as polydimethylsiloxane (PDMS) [86, 87, 88, 89], Ecoflex [90, 91], and thermoplastic polyurethane (TPU) [92, 93]. Textile and paper‐based substrates are particularly attractive for smart face mask applications due to their intrinsic porosity and interconnected fiber networks, which naturally facilitate air and moisture permeability while maintaining mechanical flexibility. In contrast, elastomeric polymers such as PDMS and Ecoflex offer excellent stretchability, conformability, and biocompatibility; however, in their bulk, nonporous forms, they exhibit limited breathability. To address this limitation, recent strategies involve engineering micro‐/nanoporous architectures, perforated structures, or electrospun fibrous configurations to enhance gas exchange and vapor transmission while preserving mechanical integrity and sensing stability. Through such structural optimization, these materials can be adapted to meet the multifunctional requirements of smart face mask substrates, including flexibility, biological safety, environmental sustainability, and airflow compatibility.

### 2.2 Sensing Material

**FIGURE 2:**
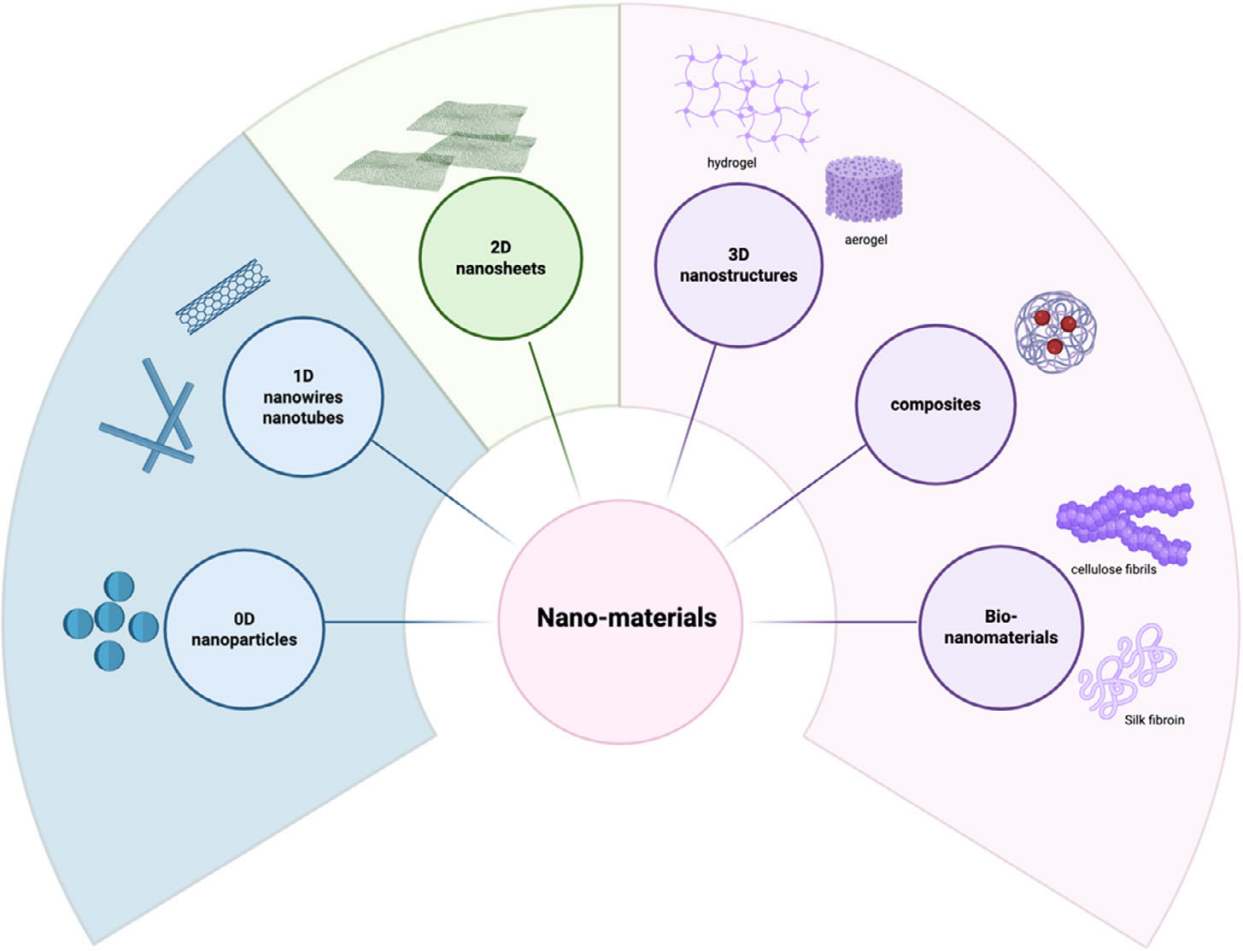
Materials used in smart mask production, created in BioRender. Faramarzi, N. (2025) https://BioRender.com/8sugmpv.

The sensing element of a wearable sensor plays a critical role in transforming specific physical, chemical, or environmental signals into measurable electrical outputs, necessitating materials with high electrical conductivity to ensure optimal functionality. To achieve high sensitivity, long‐term stability, and reliable performance in wearable sensors, a wide array of nanomaterials has been extensively explored. These nanomaterials are typically classified based on their dimensional structure, including 0D, 1D, 2D, and 3D architectures, which are illustrated in Figure 2. Each category offers unique advantages, such as exceptional electrical and mechanical properties, high surface‐to‐volume ratios, and the ability to be functionalized for specific applications. These attributes make nanomaterials highly versatile and indispensable for advancing the performance and applicability of wearable sensor technologies.

#### 2.2.1 0D Nanomaterials

Metal nanoparticles, such as gold (Au), silver (Ag), are widely used as 0D nanomaterials in smart mask sensors due to their high conductivity and large surface area. These nanoparticles are integrated into substrates or electrodes to enhance sensitivity by facilitating efficient electron transfer. Various fabrication methods are employed to synthesize these materials, including chemical [94], physical, and biological processes [95].

#### 2.2.2 1D Nanomaterials

##### 2.2.2.1 Carbon Nanotube (CNT)

CNTs, with their 1D tubular nanostructure, are prominent 1D nanomaterials for smart face mask sensors due to their exceptional electrical conductivity, tunable alignment, mechanical flexibility, high aspect ratio, and low density. These properties enable their integration into diverse architectures, such as thin films [96], aerogels [97], sponges [98], and vertically aligned forests [99], through tailored synthesis methods like chemical vapor deposition (CVD) [100], or solution processing [101]. Such structural versatility allows CNTs to serve as highly sensitive, spatially resolved platforms for monitoring multiple physiological and environmental parameters. For instance, their conductivity changes in response to temperature fluctuations, humidity gradients, or mechanical strain/pressure, while their surface functionalization can detect biological analytes (e.g., biomarkers in breath).

##### 2.2.2.2 Metal Nanowire

Metal nanowires (NWs), such as gold, silver, and copper, are widely used in wearable sensors as percolated networks or aligned structures to act as active sensing components. There are two primary sensing mechanisms, namely, charge transfer and percolation‐based electron transport. In gas/humidity sensors [102], adsorption of molecules on the nanowire surface leads to charge transfer, altering the electrical conductivity. In pressure/strain sensors, mechanical deformation disrupts the nanowire network, resulting in resistance changes. These nanowires exhibit high sensitivity due to their high conductivity and large surface‐to‐volume ratio. They are typically synthesized via solution‐phase reduction, where metal precursors are chemically reduced in the presence of stabilizing agents, allowing precise control over their properties for optimized performance.

#### 2.2.3 2D Nanomaterials

##### 2.2.3.1 Graphene and Its Derivatives

Graphene, a 2D monolayer of carbon atoms arranged in a hexagonal lattice, exhibits extraordinary mechanical, electrical properties, ultrahigh specific surface area, a high Young's modulus, and exceptional flexibility. Its unique characteristics, such as atomic‐scale thickness, tunable charge carrier mobility, and adaptable electronic behavior, make it an ideal material for flexible electronics and high‐performance wearable sensors [103, 104]. Graphene can be tuned for specific applications through chemical functionalization (e.g., attaching hydrophilic groups to its hydrophobic surface) or doping (e.g., nitrogen/boron doping). Graphene and its derivatives, such as graphene oxide (GO) [105], rGO [106], and graphene nanoplatelets [107, 108], are widely employed as active sensing material or signal transfer electrode. Common synthesis methods include CVD, mechanical exfoliation, chemical reduction of GO, and epitaxial growth, each offering distinct advantages in scalability, purity, and defect control.

##### 2.2.3.2 2D Materials Beyond Graphene

2D transition metal dichalcogenides (TMDs), such as WS_2_ and MoS_2_, exhibit graphene‐like layered structures with high electrical conductivity and large specific surface areas, enabling efficient electron transport and precise sensing capabilities, along with good biocompatibility. Their general formula MX_2_ (where M = transition metals like W or Mo, and **X** = chalcogens such as Te, Se, or S) endows them with versatile electrical, mechanical, chemical, and optical characteristics. These materials are synthesized via methods like CVD [109], solvent exfoliation [110], laser‐assisted chemical conversion [111, 112], and chemical intercalation [113, 114]. Besides, 2D materials like MXenes have gained significant research attention due to their unique physicochemical properties. MXenes are significant due to their remarkable biocompatibility, extensive interlayer spacing, environmental friendliness, and relative safety, offering a range of substantial attributes. MXenes are synthesized by selectively etching the A‐element layers from their parent ternary layered MAX phases (Mn+1AX*n*), where M represents transition metals (e.g., Ti, Mo, Ta, V, Zr), A represents Group III–IV elements, X is carbon (C) or nitrogen (N), and *n* ranges from 1 to 3. This chemical removal of the A‐layer creates 2D MXene structures, retaining the M–X bonding framework while introducing tunable surface properties for sensing applications [115].

#### 2.2.4 3D Nanomaterials

3D nanomaterials are nanostructured in all three spatial dimensions, exhibiting exceptional surface‐to‐volume ratios, customizable porosity, and unique mechanical/electrochemical properties. These materials, including aerogels [97] and hydrogels [116, 117], are particularly promising for smart face mask sensors. Aerogels, synthesized via sol–gel methods followed by freeze‐drying, form ultralightweight, porous network from carbon, metal oxides, or polymers, ideal for humidity or gas detection. Hydrogels, constructed through crosslinking of hydrophilic polymers (e.g., cellulose, polyacrylamide), create biocompatible 3D networks that respond dynamically to moisture, enabling real‐time breath analysis.

#### 2.2.5 Composite Materials

Hybrid materials are engineered by combining two or more distinct constituents to synthesize a novel material system with physical and chemical properties distinct from those of its individual components. These composites exhibit superior performance enhancements compared to single‐component materials, addressing inherent limitations such as brittleness, susceptibility to corrosion or oxidation, biocompatibility challenges, and functional constraints like the relatively high sheet resistance observed in graphene. Structurally, composite materials consist of a base matrix (a continuous, soft phase) and a reinforcing filler (a dispersed phase characterized by high stiffness, strength, and low thermal expansion). Their classification is typically based on three primary criteria: (1) matrix type (e.g., polymer matrix composites, metal matrix composites, or ceramic matrix composites (CMCs)), (2) reinforcement morphology (particulate, fibrous, or laminar), and (3) scale of constituent integration (e.g., nanocomposites) [118].

Among hybrid materials, core–shell structure, which enable the integration of organic and inorganic phases through synergistic interactions, have emerged as a promising strategy to enhance the reliability, accuracy, and durability of composite systems. The limitations of core may be solved with addition of shell. For example, Kim et al. developed a humidity sensor using a CNT@chitosan–polyamidoamine composite with a core–shell structure. This design achieved high sensitivity, minimal hysteresis, long‐term operational stability, and mechanical resilience under repeated bending, demonstrating the efficacy of core–shell configurations in optimizing sensor performance [119].

Beyond core–shell methodologies, advanced fabrication techniques such as mesoscopic reconstruction offer innovative pathways for composite synthesis. In one instance, a CNT/silk fibroin (SF) sensor was fabricated via seeded nucleation and wet spinning. Here, CNTs functioned as nucleation seeds, directing the self‐assembly of SF into a hierarchical mesostructure. The resulting hybrid network leveraged CNTs as conductive pathways while SF provided a humidity‐responsive framework, exemplifying the potential of tailored material architectures to integrate complementary functionalities [120].

#### 2.2.6 Bio‐Nanomaterials

Many nanomaterials, although they have remarkable properties, often face limitations in clinical applications because of low biocompatibility. This issue has encouraged the use of biomaterials like cellulose and silk fibroin, which are valued for their safety, biodegradability, and compatibility with biological systems. Silk fibroin [121], which comes from silkworms or different types of spiders, is a natural polymer that can be functionalized to increase its usefulness. Cellulose is one of the most studied biopolymers due to its abundance on Earth, obtained from different sources (e.g., plants). It is known for its enhanced functionality through simple surface modification. Cellulose nanomaterials can be grouped into five types, among which cellulose nanocrystals (CNCs) and cellulose nanofibrils (CNFs) [122] are the most commonly used. CNCs are made by acid hydrolysis, where the disordered regions dissolve and only the crystalline parts remain [123], while CNFs are produced by mechanical treatments that tear the cellulose apart through deformation [124]. Both materials address critical gaps in clinical wearables by combining natural safety with advanced functionality (Table 1).

**TABLE 1:** Summary of materials and their functionalities.

Example	Role in smart mask sensor	Classification	Key properties relevant to masks	Typical sensing targets/functions
Cotton, polyester, and conductive fabrics	Flexible substrate	Textile based	Breathable, flexible, good conformability, biocompatible	Mechanical strain, integration platform for sensing layers
Cellulose paper, paper tissue	Flexible substrate	Paper based	Low cost, lightweight, porous, biodegradable	Disposable mask body, gas/humidity pathways
PDMS, Ecoflex, TPU	Flexible substrate	Polymer based	Stretchable, elastic, good skin contact, encapsulation	Substrate for strain/pressure sensors, encapsulation of electrodes
Au, Ag nanoparticles	Sensing layer	0D nanomaterial	High conductivity, large surface area, strong surface chemistry	Gas and biomarker detection via surface interactions, signal amplification
Carbon nanotubes (CNTs)	Sensing layer	1D carbon nanomaterial	High aspect ratio, excellent conductivity, flexible, tunable alignment	Strain/pressure, temperature, humidity, breath biomarkers
Graphene nanoplatelets (GNPs), GO, rGO	Sensing layer	2D carbon nanomaterial	Ultrahigh surface area, excellent conductivity, flexible, functionalizable	Gas, humidity, strain, temperature, multifunctional sensing
2D TMDs (MoS_2_, WS_2_)	Sensing layer	2D semiconductor	Tunable bandgap, good conductivity, high surface area, biocompatible	Gas sensing, strain sensing, optoelectronic responses
1D metal nanowires (Ag, Au, Cu NWs)	Sensing network	1D metal nanomaterial	High conductivity, high surface‐to‐volume ratio, deformable networks	Gas/humidity sensing (charge transfer), strain/pressure (network breakage)
2D MXenes (e.g., Ti_3_C_2_T*_x_*)	Sensing layer, sometimes substrate‐like films	2D carbide/nitride	High conductivity, hydrophilicity, good biocompatibility, large surface area	Gas sensing, humidity, pressure, multimodal sensing
3D aerogels (carbon, oxide, polymer)	Sensing framework	3D porous network	Ultralightweight, huge surface area, tunable porosity	Gas/humidity sensing, pressure sensing via deformation
3D hydrogels	Sensing matrix/substrate	3D polymer network	Biocompatible, moisture‐responsive, flexible	Humidity/breath analysis, skin‐contact sensors
Polymer matrix composites (e.g., CNT/polymer, graphene/polymer)	Sensing film, stretchable conductor	Composite, nanocomposite	Synergistic mechanical + electrical properties, improved durability	Strain, pressure, gas/humidity, multimodal sensing
Bio‐based materials (silk fibroin, cellulose CNF/CNC)	Substrate, sensing matrix, binder	Biopolymer/bio‐nanomaterial	Biocompatible, biodegradable, tunable functionalization	Skin‐contact sensors, breathable substrates, humidity/breath sensing

## 3 Smart Mask System Integration

### 3.1 System Integration

The successful realization of a smart mask extends beyond the development of functional sensor materials to comprehensive system‐level integration. This process requires a careful balance between measurement accuracy, electronic performance, energy efficiency, and ergonomic comfort. Accurate and reliable sensing is fundamentally dependent on optimized sensor placement. The spatial configuration within the mask significantly influences measurement fidelity. For example, temperature readings may vary depending on whether the sensor is positioned near the breathing zone, at the bottom edge, or along the side of the mask, due to differences in airflow, exhaled breath concentration, and thermal gradients. Similar considerations apply to humidity and gas sensors, where proximity to the nose and mouth directly affects response characteristics. Therefore, each sensor type requires strategic placement to ensure representative and reproducible data acquisition under real‐use conditions. From a hardware perspective, wireless smart mask systems can generally be realized through hybrid integration or monolithic integration strategies. In hybrid integration, the sensing element is physically connected to an external circuit board that contains the microcontroller, power unit, and wireless communication module (e.g., NFC or Bluetooth). This approach offers design flexibility and facilitates modular upgrades but may increase structural complexity due to interconnections. In contrast, monolithic integration involves directly embedding the sensing component into the wireless module or antenna structure, enabling simultaneous sensing and signal transmission within a unified architecture. This method simplifies structural design and enhances compactness, which is particularly advantageous for lightweight and wearable applications such as smart masks. Beyond structural integration, system architecture also includes embedding essential electronic components such as a microcontroller unit (MCU), signal conditioning circuitry, wireless communication modules, and power management systems. These components must be compact, lightweight, and mechanically stable while maintaining user comfort and breathability. Energy management represents one of the most critical integration challenges in wearable systems. The rechargeable battery is typically the heaviest single component of the mask and directly affects overall comfort. Excessive power consumption from continuous sensing and wireless transmission necessitates larger battery capacity, which increases weight and reduces wearability. Therefore, optimizing duty cycles, sampling frequency, and power regulation strategies is essential to maintain a lightweight design while ensuring extended operational lifetime [125].

### 3.2 Data Communication Architecture

Once sensor data is acquired and preprocessed by the MCU, it must be transmitted to the user in real time to enable immediate feedback. Real‐time connectivity ensures that users receive instant alerts if abnormal physiological parameters or environmental hazards are detected. The selection of an appropriate wireless protocol is a critical design decision that directly affects power consumption, transmission range, and overall ergonomics. Early‐stage prototypes often employ Wi‐Fi due to its high data throughput and straightforward implementation; however, because smart masks typically transmit low‐bandwidth telemetry (such as temperature, humidity, gas concentration, and respiration patterns), this high throughput is generally unnecessary. Furthermore, Wi‐Fi is energy‐intensive and significantly reduces battery life in compact wearables. For advanced prototypes and commercial implementations, BLE [90] represents a more suitable communication protocol. By operating in low‐duty‐cycle modes and transmitting small data packets at optimized intervals, BLE provides sufficient bandwidth for periodic telemetry while drastically reducing power consumption compared to Wi‐Fi. This transition to BLE enables continuous real‐time monitoring with minimal battery drain, allowing for smaller battery capacity, reduced overall mask weight, and improved ergonomic performance without compromising connectivity. In specific scenarios requiring battery‐free operation or ultra‐short‐range communication, Near‐Field Communication (NFC) can also be employed, though its limited range and intermittent activation restrict continuous monitoring [126]. Furthermore, the overall communication architecture must be designed to support either edge processing (data analysis on a smartphone) or cloud‐based processing, depending on latency requirements, privacy considerations, and computational demands. Thus, communication protocol selection is directly linked to system integration, data handling, and energy optimization, playing a central role in achieving a practical and lightweight smart mask platform. Thus, communication protocol selection is directly linked to system integration and energy optimization, playing a central role in achieving a practical and lightweight smart mask platform.

### 3.3 AI‐Driven Data Processing

While accurate sensing and efficient data transmission are fundamental, the true value of a smart mask lies in intelligent data interpretation. Continuous streams of real‐time telemetry provide a rich dataset that can be leveraged using artificial intelligence (AI) and machine learning algorithms. Rather than merely displaying raw measurements, AI models can analyze multisensor data to generate actionable and personalized insights. By continuously monitoring internal temperature, humidity, respiration dynamics, and environmental exposure, the system can establish an individualized physiological baseline for each user. Machine learning‐based anomaly detection algorithms can then identify deviations from this baseline, enabling early warnings of respiratory distress, abnormal breathing patterns, or potential fever conditions. Furthermore, predictive analytics can be applied to long‐term usage and environmental data to estimate filter degradation or mask performance decline. Instead of relying on fixed replacement schedules, the system can intelligently notify users when maintenance or filter replacement is required, ensuring optimal protection while avoiding unnecessary replacement. Through data fusion, personalization, and predictive modeling, AI transforms the smart mask from a passive monitoring device into a proactive health and safety management system [126].

## 4 Mechanism

### 4.1 Shared Principles in Sensor Design

We begin by briefly reviewing key parameters that evaluate the efficiency and performance of sensors, followed by a concise overview of their shared fundamental operating principles. The first parameter is sensitivity, which indicates a sensor's accuracy in converting an input stimulus into an output signal. It is quantified by the formula *S* = d*P/*d*X*​, where *S* represents the relative change, in the output signal (*X*) in response to the applied stimulus (*P*).

Next, response time refers to the duration required for a sensor to achieve a stable output signal. A shorter response time correlates with better sensor performance, particularly in real‐time monitoring applications. Polymer‐based materials possess longer response time due to their viscoelastic property [127].

The range of detection defines the minimum and maximum levels of a target parameter that a sensor can reliably measure. While literature often highlights the limit of detection (the smallest detectable quantity), this review prioritizes the range of detection, as it is critical to understanding a sensor's operational boundaries before signals (like humidity, pressure, stress, etc.) saturation occurs.

Hysteresis is when a sensor gives a slightly different output for the same input, depending on whether the input is increasing or decreasing. Hysteresis is typically observed in sensors that rely on material deformation or magnetic properties, such as strain gauges or magnetic circuits, leading to small offsets over time that can affect accuracy [128].

Stability refers to a sensor's ability to resist external factors and continue functioning under specified conditions throughout its intended lifespan, which is a key requirement for flexible sensors since respiration‐related disease often require a prolonged monitoring.

Finally, linearity measures the degree to which a sensor's output follows a linear relationship with the input. It is assessed by the deviation of the output signal from the ideal linear regression line, reflecting the sensor's stability in specific applications [129].

Although all the sensors‘ types reviewed rely on diverse mechanisms yet share fundamental operating principles, which we will briefly outline. Each sensor type operates via distinct working principles; here, we focus on those integrated into face masks for respiration monitoring, including resistive and capacitive sensors for humidity, piezoresistive, capacitive and piezoelectric sensors for pressure, resistive and capacitive sensors for strain, and mostly semiconductor sensors for gas detection. There are also other types of sensors that operate based on the triboelectric mechanism, where two different materials come into contact and electrons are transferred. The triboelectric mechanism will be explained in more detail in its dedicated section. These working principles share a common framework, where a specific stimulus induces measurable changes in the sensor's output. Resistive sensors rely on variations in the electrical resistance of a sensing material in response to external stimuli. For instance, humidity sensors detect changes due to moisture absorption, while strain or pressure sensors respond to mechanical deformation. Capacitive sensors, on the other hand, utilize dielectric materials sandwiched between conductive electrodes, storing electrical energy as an electrostatic field. Their capacitance is described using the equation of *C*  =  ε0ε*rA*/*d*, where *ε*0​ (vacuum permittivity) and *εr*​ (relative permittivity of the dielectric) are constants, while *A* (electrode surface area) and *d* (dielectric thickness) dynamically change under stimuli like pressure or humidity. In the capacitance mechanism, even though the response is very linear, the sensitivity is low, especially when using materials with a high Young's modulus. Piezoresistive sensors are commonly used for pressure sensing. They work by turning mechanical deformation into changes in resistance, either through the material's natural piezoresistive effect or through other mechanisms like the microcrack propagation, changes in structure shape, or the tunneling effect in conductive materials. Piezoelectric sensors operate via the inherent properties of piezoelectric materials, where mechanical forces (bending, compression, or tension) reorient molecular dipoles, generating an electric charge proportional to the applied stimulus. These mechanisms, resistive, capacitive, piezoresistive, and piezoelectric, highlight the versatility of sensing technologies. Resistive and capacitive principles dominate humidity, strain, and pressure sensing, with resistive systems tracking resistance fluctuations and capacitive systems monitoring dielectric or geometric changes. Piezoresistive mechanisms excel in pressure detection through strain‐induced resistance shifts, while piezoelectric transduction directly links mechanical stress to electrical output. In the following sections, the main mechanisms of each sensor type will be discussed in detail, followed by a review of recent advancements in their development.

### 4.2 Humidity Sensor

Humidity refers to the presence of water vapor in air or other gases and can be measured in two main ways. Relative humidity (RH) expresses the ratio of the amount of water vapor to the maximum possible saturation at a specific temperature, while absolute humidity measures the mass of water vapor present in a given volume of air, independent of temperature, and is expressed in grams per cubic meter (g/m^3^). These measurements provide different insights into atmospheric moisture, however, in humidity sensor applications relative humidity is more preferred due to its simplicity and relevance in various human issue and environmental applications. It represents a direct indication of the current moisture level relative to saturation, making it essential for practical applications involving humidity sensors [130]. Expiratory air becomes humidified as it travels through the nasal passages and lungs. Monitoring these humidity changes can provide valuable health insights, indicating physiological stress, emotional distress, mental health conditions, physical activity, metabolic rates, and potential diseases [39]. Consequently, real‐time, noninvasive breath humidity tracking is essential. This need has driven the development of humidity sensors, crucial for continuous health monitoring. Their rapid response to humidity variations enables early detection and intervention. Advancing humidity sensor technology marks a significant step in proactive healthcare, offering a noninvasive biomarker for health assessment and disease prevention. Early attempts at respiration monitoring with humidity sensors struggled with efficiency, particularly due to slow response and recovery times. For instance, in a 1997 study, Tatara and Tsuzaki reported response times of 8 and 10 s for an electrical hygrometer with and without a membrane filter, respectively; these times are not short enough, and the prolonged recovery time made it difficult to capture a stable signal during inspiration [39, 131]. This highlights the importance of optimizing sensor performance for effective respiratory monitoring. Early humidity sensor designs also lacked the flexibility crucial for health applications.

Recent advancements in sensor materials and microfabrication techniques have enabled the development of highly efficient systems, leading to ultrafast humidity sensors with microsecond response times and with more flexibility, a significant leap in the practicality of respiratory monitoring. Next, we will discuss the mechanisms of these sensors, providing insights into material selection and fabrication techniques. The operation of these sensors relies on the proton‐hopping process, also known as the Grotthuss chain reaction, which occurs under varying relative humidity levels. In this process [132, 133, 134, 135], charge is dynamically transferred within the sensing materials in response to changes in humidity. The strength of the signal generated by a humidity sensor depends mainly on the number of water molecules present on the surface of the sensing materials. Therefore, the ability of the materials to attract and retain water (hydrophilicity) is crucial to their performance. Water molecules adhere to the active material surface in two stages: initially, the first layer binds chemically to surface defects or hydroxyl groups, and as the relative humidity increases, additional layers are adsorbed physically. In an electrostatic field, water molecules can ionize, creating hydronium ions that help transfer charge, which in turn influences the sensor's output [39]. Additionally, the adsorption and desorption of water molecules significantly impact sensor performance. To be effective, the active materials used in these sensors should have a high affinity for water and offer a large surface area for interaction. Derived from the previously explained process, flexible humidity sensors are commonly classified into electrical signal‐based sensors including resistive, capacitive, and impedance type depending on distinct measurement techniques. There are additional humidity sensor classifications, such as acoustic surface sensors, surface acoustic wave resonators, field‐effect transistors, and optical fiber humidity sensors. These have not been integrated into masks for respiration monitoring and therefore are not considered in this review paper. In the following sections, we explore recent advances in electrical signal‐based humidity sensors and their integration into smart face masks, highlighting their role in precise humidity detection for respiratory health monitoring.

#### 4.2.1 Resistive/Impedance Humidity Sensor

**FIGURE 3:**
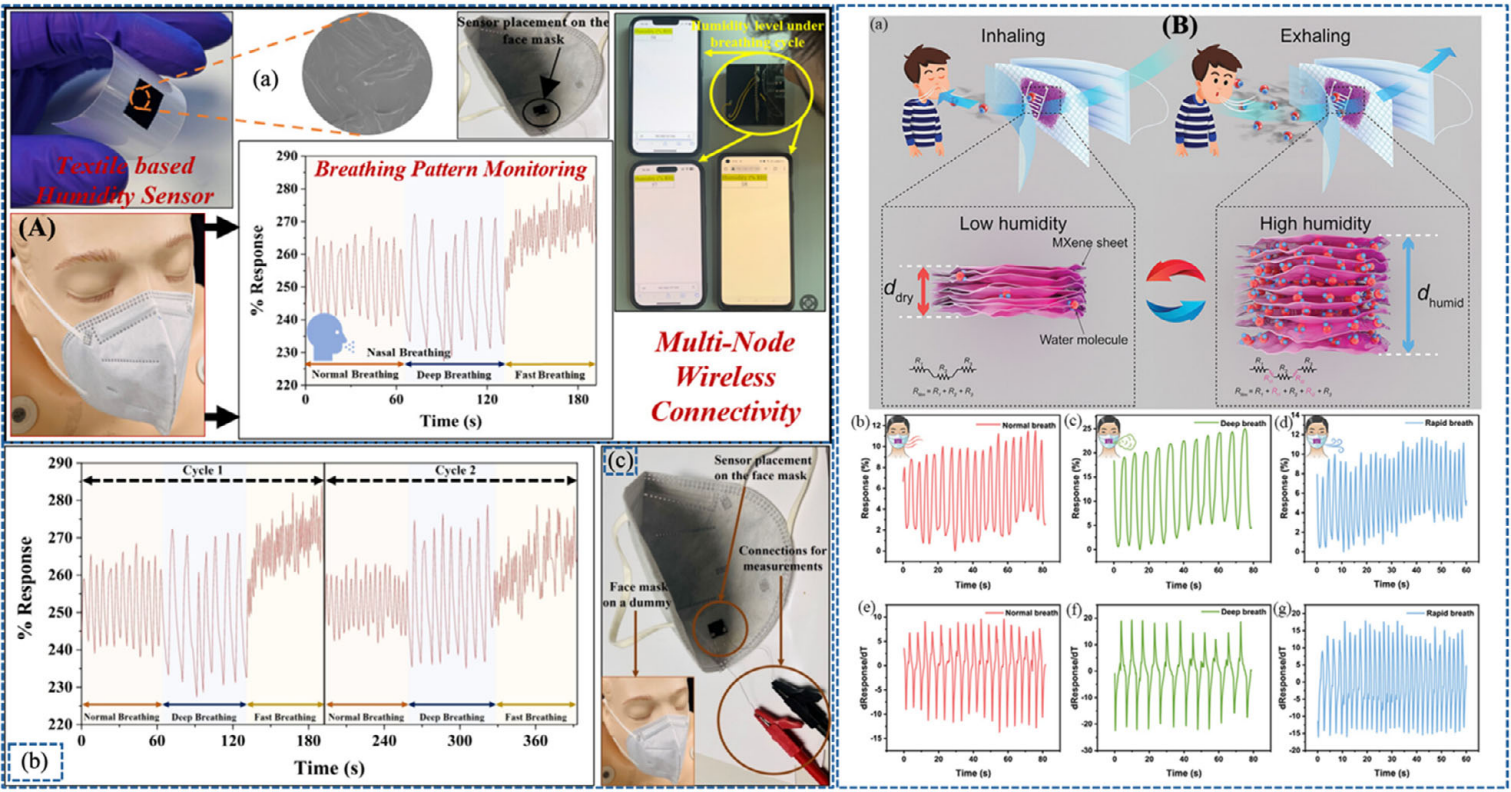
(A) Graphical abstract of eco‐friendly textile‐based wearable humidity sensor. Reproduced with permission [155]. Copyright 2024, American Chemical Society. (B) Infographic outline of MXene/TPU composite film for humidity sensing and human respiration monitoring. Reproduced with permission [156]. Copyright 2023, Wiley‐VCH GmbH.

Beniwal et al. [155] presented a cotton‐based resistive humidity sensor using PEDOT:PSS as the active layer, exhibiting a wide sensing range of 25%–91.5% RH suitable for healthcare and environmental monitoring. The cotton substrate is first treated with a Fabsil protector to enhance hydrophobicity and then dip‐coated with PEDOT:PSS. Compared with conventional sensing materials, PEDOT:PSS offers advantages such as biocompatibility, environmental friendliness, and facile processing. The sensing mechanism arises from the intrinsic core–shell structure of PEDOT:PSS, in which the conductive PEDOT core is surrounded by a hydrophilic PSS shell. Under humid conditions, water adsorption by the PSS shell induces swelling, increasing the separation between PEDOT‐rich domains and limiting charge carrier hopping, thereby increasing the electrical resistance. Such sensor is particularly promising for respiration monitoring, as well as skin moisture and neonatal care monitoring (Figure 3A).

Liu et al. [156] developed a MXene/TPU composite mat as a flexible humidity sensor for distinguishing breathing intensities and monitoring respiratory signals during various physical activities. The sensor is fabricated by electrospinning TPU fibers, modifying the mat with chitosan to promote electrostatic interaction, and subsequently depositing MXene nanosheets through solution soaking. The sensing mechanism is mainly governed by humidity‐induced changes in the interlayer spacing of MXene nanosheets. With increasing humidity, adsorbed water molecules expand the sheet‐to‐sheet distance, increasing the tunneling resistance at nanosheet junctions and thereby raising the overall sensor resistance. When humidity decreases, the interlayer distance contracts, leading to recovery of the electrical resistance. This humidity‐dependent tunneling effect directly influences sensor sensitivity and response, as small variations in nanosheet spacing significantly modulate electron transport pathways, enabling accurate detection of respiration‐related humidity changes (Figure 3B).

**FIGURE 4:**
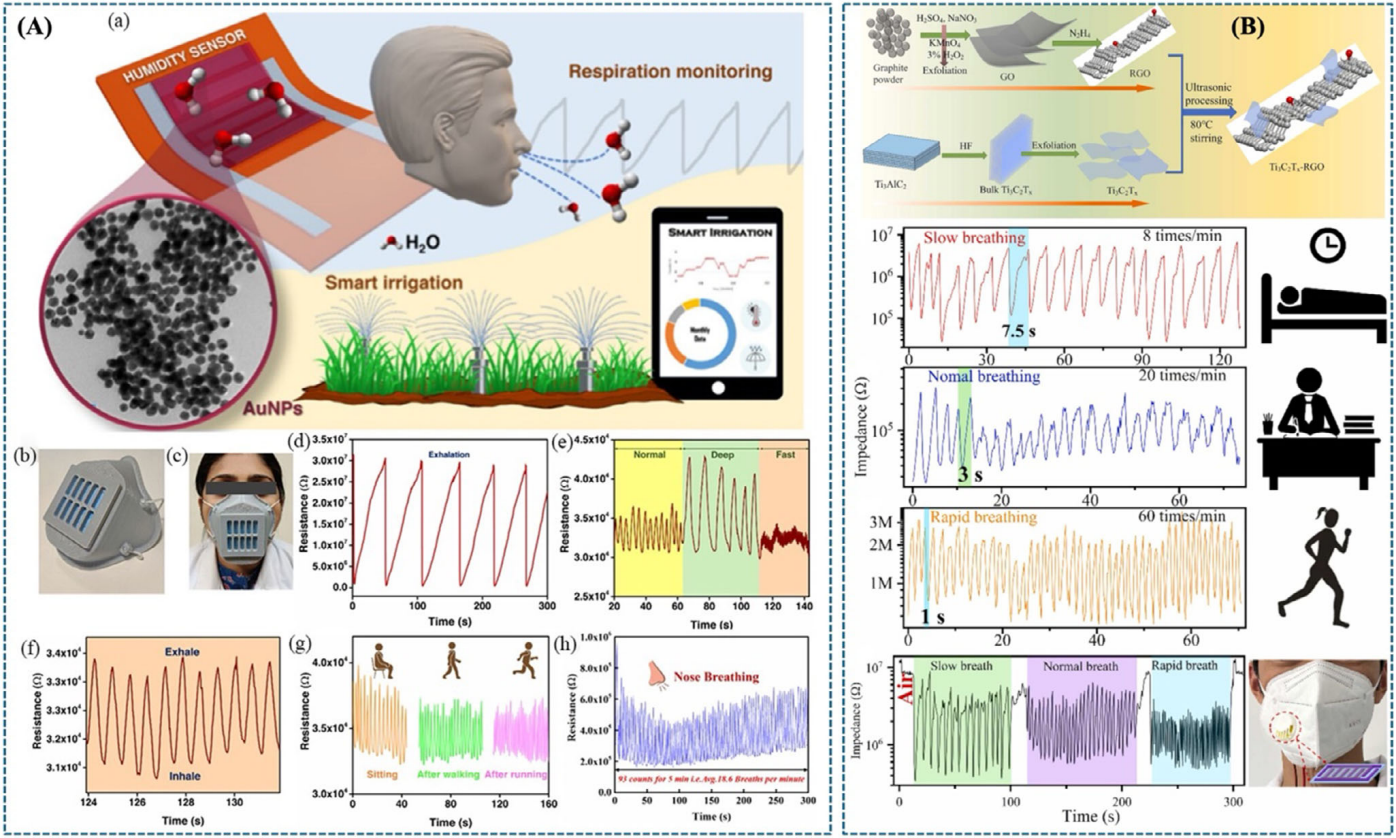
(A) Schematic mapping of gold‐nanoparticle‐based flexible humidity sensor for breath monitoring. Reproduced with permission [158]. Copyright 2024, American Chemical Society. (B) Illustration of highly sensitive MXene–RGO humidity sensor for human noncontact respiratory monitoring. Reproduced with permission [157]. Copyright 2023, Elsevier B.V.

To further enhance the humidity sensing performance of MXene‐based materials, Yu et al. [157] developed a Ti_3_C_2_T*_x_* MXene–RGO composite humidity sensor. In contrast to the MXene/TPU composite, where the sensing response mainly arises from macroscopic, humidity‐induced swelling, this approach works at the nanoscale. The insertion of RGO between the MXene layers expands the interlayer spacing and prevents restacking. This newly created 3D structure effectively exposes the abundant surface functional groups (─O, ─OH, ─F) intrinsic to MXene, allowing them to act as highly active adsorption sites for water molecules. Once adsorbed, these water molecules facilitate rapid proton hopping at low humidity and form multilayer structures that trigger the Grotthuss chain reaction at high humidity. Furthermore, the expanded spacing creates additional channels for water diffusion, facilitating much faster adsorption and desorption. As a result, the sensor exhibits high sensitivity, low hysteresis (∼1%), and extremely fast response/recovery times (7/16 s), enabling highly reliable respiratory monitoring (Figure 4B).

In contrast, Ramesh et al. [158] employ a conductive nanoparticle network by developing a flexible humidity sensor based on gold nanoparticles (AuNPs) for applications such as breath monitoring and smart irrigation. The sensor operates over a wide humidity range of approximately 40% RH to 95% RH and exhibits high sensitivity due to the conductive nanoparticle network. The sensing mechanism relies on chemisorption of hydroxides at low humidity and physisorption of water layers at higher humidity, enabling proton transport via the Grotthuss mechanism. These interactions cause measurable resistance changes that reflect the surrounding humidity level. Owing to the high surface area and conductivity of AuNP networks, the sensor demonstrates reliable detection of small humidity fluctuations, enabling real‐time monitoring of human respiration as well as environmental moisture levels. However, the high cost of AuNPs and the reduced sensitivity at elevated humidity levels may limit their practical scalability (Figure 4A).

**FIGURE 5:**
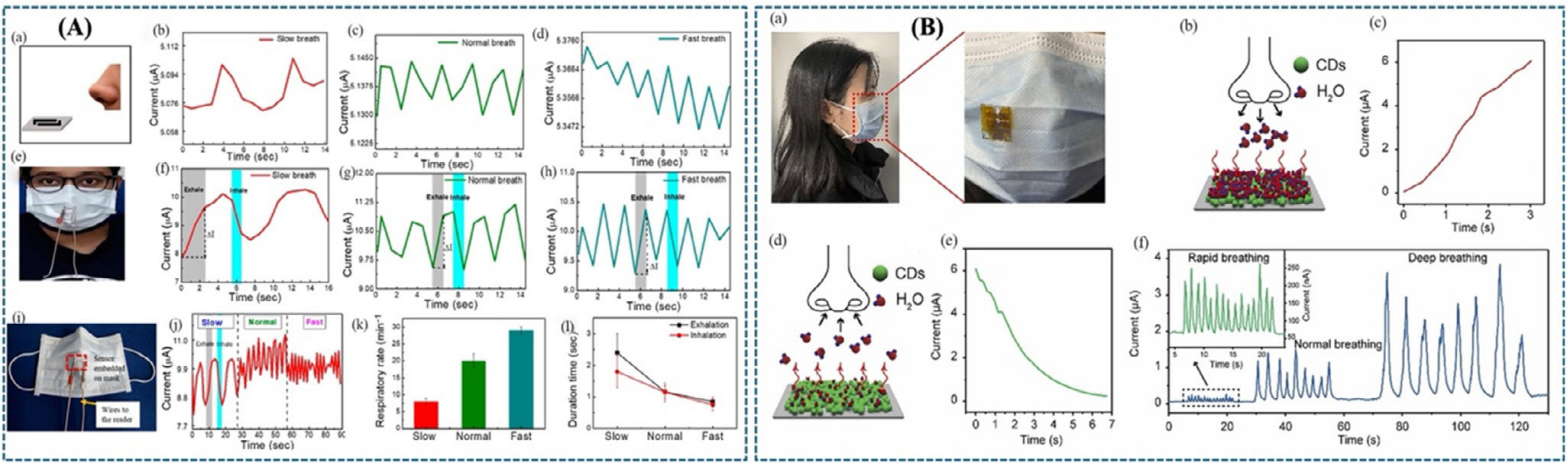
(A) Graphical abstract of SnO_2_‐based ultraflexible humidity/respiratory sensor. Reproduced with permission [159]. Copyright 2023, MDPI. (B) Pictorial representation of carbon nanodot‐based humidity sensor for self‐powered respiratory monitoring. Reproduced with permission [160]. Copyright 2022, Elsevier Ltd.

Alternatively, metal oxide semiconductors have also been explored for flexible humidity sensing. In this work [159], an ultraflexible humidity sensor based on SnO_2_ is fabricated on plastic wrap using a low‐temperature near‐infrared laser annealing process. The sensor operates in the 15%–70% RH range and can detect very small humidity changes. The sensing mechanism is mainly attributed to the adsorption of water molecules on the SnO_2_ surface, where abundant oxygen vacancies and hydroxyl groups act as active sites for water adsorption and dissociation. This interaction facilitates proton transport on the surface, resulting in measurable resistance changes with humidity. Owing to its ultrathin and flexible substrate, the sensor can be directly integrated into face masks for real‐time respiration monitoring (Figure 5A).

A triboelectric nanogenerator (TENG) humidity sensor developed by Qin et al. While the previously discussed flexible humidity sensors demonstrate high sensitivity and mechanical flexibility, they generally require an external power source to operate and measure resistance changes. To overcome this limitation, recent research has focused on integrating sensing systems with energy‐harvesting technologies to enable self‐powered operation. In this context, a fully self‐powered humidity sensor has been developed by combining carbon nanodots (CDs) with a breath‐driven TENG [160]. Similar to other carbon‐based nanomaterials, CDs contain abundant hydrophilic functional groups that readily adsorb water molecules, ensuring high humidity sensitivity. The core innovation lies in the device architecture, where the mechanical energy of human exhalation drives the TENG to generate its own electrical output (up to 200 V). This allows the sensor to autonomously monitor real‐time respiration and identify different breathing statuses without the need for any external batteries (Figure 5B).

**FIGURE 6:**
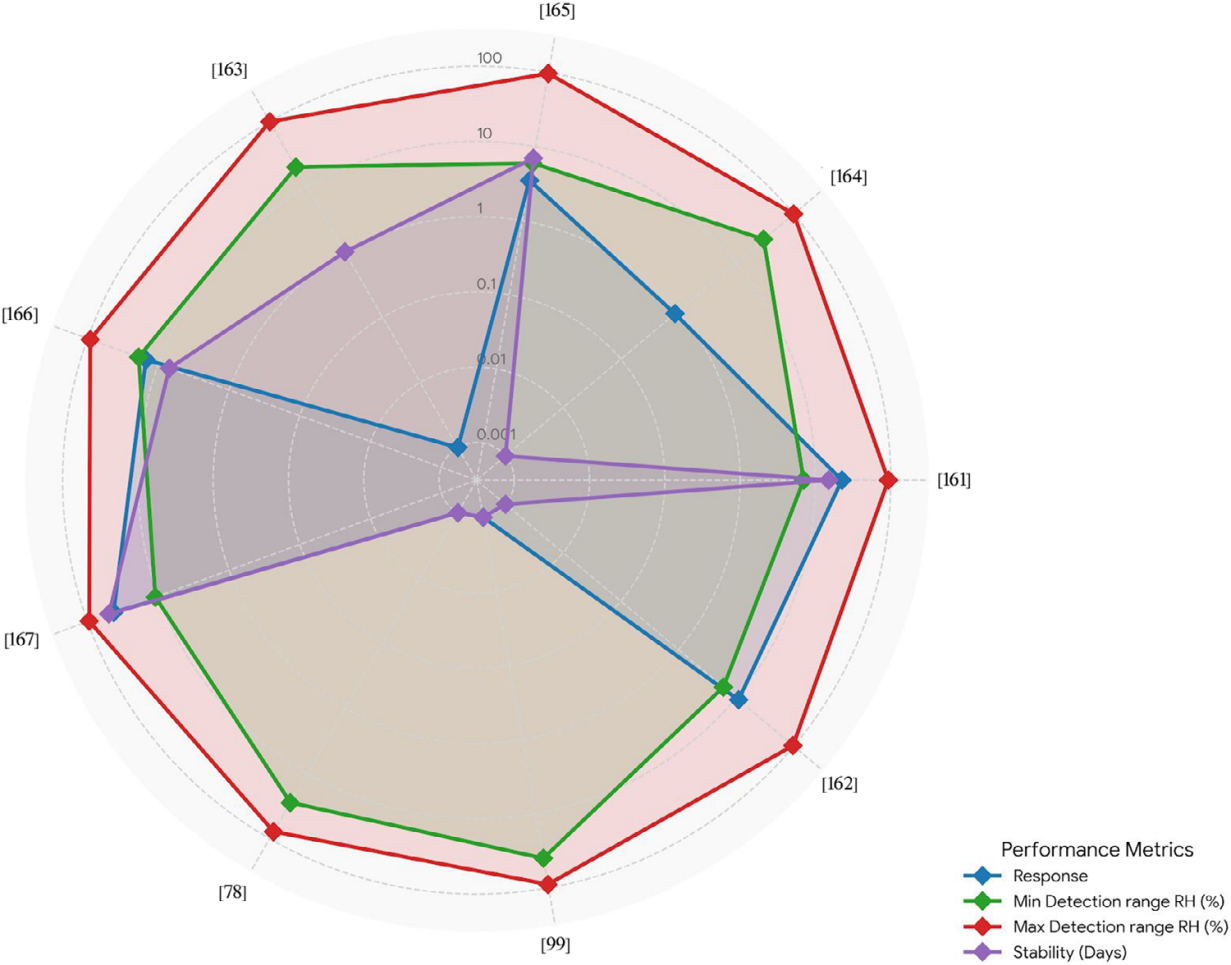
Radar chart of the capacitive‐based humidity sensors for four performance parameters, created by the authors using Microsoft Excel, based on Table 3 data.

While there are additional papers on the topic, we focus on the most recent ones in the main text. The remaining studies are summarized in Table 2, where they are sorted based on their sensitivity, from high to low (Figure 6).

**TABLE 2:** Resistive‐based humidity sensors sorted by sensitivity.

Active material	Sensitivity/responsivity	Response/recovery time (s)	Detection range RH%	Stability	Year
PVA–CNF organohydrogel [136]	25,000% at 98% RH	N/R	11–98	120 days	2022
Polyacrylamide (PAM)/Carrageenan [116]	13,462.1%/% RH	276/227	11–98	N/R	2022
Ag/Fe_3_O_4_ NWs [137]	6600% (∆I/I0) at 95%	N/R	11–95	N/R	2020
CNT/PANI [138]	42.6% at 90% RH	23/120	10–90	N/R	2019
Polyamide (PA) 66 fiber [139]	34.50%	N/R	10–90	N/R	2021
Sodium polystyrene sulfonate/citric acid [140]	2.15%/RH in 50–90% RH	3.8/7.26	50–90	N/R	2022
MXene (Ti_3_C_2_T*_x_*) ink [141]	0.601	17/37	11–97	N/R	2024
CNT/MXene [142]	N/R	28/66	10–90	200 cycles	2022
Polyester threads [143]	N/R	0.8/1.5	30–79	N/R	2022
Polyaniline [144]		2.1/2.8	N/R	N/R	2022
Halide perovskite Cs_2_TeBr_6_ [145]	N/R	4/253	11–97	N/R	2022
PEDOT‐Cl [146]	N/R	N/R	N/R	N/R	2021
ZnO/graphite carbon nitride (g‐C_3_N_4_) [147]	N/R	22/5	11–95	30 days	2021
Cellulose/KOH [148]	N/R	6/10.8	11.3–97.3	N/R	2020
Polyethylene glycol (PEG)/gold nanoparticle [149]	N/R	1.2/3	1.8–95	N/R	2019
Polyurethane (PU)/acidified CNT [150]	N/R	N/R	11–95	N/R	2019
Leather [151]	N/R	N/R	33–90	N/R	2019
Paper [152]	N/R	472/19	7.2–91.5	N/R	2019
Ag/alginate nanofibers [153]	N/R	1.4/1.1	20–85	N/R	2018
Polydopamine/graphene oxide [154]	N/R	20/17	0–97	N/R	2018

#### 4.2.2 Capacitive Humidity Sensors

**FIGURE 7:**
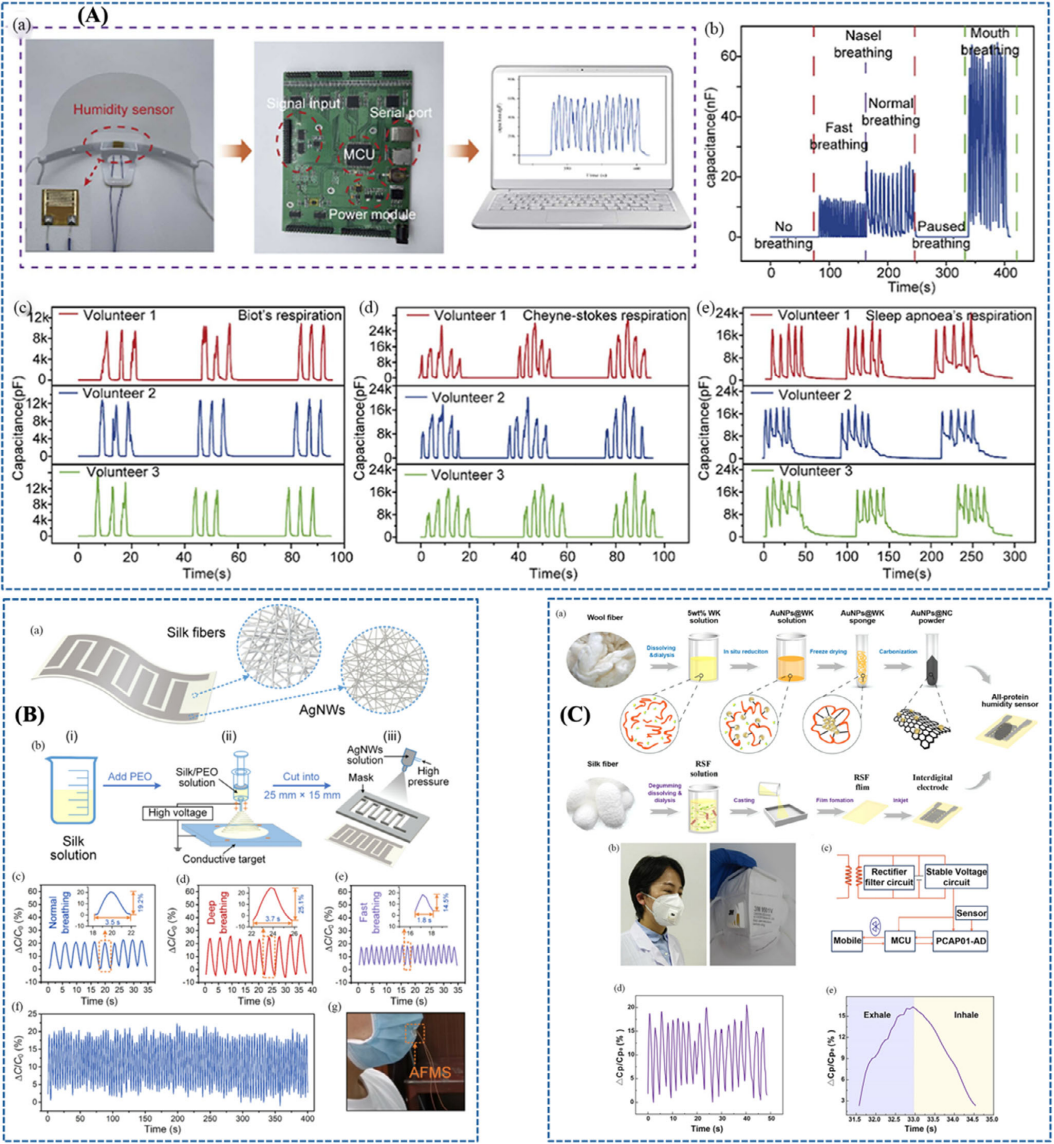
(A) Schematic representation of capacitive humidity sensor based on flower like SnS_2_/Ti_3_C_2_ MXene for respiration monitoring. Reproduced with permission [161]. Copyright 2024, Elsevier B.V. (B) Infographic of capacitive humidity sensor based on all‐protein imbedded with gold nanoparticles in carbon composite for human respiration detection. Reproduced with permission [162]. Copyright 2021, IOP Publishing. (C) Graphical illustration of silk fibroin‐based wearable all‐fiber sensor fabrication and its performance. Reproduced with permission [163]. Copyright 2022, Springer Nature.

Jian et al. [161] developed a high‐performance capacitive humidity sensor based on a flower‐like SnS_2_/Ti_3_C_2_ MXene nanocomposite, synthesized via an in situ hydrothermal method. The hierarchical structure provides a large surface area and abundant adsorption sites for water molecules, while MXene enhances charge transport. As humidity increases, water adsorption alters the dielectric properties of the sensing layer, leading to a significant increase in capacitance. As a result, the sensor exhibits a wide detection range of 7%–93% RH, with a capacitance response several orders of magnitude higher than that of pure SnS_2_, enabling applications such as respiration monitoring and noncontact humidity detection (Figure 7A).

Similarly, bio‐derived materials have also been explored to develop flexible humidity sensors for respiration monitoring. Ma et al. [162] reported a capacitive humidity sensor based on an all‐protein substrate embedded with gold nanoparticles within a nitrogen‐doped carbon matrix (AuNPs@NC). The device is fabricated on a flexible silk‐protein substrate with interdigital electrodes, while the sensing layer is obtained by dispersing gold nanoparticles in a wool‐keratin‐derived porous carbon precursor. The porous structure and abundant hydrophilic functional groups promote rapid adsorption of water molecules, which changes the dielectric properties of the sensing layer and leads to measurable capacitance variations. As a result, the flexible sensor demonstrates high sensitivity and reliable performance for real‐time respiration monitoring, benefiting from the biocompatibility and flexibility of protein‐based materials. The protein‐derived AuNP@NC sensor offers superior biocompatibility and flexibility, making it ideal for wearable applications, but it may face challenges in scalability, long‐term stability (Figure 7B).

Wen et al. [163] reported a wearable all‐fiber multifunctional sensor based on silk fibroin with integrated Ag nanowire interdigital electrodes for smart clothing applications. In this design, silk fibroin serves both as a flexible substrate and a functional humidity‐sensitive dielectric material. The all‐fiber structure provides ultrahigh flexibility, excellent air permeability, and biocompatibility, while the environmentally sensitive dielectric response of silk fibroin enables simultaneous detection of pressure, temperature, and humidity, with focus on humidity sensing for breath monitoring. Humidity changes modulate the dielectric properties of the silk fibroin film, producing measurable capacitance variations, which have been demonstrated for applications including smart masks for breathing monitoring and adaptive wearable systems. The multifunctional sensor also shows good mechanical reliability and stable sensing behavior under deformation. Compared with the protein‐derived AuNP@NC capacitive sensor, the silk fibroin device offers enhanced multifunctionality and improved breathability (Figure 7C). The remaining capacitive‐based sensors are summarized in Table 3, based on their sensitivity.

**TABLE 3:** Capacitive‐based humidity sensors sorted by sensitivity.

Active material	Sensitivity/responsivity	Response/recovery time (s)	Detection range RH (%)	Stability	Year
SnS_2_/Ti_3_C_2_ MXene [161]	433,827.42% at 1 kHz	22.5/0.21	7–93	15 days	2025
Silver (Ag) ink [164]	128 pF at 30% RH, 4400 pF at 100% RH	0.87/1.2	30–100	N/R	2024
Functional yarn [165]	82.4 pF/%RH	3.5/4	6–97	1000 cycles, in 7 days	2019
Silk fiber film [163]	7.5%/kPa	N/R	20–100	400 s	2022
ZnO–PVDF/polyethylene glycol (PEG) [163]	0.7 pF/RH%	15/7	19–92	7 days	2022
Silk yarn coated with PI [167]	∼0.25 at 500 Hz (pF/% RH)	43/26	11–95	50 days	2020
Polyester threads [78]	0.116 at 1 kHz/0.052 at 100 kHz	N/R	28–78	N/R	2021
Nanoforests [99]	0.014(10%–40% RH)/0.11(40%–90% RH)	–/5	40–90	N/R	2022
Golds NP in a carbon precursor [162]	N/R	11/43	6–97	N/R	2022

In conclusion, ultrafast humidity sensors offer several advantages for respiration monitoring. They are typically noninvasive, lightweight, and capable of detecting breathing patterns with high speed and accuracy. These features make them promising tools for real‐time tracking of respiratory rate and rhythm. However, there are still challenges to address. One key limitation is the tradeoff between fast response/recovery times and sensitivity, which can affect the sensor's overall performance. Additionally, while early studies show potential, most work has been limited to small‐scale feasibility tests. To fully unlock their practical use, not just in respiration monitoring but also in areas like voice recognition, touchless interfaces, and skin analysis, broader trials involving diverse human subjects are essential.

### 4.3 Pressure Sensor

Pressure is a fundamental physical parameter that quantifies the force exerted per unit area. This is described mathematically by the formula: *P*  =  *F*/*A*, where *P* represents pressure, *F* represents the force applied, and *A* is the area over which the force is spread. Pressure is commonly measured in Pascals [168], which is equivalent to one newton per square meter [N/m^2^]. The concept of pressure arises from the interaction of forces over an area, playing a crucial role across a wide range of natural and engineered systems, from weather patterns to hydraulic presses. An increase in the force *F* or a decrease in the area *A* will result in higher pressure, and vice versa. Pressure is a scalar quantity, meaning it has magnitude but no direction. It acts equally in all directions at any point within a confined fluid. This characteristic makes pressure ideal for measurement using sensors, which detect variations in pressure to monitor various systems. A pressure sensor, as a technological application of this concept, is designed to measure and report the pressure of its subject [169]. These sensors primarily function by converting applied pressure into an analog or digital signal, which can then be interpreted and used by other devices or systems. Pressure measurement is crucial in fields such as meteorology, aviation, healthcare, and more. In healthcare, for example, pressure sensors can monitor blood pressure or respiratory patterns. With advancements in technology, pressure sensors have become more accurate, durable, and compact, capable of enduring extreme conditions and fitting into smaller, more complex systems. One of the emerging trends is the development of flexible pressure sensors, which have diverse applications, including object detection, structural monitoring, and gait analysis. More recently, attention has turned to the production of highly sensitive pressure sensors for monitoring respiration. These sensors are expected to play a key role in continuous respiration monitoring and could assist in the early detection of respiratory failures [167, 170]. To accurately detect various rates of respiration, pressure sensors must be highly sensitive and capable of measuring pressures in the 0–2 kPa range. These advancements have expanded the potential applications of pressure sensors, particularly in the development of smart masks for monitoring human respiration. A pressure sensor can monitor respiration by detecting the changes in air pressure that occur with each breath. When placed in a location that effectively captures pressure changes, such as a smart mask or wearable device, the sensor can monitor inhalation and exhalation through pressure variations. The working principle of the pressure sensor in this context is simple: as the person inhales, the air pressure within the mask or device decreases. This pressure drop causes the diaphragm of the sensor to deflect, altering its resistance, capacitance, or another characteristic property (depending on the sensor type). This change is measured and converted into an electrical signal. The opposite occurs during exhalation, when the pressure inside the mask or device increases, causing the diaphragm to deflect in the opposite direction and produce a corresponding signal. By measuring these pressure changes and the timing of each cycle, the sensor can provide valuable data on a person's breathing rate and depth, which can be tracked and analyzed for health purposes. Flexible pressure sensors integrated into smart masks offer notable advantages for respiration monitoring, including high sensitivity, comfort, and real‐time response to subtle breathing patterns. Their flexibility and lightweight design make them ideal for wearable applications. However, they can be affected by facial motion, humidity, and external mechanical noise, which may reduce accuracy. Ensuring durability and consistent performance in real‐world conditions remains a key challenge.

In the realm of pressure sensing technologies, our discussion will cover three primary categories: capacitive, piezoelectric, and piezoresistive pressure sensors, which are predominantly integrated into smart face masks, along with recent advancements in these technologies, with details presented in Tables 4 and 5, respectively.

**TABLE 4:** Summary of piezoresistive based pressure sensors sorted based on response/recovery time.

Active material	Sensitivity (kPa^−1^)	Response/recovery time (ms/ms)	Detection range (kPa)	Stability (cycles)	Year
BP‐AuNCs [178]	0.372	24/32	0–1.5	4000	2022
PDMS‐wrapped CNT arrays [179]	GF = 0.93	26/57	0.2–12	8000	2019
Graphene/MXene/cellulose [171]	−1.6189%	37.5/37.5	0–370	1500	2024
PEDOT:PSS/polyamide 6 [173]	6554.6	53/90	0–60	10,000	2022
PDMS micropillars array combined secondary nanoprotrusions [180]	26.08 Ω	56/140	0–32	1000	2024
CNTs [181]	0.2	70/50	0–45	5000	2021
MXene/silver nanowires [172]	770.86–1434.89	70/81	0–100	5000	2023
Carbonized cellulose fabric [174]	32.13	81/79	0–100	1000	2022
MXene [53]	509.5	150/60	0.5–100	10,000	2021
Ti_3_C_2_T*_x_*/NiSe_2_ [178]	2.41	220/200	1.477–3.185	3000	2021
PANI [182]	179.1	N/R	0–50	10,000	2022

**TABLE 5:** Summary of capacitive‐based pressure sensors.

Active material	Sensitivity (kPa^−1^)	Response/recovery time (ms/ms)	Detection range (kPa)	Stability (cycles)	Year
PVDFNM/polyurethane NM [183]	0.46 V/kPa^−1^	< 26	0–294	7000	2018
MXene/(PVP) [176]	1.25	30/15	0–294	10,000	2021
LSG electrodes/PVP nanofiber dielectric [184]	4.352	40/37	0–2	6000	2023
HM‐PDMS M‐tooth structure [185]	0.0246	40/180	0–40	1000	2021
PVP NMs [177]	0.278	41/36	0–200	8000	2022
BP‐AuNCs and Ecoflex sponge [186]	0.10@0–2 kPa, 0.06@2–7 kPa, and 0.01@7–12 kPa	45/60	0.1–12	> 5000	2024
Magnetically grown microneedles [187]	0.16	49/51	0–294	9000	2020
Porous PDMS [175]	0.18	100/100	0–400	10,000	2021

#### 4.3.1 Piezoresistive Pressure Sensors

**FIGURE 8:**
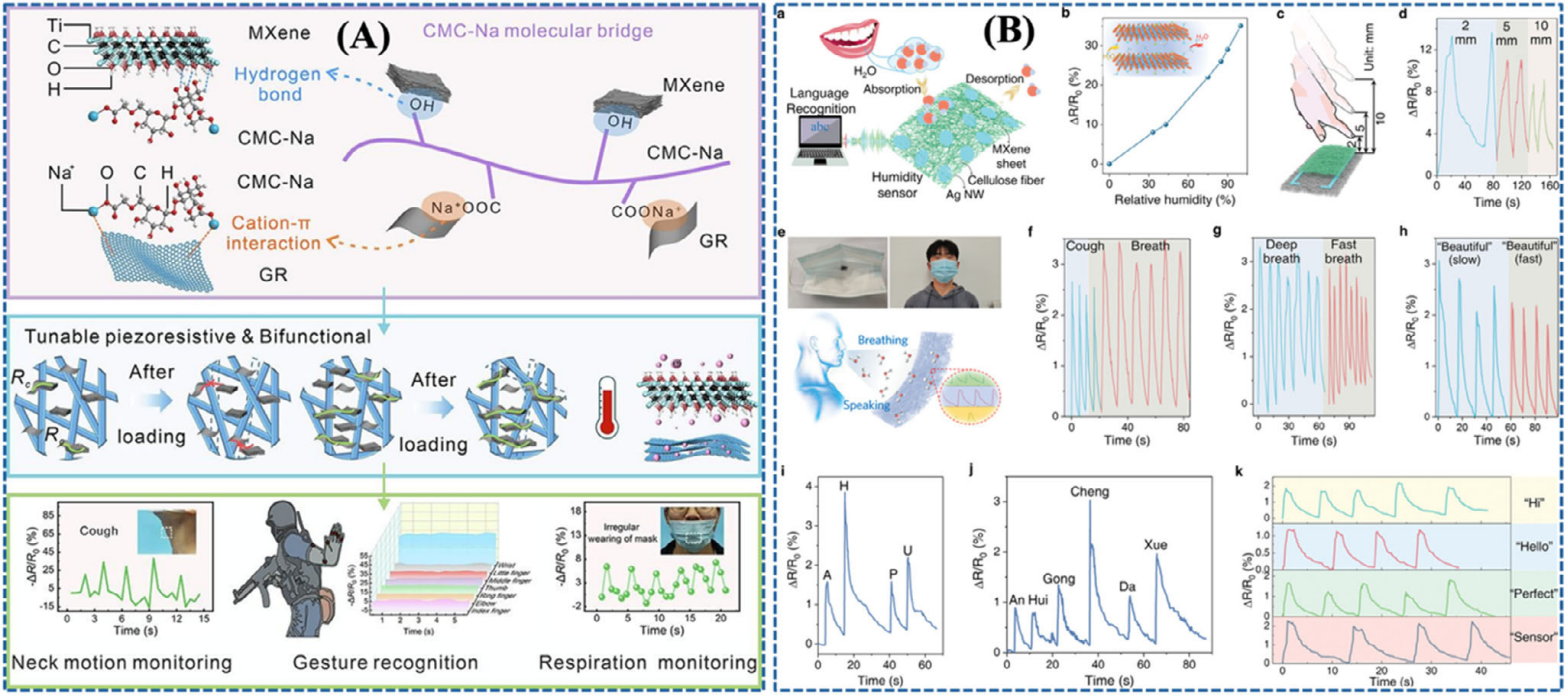
(A) Graphical abstract of graphene/MXene/cellulose paper‐based sensor with tunable piezoresistive effect for pressure sensing. Reproduced with permission [171]. Copyright 2024, Elsevier B.V. (B) Pictorial outline of highly breathable and sensitive pressure sensors based on functionalized MXene with multilayered porous structure. Reproduced with permission [172]. Copyright 2023, Wiley‐VCH GmbH.

Zhang et al. [171] developed a paper‐based bifunctional piezoresistive sensor (CP@GM) by integrating graphene and MXene within a CMC‐modified cellulosic matrix, where hydrogen bonding and cation interactions promote uniform filler dispersion and strong interfacial coupling within the fibrous substrate. This material design stabilizes the conductive network and allows pressure to modulate resistance through changes in the effective conductive filler distribution density, thereby improving signal reliability under deformation. As a result, the sensor combines a wide operating range up to 370 kPa with low‐cost, disposable mask compatibility, making it a practical platform for sleep‐breathing monitoring in surgical masks (Figure 8A).

With a stronger emphasis on breathability and weak‐pressure detection, Zheng et al. [172] designed a flexible piezoresistive sensor based on multilayer porous nonwoven fabrics, in which MXene interdigitated electrodes and MXene/silver nanowire‐coated sensing layers form a highly air‐permeable textile like conductive architecture. The porous multilayer structure increases compressibility and contact‐area variation under small external loads, which amplifies resistance changes and explains the markedly enhanced sensitivity and ultralow detection limit of about 1 Pa. Compared with the CP@GM paper platform, this design more effectively captures subtle respiratory and speech‐related signals, although within a narrower pressure window (Figure 8B).

**FIGURE 9:**
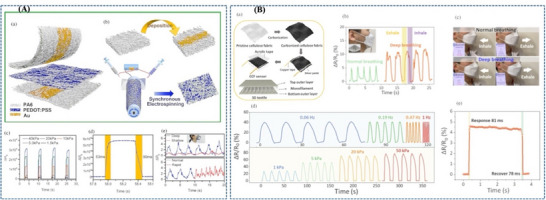
(A) Illustration of PEDOT:PSS/PA6 nanofiber network structure for ultrasensitive piezoresistive pressure sensors. Reproduced with permission [173]. Copyright 2022, American Chemical Society. (B) Schematic mapping of carbonized cellulose fabric‐based piezoresistive pressure sensor for human healthcare monitoring. Reproduced with permission [174]. Copyright 2022, Elsevier B.V.

Further advancing low‐pressure respiratory sensing, Zhou et al. [173] reported an ultrathin electrospun pressure sensor composed of Au‐coated PA6 nanofiber electrodes and a PEDOT:PSS/PA6 composite nanofiber sensing layer. In this system, the interwoven nanofiber network creates highly deformable and efficiently percolated conductive pathways, so even weak external pressure produces a pronounced resistance change. This mechanism enables an ultrahigh sensitivity of in the 0–1.4 kPa range, making the device especially effective for detecting subtle nasal breathing patterns when mounted on a KN95 mask. Relative to the paper‐based and porous nonwoven sensors, this nanofiber architecture is more specialized for very small respiratory pressures, although its optimal performance is confined to a limited pressure range (Figure 9A).

**FIGURE 10:**
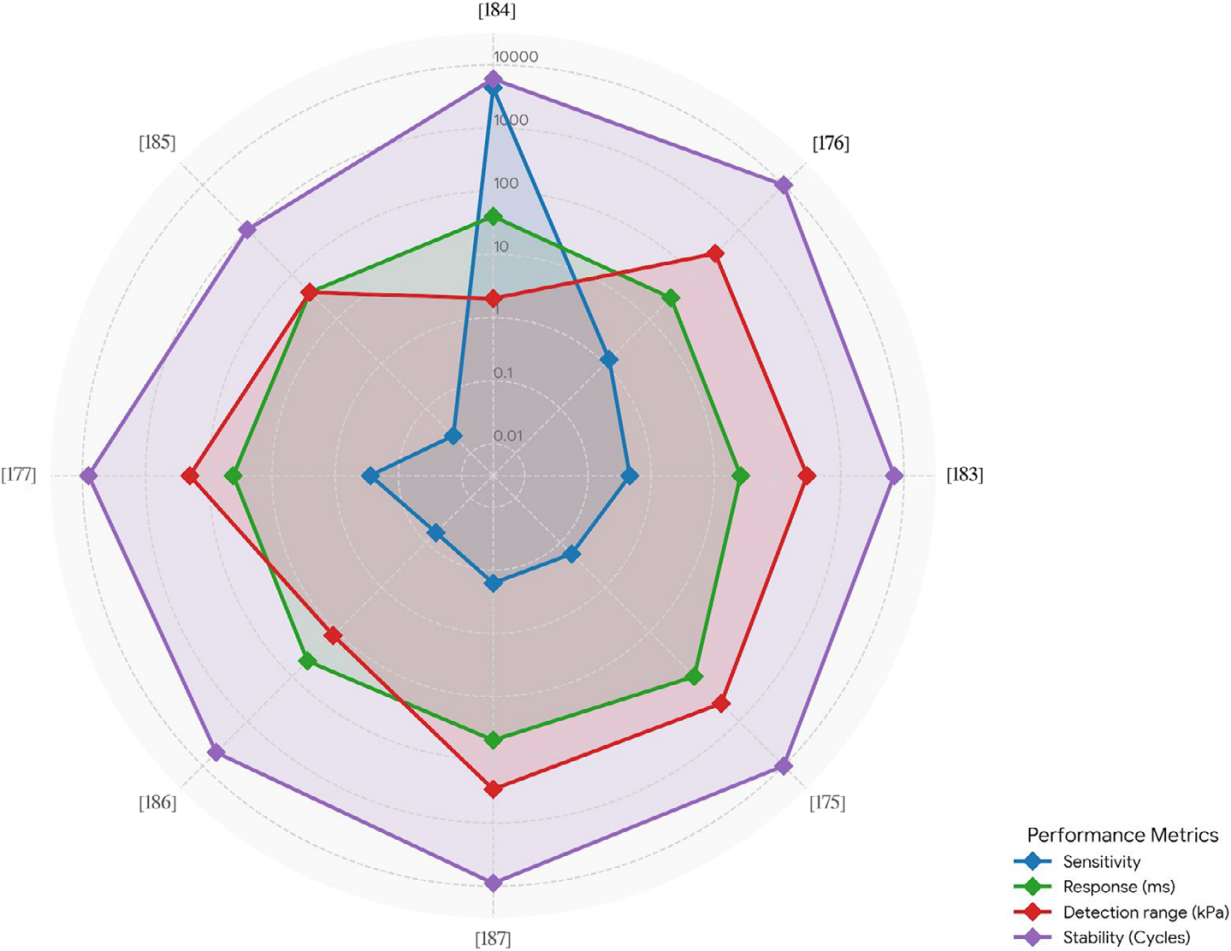
Radar chart of the capacitive pressure sensors for four performance parameters, created by the authors using Microsoft Excel, based on Table 5 data.

In contrast, Choi et al. [174] focused on structural robustness by integrating carbonized cellulose fabric with a prestrained viscoelastic monofilament to construct a 3D textile network. Here, the monofilament acts as a strain‐amplifying element that concentrates local deformation onto the conductive fabric, increases fiber‐to‐fiber contact under pressure, and thereby strengthens the piezoresistive response. This strategy enhanced the sensitivity from 3.35 to while preserving excellent stability over 5000 loading–unloading cycles, highlighting its suitability for durable and repeated wearable respiration monitoring. Compared with the highly sensitivity‐driven porous and electrospun designs, this approach is less optimized for ultralow‐pressure detection but more attractive for long‐term mechanical reliability (Figure 9B) (Figure 10).

#### 4.3.2 Capacitive Pressure Sensors

**FIGURE 11:**
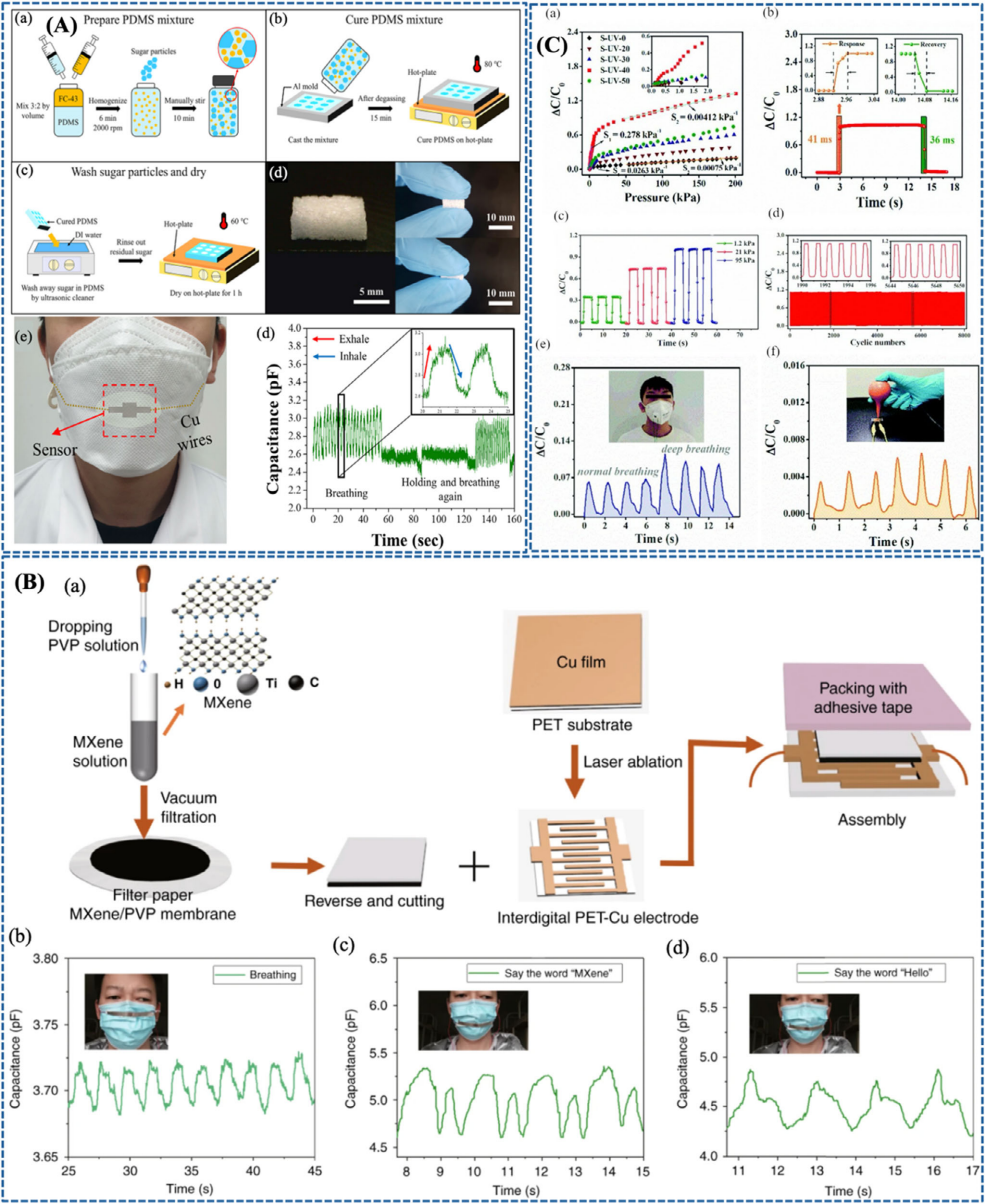
(A) Illustration of fabrication of hierarchically porous structured PDMS composites as a flexible capacitive pressure sensor. Reproduced with permission [175]. Copyright 2021, Elsevier B.V. (B) Graphical abstract of a new strategy for the fabrication of flexible and highly sensitive MXene/PVP‐based flexible pressure sensors. Reproduced with permission [176]. Copyright 2021, Springer Nature. (C) Schematic outline of biocompatible PVP nanofiber membrane via electrospinning and UV treatment for high‐performance capacitive pressure sensor. Reproduced with permission [177]. Copyright 2022, Royal Society of Chemistry (RSC).

Hwang et al. [175] developed a flexible capacitive pressure sensor based on hierarchically porous PDMS, where the main material innovation is the transformation of conventional dense PDMS into a multiscale porous dielectric layer. This porous architecture increases compressibility and enhances pressure‐induced changes in dielectric thickness and effective permittivity, thereby amplifying the capacitance response compared with bulk PDMS. As a result, the sensor achieved a 22.5‐fold sensitivity enhancement to while retaining a broad operating range of 0–400 kPa, indicating that porous dielectric engineering can improve sensitivity without sacrificing usable range (Figure 11A).

Moving from porous dielectric engineering to conductive composite design, Qin et al. [176] advanced capacitive sensing through an MXene/PVP material system combined with interdigital electrodes, where the high conductivity of MXene, the flexibility of PVP, and the intensified fringing electric field of the electrode geometry act synergistically to strengthen pressure‐induced capacitance modulation. This design therefore improves weak‐pressure signal transduction more effectively than porous PDMS alone, which is reflected in its higher sensitivity of about and ultralow detection limit of about 0.6 Pa. Such features make this architecture especially suitable for resolving subtle breathing signals in mask‐integrated applications. Compared with the porous PDMS sensor, this study more clearly prioritizes weak‐ pressure detectability and rapid dynamic response (Figure 11C).

In a related direction, Ren et al. [177] further emphasized dielectric material design by employing a biocompatible PVP nanofiber membrane, in which the nanofibrous network provides high deformability and abundant internal air gaps that facilitate efficient capacitance variation under compression. The sensing improvement therefore arises from the combined effects of fiber‐network compressibility and dielectric modulation, enabling a more wearable and skin‐compatible sensing platform. Although its low‐pressure sensitivity is lower than that of the MXene/PVP interdigital design, the device still delivered at 0–2 kPa and was successfully applied to mask‐based monitoring of normal and deep breathing, highlighting its balanced performance for practical wearable use (Figure 11B).

#### 4.3.3 Piezoelectric Pressure Sensors

**FIGURE 12:**
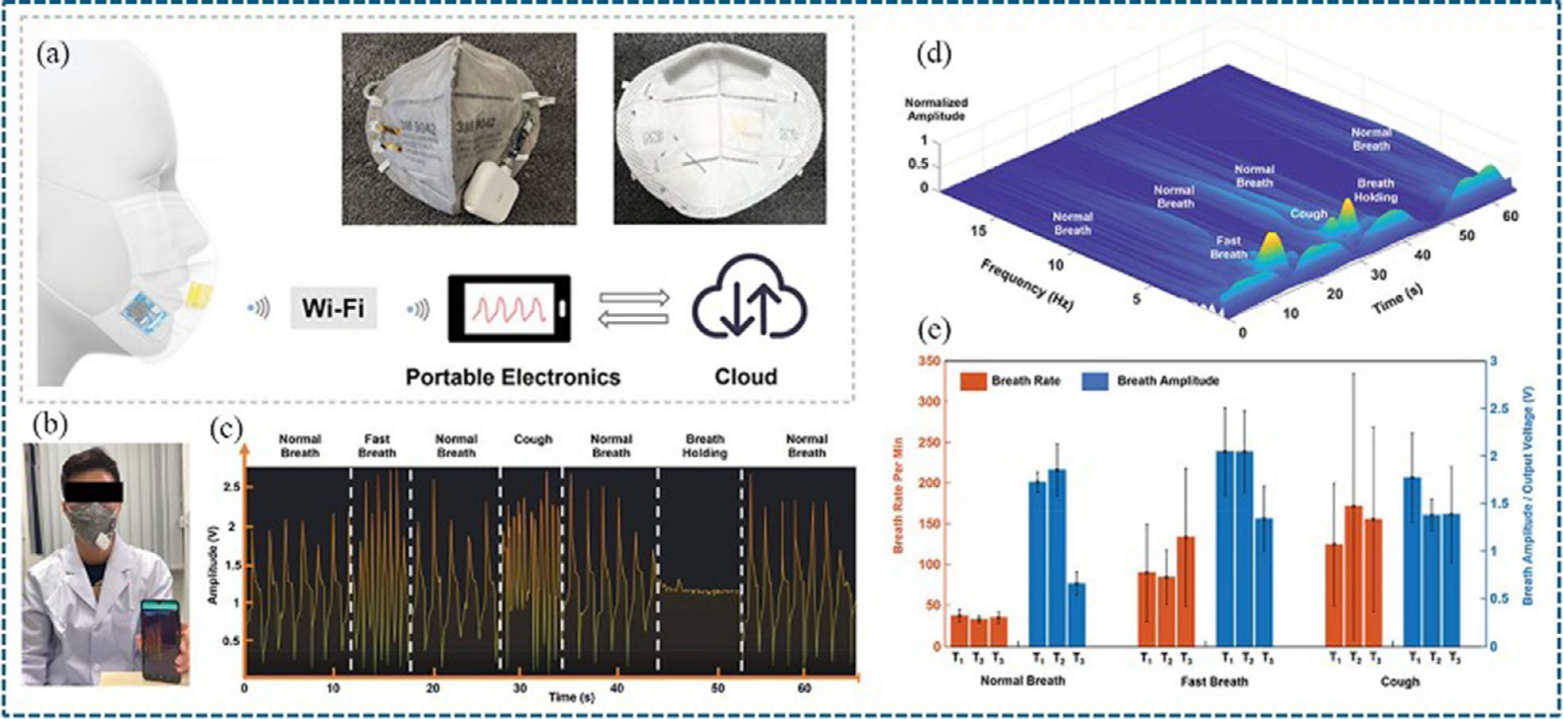
Schematic of smart face mask based on ultrathin piezoelectric pressure sensor for wireless breathing analysis. Reproduced with permission [188]. Copyright 2021, Wiley‐VCH GmbH.

**FIGURE 13:**
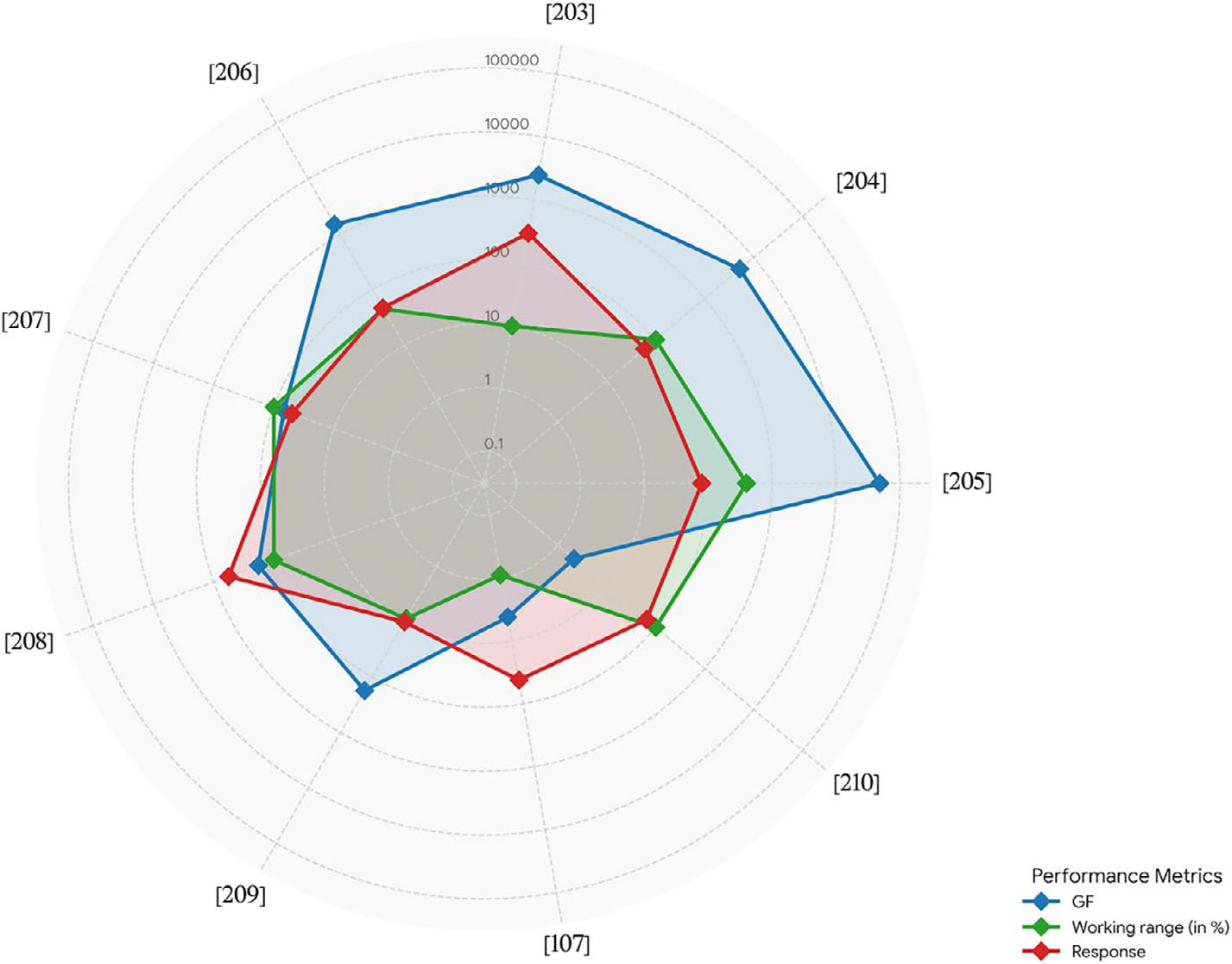
Radar chart of the strain sensors for three performance parameters, created by the authors using Microsoft Excel, based on Table 7 data.

Likewise, Zhong et al. [188] developed a self‐powered piezoelectric pressure sensor based on graphene oxide‐interfaced PVDF nanofibers, in which the introduction of GO into the PVDF nanofibrous matrix improved dipole alignment, interfacial polarization, and charge‐transfer efficiency, thereby strengthening the electromechanical response of the flexible sensing layer. This material coupling enabled a dual transduction mechanism, where mechanical deformation generated a piezoelectric output and temperature fluctuations induced a pyroelectric response, allowing the same platform to function as both a pressure and thermal sensor. As a result, the device achieved a high‐pressure sensitivity of up to 4.3 V/kPa, a low detection limit of 10 Pa, and output power densities of about under mechanical stimulation and under thermal fluctuations. When integrated onto an N95 mask, it enabled self‐powered monitoring of breathing and temperature, highlighting the advantage of piezoelectric nanogenerators over resistive and capacitive counterparts in combining sensing with intrinsic energy harvesting for multifunctional smart‐mask systems (Figure 12) (Figure 13).

### 4.4 Gas Sensor

Human breath contains a variety of gases and chemical compounds that can serve as indicators of both health status and environmental exposure. In 1971, Linus Pauling identified more than 200 different VOCs in exhaled breath. Monitoring the concentration of certain compounds, like oxygen or carbon dioxide, can provide insights into breath depth, rate, and pattern, offering valuable information about a patient's respiratory health. For individuals with chronic respiratory conditions like chronic obstructive pulmonary disease (COPD), asthma, etc., flexible gas sensors present new diagnostic and management possibilities. These sensors enable real‐time monitoring of respiratory activity, allowing for timely medical intervention, prevention of exacerbations, and improved symptom control. Gas sensors integrated into smart masks offer a noninvasive, portable solution for continuous health monitoring and air quality assessment. Gas sensors are commonly classified based on their detection mechanisms, including catalytic, electrochemical, optical, semiconductor, acoustic, and field‐effect transistor‐based sensors [189, 190].

Table 6 is a summary of all the gas sensors explained in this review. Given that a great number of gas sensors mounted on a face mask for respiratory monitoring, fall under the semiconductor classification, operating with resistance change principle, it would be advantageous to provide a concise overview of sensing mechanism of semiconductor gas sensors. Semiconductor metal oxide (SMO)‐based gas sensors rely on reversible reactions between gases and material surfaces. They are divided into n‐type and p‐type SMOs, depending on electron movement within the material. Common n‐type SMOs include SnO, Fe_2_O_3_, ZnO, and WO_3_, while NiO, CuO, and others are used for p‐type sensors [191]. The decoration of noble metals such as Os, Ir, Pt, and Rh, Ru, Rh, Pd, and Ag on these semiconductors can boost the sensitivity and selectivity of gas sensors.

**TABLE 6:** Summary of gas sensors sorted by their type.

Sensor type	Materials	Target gas	Response	Sensitivity	Response/recovery time (s)	Low detection limit (LOT)	Year
Chemiresistive [192]	Collagen‐ based fibers	Ammonia (NH_3_)	68.26%	N/R	159.3/–	800 ppb	2025
Semiconductor [195]	In_2_O_3_/ZnO	NO_2_	171.2 at 100 ppm	N/R	5/–	N/R	2025
Semiconductor [196]	Pb/WO_3_	Acetone	57.33 at 100 ppm acetone	8.22 at 1 ppm acetone	N/R	0.076 in the acetone range of 0.1–0.8	2024
Semiconductor [193]	PANI/WS_2_	Ammonia (NH_3_)	800 at 20 ppm NH_3_	N/R	108/229	8% at 100 ppb NH3	2024
Semiconductor [197]	PPy/MXene	Ethanol	76.3% at 400 ppm		49/18 s	2.21 ppm	2024
Semiconductor (metal–organic framework) [198]	MIL‐101 (Cr)/PDMS/PI	Acetone	Desorption Signal intensity	N/R	N/R	100 ppb to 2500 ppb	2024
			76.3				
			2.2 cm^2^/V·s	105			
Semiconductor [199]	Amorphous indium gallium zinc oxide	Respiratory modes indicator	Average mobility	On/off ratio	0.7 s	N/R	2024
			2.2 cm^2^/V·s	105			
Semiconductor [200]	PP/MXene/PANI	CO2	15.2 at 500 ppm CO_2_	N/R	N/R	N/R	2023
Semiconductor [25]	PP/CNT/PANI (prepopylene)	Ammonia (NH_3_)	425% at 70 ppm NH_3_	N/R	93/36	500 ppb	2023
Electrochemical (hydrogel based) [194]	PAM/CARR/Ecoflex elastomer	O2	N/R	0.335%/ppm	N/R	< 5 ppm O_2_	2023

**FIGURE 14:**
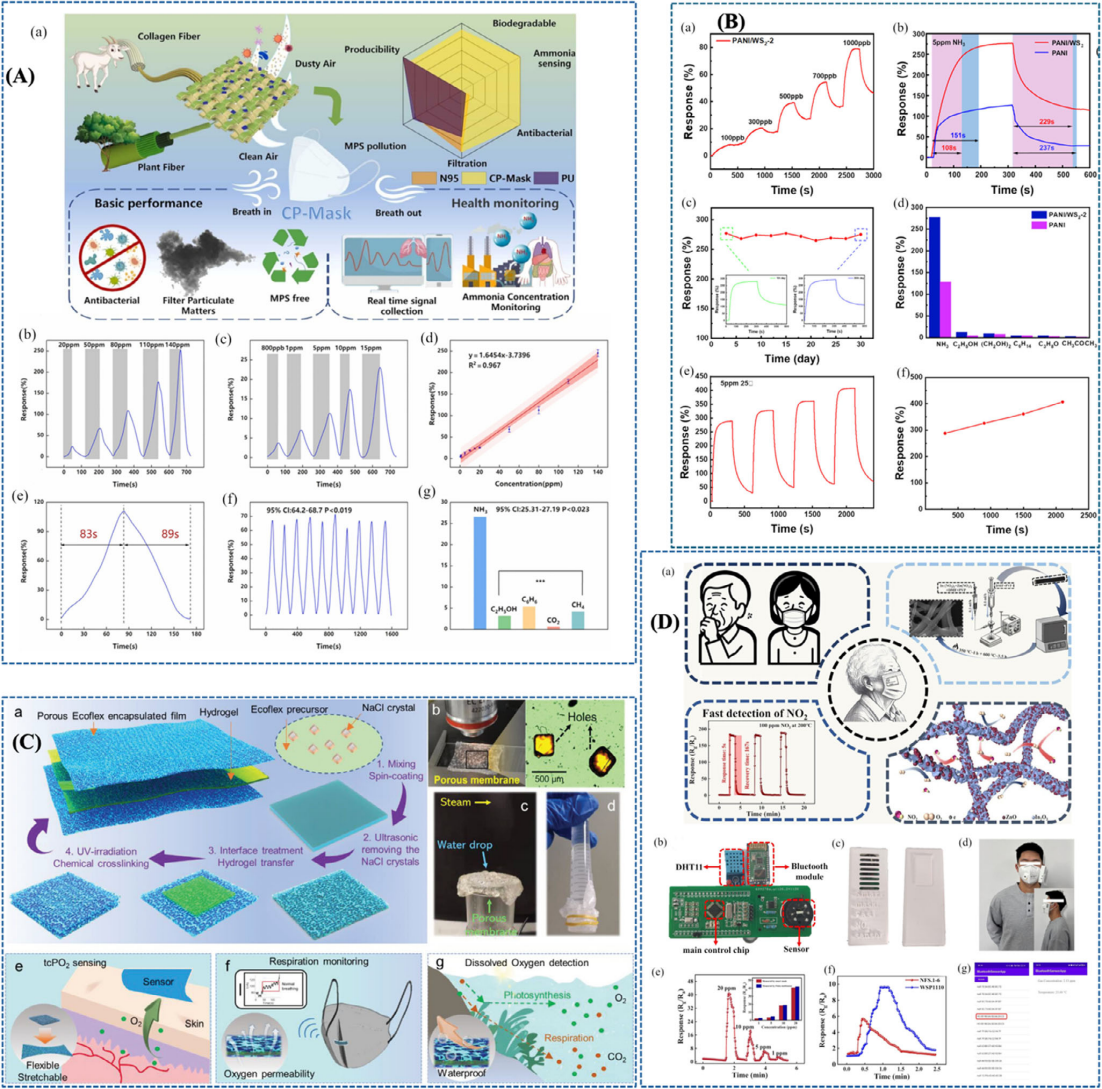
(A) Infographic of chitosan crosslinked animal–collagen plant composite fibers‐based gas sensitive mask with ammonia sensing ability. Reproduced with permission [192]. Copyright 2024, Elsevier B.V. (B) Pictorial outline of in situ‐polymerized PANI/WS2 nanocomposites for highly sensitive flexible ammonia gas sensors and respiration monitoring. Reproduced with permission [193]. Copyright 2024, American Chemical Society. (C) Graphical illustration of humidity‐resistant, sensitive, stretchable PAM/CARR double network hydrogel‐based oxygen sensor for wireless health monitoring. Reproduced with permission [194]. Copyright 2023, Wiley‐VCH GmbH. (D) Pictorial representation of smart mask based on In_2_O_3_@ZnO nanofibers synthesized by coaxial electrospinning for NO_2_ detection. Reproduced with permission [195]. Copyright 2024, Elsevier B.V.

An environmentally friendly ammonia‐sensing mask based on basal dialdehyde–chitosan crosslinked collagen/plant composite fibers (CP‐Mask) has been developed for wearable environmental and health monitoring [192]. The sensing mechanism relies on the adsorption of ammonia molecules on the MoS_2_‐modified fiber surface, which induces resistance changes in the composite structure. The device responds to ammonia concentrations ranging from 800 ppb to 140 ppm, covering both low concentrations in human breath and higher levels in polluted environments. Ammonia is an important indicator of physiological and environmental conditions, as elevated levels in exhaled breath can be associated with metabolic disorders such as kidney or liver dysfunction, while higher ambient concentrations are linked to industrial emissions and air pollution. The CP‐Mask provides a multifunctional wearable solution for protection against air pollution, infectious diseases, and microplastics, supporting the development of sustainable protective devices (Figure 14A).

In another study for ammonia detection, Feng et al. [193] developed a flexible gas sensor using in situ polymerized polyaniline (PANI)/WS_2_ nanocomposites deposited on flexible interdigital electrodes. The sensor operates at room temperature, overcoming the limitation of many metal oxide sensors that require high temperatures, and relies on the protonation‐dedoping mechanism of PANI. When NH_3_ molecules interact with the protonated PANI, the alkaline ammonia neutralizes the protons, reducing PANI's conductivity; upon desorption, PANI is re‐protonated, restoring its conductivity. This effect is further enhanced by the p–n heterojunction formed between p‐type PANI and n‐type WS_2_, which creates a depletion layer and built‐in electric field that amplifies resistance changes upon NH_3_ adsorption. The PANI/WS_2_ sensor achieves a detection limit as low as 100 ppb and demonstrates good selectivity, repeatability, and stability under bending, enabling applications in real‐time respiration monitoring, wearable ammonia detection, and environmental air quality monitoring (Figure 14B).

Wu et al. [194] developed a porous, elastomer‐encapsulated hydrogel‐based oxygen sensor using a polyacrylamide (PAM) and carrageenan (CARR) hydrogel electrolyte, combined with an oxygen‐permeable Ecoflex encapsulation film. The sensor offers a high sensitivity of 0.335%/ppm, a low detection limit in the ppm range, and a wide detection range from 5 ppm to 90% O_2_. It shows response and recovery times of 283.3 s and 269.6 s, respectively. At 4% O_2_, the sensor maintains strong responses across different humidity levels, 1945% at 45% RH, 2016% at 60% RH, and 1965% at 78% RH, demonstrating stable performance in varying humidity. It also remains reliable across temperatures from −10°C to 50°C and can stretch up to 100% strain. When placed on a mask, the sensor detects lower O_2_ during exhalation and higher levels during inhalation, with signal fluctuations matching the breathing rate. It performs consistently across different breathing patterns, including normal, paused, and deep breaths. These results highlight its strong potential for respiration monitoring and health‐related applications (Figure 14C).

In the work by Kang et al. [195], a NO_2_ sensor was developed using coaxially electrospun In_2_O_3_@ZnO porous nanofibers for integration into a smart mask. The nanofiber structure enhances gas adsorption, and the n–n junction between In_2_O_3_ and ZnO improves electron transport, giving a measurable response to NO_2_. While the sensor demonstrates real‐time monitoring capability, its response speed (∼171 s to 100 ppm NO_2_) is relatively slow compared to other wearable gas sensors. The device operates at elevated temperatures (∼200 °C), which limits its direct skin‐contact applications. Nonetheless, it is selective for NO_2_ and highlights the potential of metal oxide nanofibers for personal environmental monitoring in polluted or industrial settings (Figure 14D).

### 4.5 Strain Sensor

Strain sensors detect deformation or displacement in materials by measuring changes in length, pressure, or stress. They play a vital role in monitoring structural integrity, motion, and physiological signals such as breathing or muscle activity. Flexible strain sensors have a wide range of applications in everyday life, including respiratory monitoring through integration into face masks [201, 202]. The sensing behavior of strain sensors varies based on the structural design at the micro or nanoscale, the choice of material, and the fabrication technique. While conventional strain gauges detect deformation mainly through changes in the inherent piezoresistive properties of the material and geometry, flexible and stretchable strain sensors rely on different working principles. These include the breaking and reformation of conductive networks, the development of microcracks in thin films, and the tunneling of electrons between closely spaced conductive elements, all of which enable them to maintain performance under large deformations [127].

**FIGURE 15:**
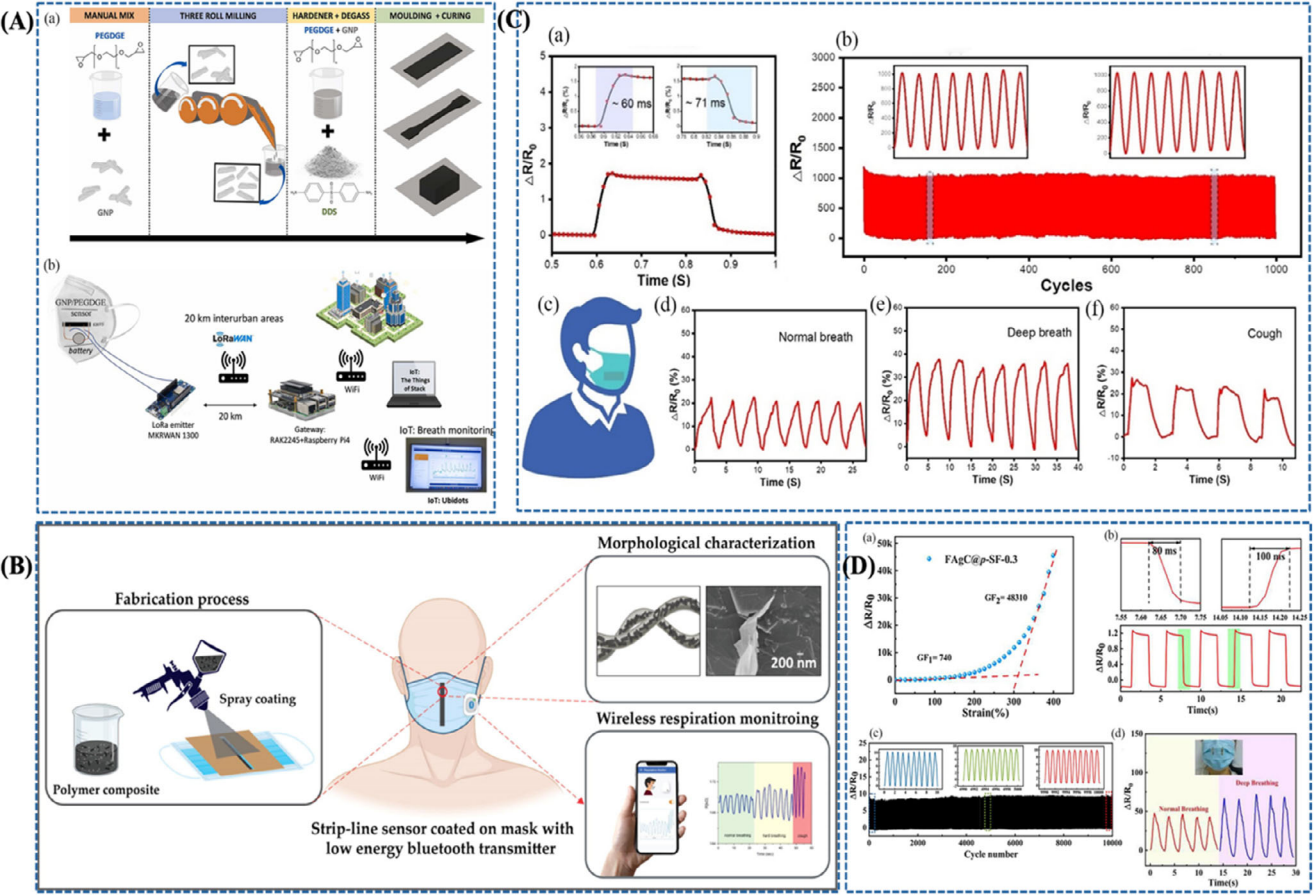
(A) Illustration of GNP/PEG diglycidyl ether based ultrasensitive flexible strain sensors for breathing monitoring. Reproduced with permission [203]. Copyright 2023, Elsevier B.V. (B) Graphical abstract of wearable graphene‐based smart face mask for real‐time human respiration monitoring. Reproduced with permission [107]. Copyright 2024, Elsevier B.V. (C) Schematic diagram of supersensitive wearable sensor constructed with PDMS porous foam and multi‐integrated conductive pathways structure for monitoring different breath patterns. Reproduced with permission [204]. Copyright 2022, Elsevier Ltd and Techna Group S.r.l. (D) Graphical mapping of FAgC@p‐SF waterproof conductive fiber strain sensor for respiration monitoring. Reproduced with permission [205]. Copyright 2022, Elsevier B.V.

In study developed by del Bosque et al. [203], graphene nanoplatelets (GNPs) dispersed in a soft polymer network (PEGDGE) create a percolative piezoresistive path, chosen for graphene's high conductivity and mechanical compliance; this yields very high gauge factors (> 50–100 at low strain and > 1000 at higher strain), enabling subtle breathing strain detection when the sensor is attached to a conventional mask and interfaced with IoT systems, though long‐term drift and environmental stability under repeated breathing were noted as practical concerns (Figure 15A).

The wearable graphene‐based smart face mask for real‐time human respiration monitoring uses a graphene nanoplatelet/polycaprolactone (PCL) composite coating on surgical masks [107], where the conductive network transforms airflow‐induced mask deformation into resistance changes with rapid response (∼42 ms) and durable cycling. Multiple graphene‐based piezoresistive strip‐line sensors are sprayed onto the external surface of a commercial surgical mask, and the authors explicitly place these in three different zones, left, right, and central positions, to determine optimal breathing signal response (Figure 15B).

A supersensitive wearable sensor comprising CNT and MXene integrated on a porous PDMS foam [204] exploits hierarchical conductive pathways and graded fracture under strain to achieve extremely high sensitivity and robustness to large deformations, yet its bulkier porous structure and lack of explicit on‐mask respiratory testing highlight integration challenges (Figure 15C).

Finally, a waterproof conductive fiber with microcracked synergistic conductive layer, developed by Yung et al. [205], demonstrates a tunable wearable strain response through engineered microcracks in the conductive layer, providing stable tuning of sensitivity across strains applicable to motion and physiological signals, but translating such fiber sensors to direct mask respiration monitoring requires careful placement and calibration to isolate breath versus facial movement signals (Figure 15D). Resistive‐type strain sensors offer strong potential for monitoring subtle physiological movements, such as respiration, due to their high sensitivity at low strain levels. Across recent studies, material choice directly influences sensor performance, with nanocarbon‐rich composites providing high gauge factors and tunable fiber or porous designs enabling flexibility and wide dynamic range. Nonetheless, several practical limitations remain, including signal hysteresis, response stability issues such as overshoot and decay, environmental effects (humidity, temperature), mechanical fatigue from continuous respiration cycles, and the need for durable yet flexible encapsulation. Additionally, considerations like user comfort and consistent sensor–mask coupling are critical for reliable wearable integration, emphasizing that careful materials selection and structural design are essential for translating these sensors into practical respiration‐monitoring devices.

More examples are listed in Table 7, sorted by their GF.

**TABLE 7:** Summary of strain sensors sorted by their GF.

Material	GF	Working range (%)	Limit of detection	Response/recovery time (ms)	Year
AgNPs/CNTs, PFDT [205]	48,310	0–400	N/R	80/100	2023
CNT/MXene/PDMS [204]	247 5227 26,438	0–50 50–100 100	N/R	60/70	2023
GNPs/epoxy resin/PEGDGE [203]	50–100 1000–2500	1–2 10	N/R	300/400–600	2023
rGO/pDNPs/PDMS [206]	1523	0–45	1%	47/109	2021
(CCAP)/PDMS [207]	68 248	0–100 100–120	0.01%	50	2019
AgNPs/PU [208]	181.1	100 12.2–12.4	N/R	565.25	2023
AgMFs/PANI/PU [209]	173.66	0–8.75	N/R	10	2019
GNPs/PCL [107]	4.2	0–0.9	N/R	42	2023
GNP/CB/CNT/PU [210]	2.14	0–100	0.50%	65	2018

### 4.6 Temperature Sensor

Continuous temperature monitoring is an important component of health assessment, and recent advances in flexible temperature sensors have expanded their use in medical monitoring, wearable electronics, robotics, and other real‐time applications [211, 212, 213, 214, 215, 216] In smart masks, although temperature sensing has been less extensively studied than pressure‐based respiratory monitoring, it offers a direct means of tracking respiration by detecting the thermal fluctuations of inhaled and exhaled air within the mask microenvironment. Among the temperature sensors explored for this purpose, thermocouples and thermistors are the most common, with thermocouples being particularly attractive because of their simple structure, wide operating range, and self‐generated electrical output. Thermocouple sensing is governed by the Seebeck effect, in which a temperature difference between the sensing junction and the reference end of two dissimilar conductors generates a thermoelectric voltage, expressed as *V*  = (*S*_A_ − *S*_B_) (*T*_h_ − *T*_c_), where *S*_A_ and *S*_B_ are the Seebeck coefficients of the two materials and *T*_h_ − *T*_c_ is the temperature difference between the hot and cold junctions, enabling direct conversion of breath‐induced thermal variations into electrical signals for real‐time respiratory monitoring [217].

Temperature sensing in mask‐based respiratory systems can also be achieved using thermistors, which operate through the temperature dependence of electrical resistance in semiconducting materials. In most smart‐mask applications, negative temperature coefficient thermistors are employed, where the resistance decreases with increasing temperature according to [218]

$$R\;\left(T\right)=R_{0}\;\exp\left[B\left(\frac{1}{T}\frac{1}{T_{0}}\right)\right]\;$$

where *R*_0_ is the resistance at the reference temperature *T*_0_, *T* is the absolute temperature, and *B* is a material‐specific constant. In mask‐integrated respiratory monitoring, this mechanism enables the temperature variation between inhaled and exhaled air to be translated into measurable resistance changes, allowing continuous and real‐time breath‐temperature detection.

**FIGURE 16:**
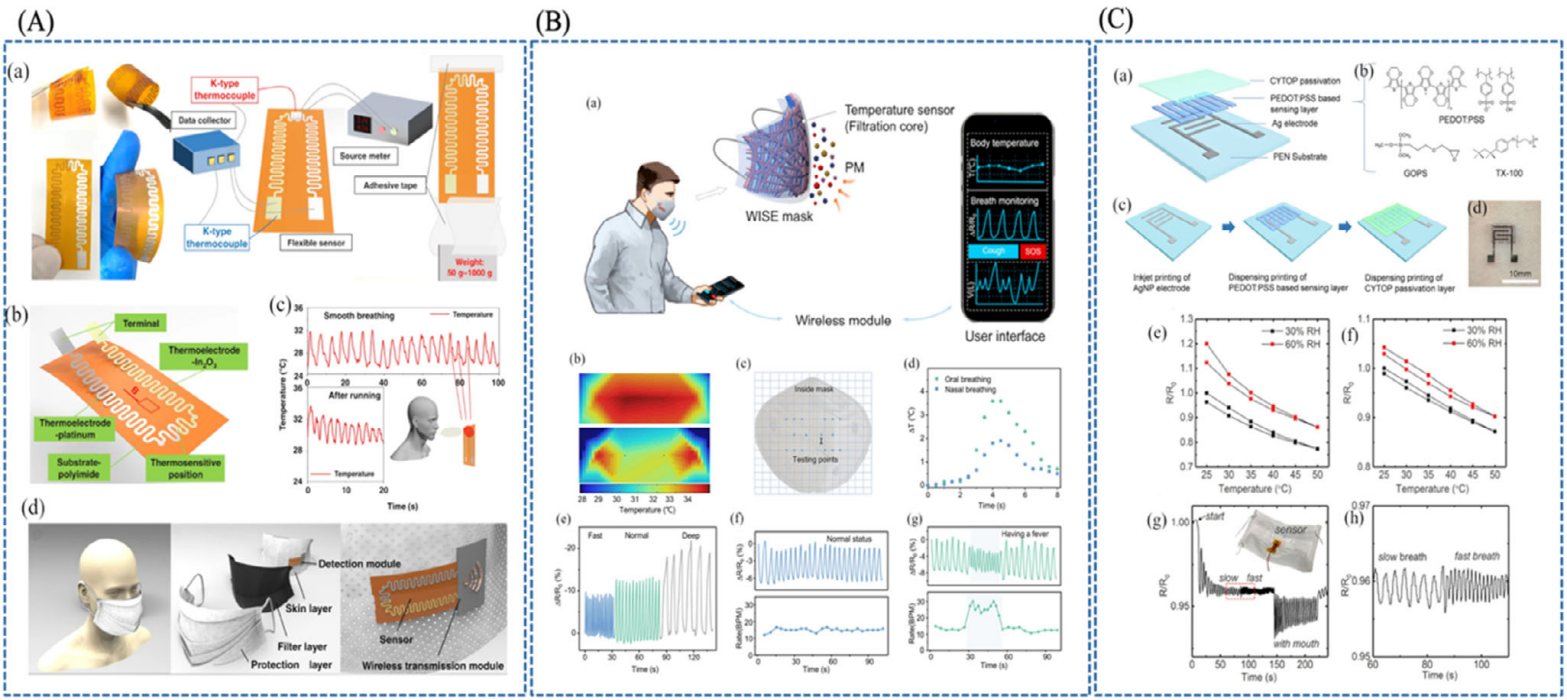
(A) Schematic diagram of micro 3D flexible thin‐film thermocouples based on platinum and indium oxide electrodes. Reproduced with permission [219]. Copyright 2021, Springer Nature. (B) Illustration of wireless all‐in‐one sensory face mask made of ultrasensitive fibrous temperature sensors based on PP/PAN‐PA bilayered nonwoven. Reproduced with permission [220]. Copyright 2023, American Chemical Society. (C) Infographic of fully printed PEDOT:PSS‐based temperature sensor with high humidity stability for wireless healthcare monitoring. Reproduced with permission [215]. Copyright 2020, Springer Nature.

Lui et al. [219] investigated a flexible thin‐film thermocouple temperature sensor for respiratory monitoring, employing polyimide as the deformable substrate and Pt as the thermoelectrode pair in a curvilinear configuration to improve mechanical compliance under bending. In this design, the flexible substrate and curved electrode layout help preserve signal stability during repeated deformation. As a result, the device achieved a maximum sensitivity of 76.5 µV/K at a temperature difference of 100°C, while the sensitivity further increased from 57.1 to 83.0 µV/K after 1000 deformation cycles, indicating good mechanical adaptability and stable thermal response. The sensor also showed reliable temperature retention during continuous operation for up to 3 h and, when integrated into masks with and without breathing valves, successfully captured exhaled‐breath temperature changes and distinguished smooth breathing from postactivity breathing (Figure 16A).

Wang et al. [215] reported a printed thermistor based on crosslinked PEDOT:PSS with (3‐glycidyloxypropyl) trimethoxy silane along with the nonionic surfactant Triton (Triton X‐100), in which the distinctive material design enables a flexible conductive network with improved humidity tolerance, mechanical stability, and device‐to‐device reproducibility. These features are particularly important for mask‐integrated operation, where moisture exposure and repeated deformation can degrade sensor reliability. The sensor delivered a high sensitivity of −0.77% in the physiologically relevant 25–50°C range and was successfully used to monitor breathing when mounted on the inner side of a mask (Figure 16C).

Zhao et al. [220] developed the wireless all‐in‐one sensory face mask (WISE mask) as a fully integrated smart mask rather than a sensor attached to a conventional mask, thereby reducing bulk and improving wearing comfort while enabling multifunctional respiratory analysis. The temperature‐sensing element was based on a PP/PAN‐PA material system, in which phytic acid served as a proton‐transporting component that enhanced the thermoresistive response and enabled a high thermal coefficient of resistance of %, a temperature resolution of 0.2°C, and rapid signal generation suitable for tracking the subtle thermal fluctuations associated with inhalation and exhalation. Beyond basic respiration monitoring, the main innovation of this work lies in extending mask‐based temperature sensing to flu‐symptom analysis: the breathing signals were converted into lung‐volume‐related curves from which and FVC could be estimated, allowing the mask to move from simple breath detection toward functional respiratory assessment. This study therefore distinguishes itself from more conventional mask‐mounted temperature sensors by integrating the sensing function directly into the mask architecture and by linking thermistor‐based breath monitoring to clinically relevant pulmonary screening concepts, which is consistent with the general use of thermally induced resistance changes in thermistor sensing (Figure 16B).

## 5 Self‐Powered Wearable Sensors

Self‐powered smart masks have emerged as a promising platform for breath‐based healthcare monitoring because conventional sensors often rely on external power supplies, can be bulky, and may show insufficient sensitivity, selectivity, or real‐time responsiveness under physiological and dynamically changing conditions. These limitations are particularly problematic for mask‐integrated systems intended for continuous wear, where low power consumption, miniaturization, flexibility, and user comfort are essential for practical deployment. In this context, self‐powered sensing strategies provide an attractive alternative by coupling sensing units with energy harvesters that convert ambient mechanical energy, such as breathing, facial motion, or other human movements, into usable electrical output. Among the available approaches, triboelectric nanogenerators have attracted special attention because they enable battery‐free or battery‐assisted operation while supporting lightweight, wearable, and portable device architectures for real‐time health monitoring [221, 222, 223, 224]. As a result, self‐powered smart masks are increasingly viewed as a key direction for next‐generation personalized healthcare systems, offering a route toward autonomous, low‐maintenance, and continuously operating platforms for exhaled‐breath analysis.

### 5.1 Triboelectric Effect

**FIGURE 17:**
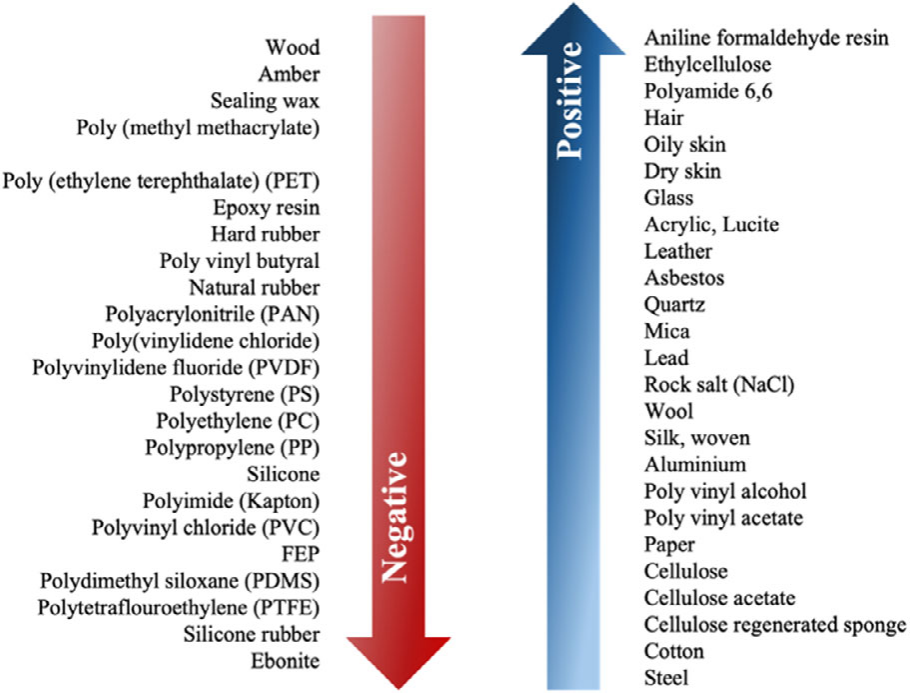
Triboelectric series of selected materials. Reproduced with permission [238]. Copyright 2020, Wiley‐VCH GmbH.

The triboelectric effect is a form of contact electrification in which electrical charges accumulate on the surfaces of two materials after they are brought into contact and then separated. This charge transfer occurs due to differences in the materials’ electron affinities, based on its position in triboelectric series (Figure 17), and is influenced by both intrinsic factors, such as dielectric properties and surface energy, and extrinsic conditions like contact force, roughness, and environmental humidity [225]. Upon separation, one material gains electrons and becomes negatively charged, while the other loses electrons and becomes positively charged. The direction and magnitude of charge transfer depend on the relative positions of the materials in the triboelectric series; for instance, polytetrafluoroethylene (PTFE) tends to attract electrons, whereas materials like nylon are more likely to lose them [226]. Although the exact microscopic mechanism is still under investigation, this phenomenon underpins a wide range of applications, particularly in energy harvesting and sensing.

#### 5.1.1 Triboelectric Nanogenerator

A TENG is a device that exploits the triboelectric effect in conjunction with electrostatic induction to convert ambient mechanical energy into usable electrical power. In a typical TENG, two triboelectrically dissimilar materials are repeatedly brought into contact and then separated through mechanical motion such as tapping, sliding, or bending. This mechanical interaction generates surface charges via the triboelectric effect. Once the materials are separated, the resulting electric field induces a potential difference between the associated electrodes, driving electrons through an external circuit. As the cycle continues, alternating current is produced. TENGs are highly versatile and can operate in four principal modes, vertical contact–separation, lateral sliding, single‐electrode, and freestanding triboelectric‐layer configurations [227], allowing for adaptation to diverse mechanical stimuli. In the vertical contact–separation mode, two surfaces are pressed together and then pulled apart vertically, generating current through repeated contact and separation. The lateral sliding mode involves tangential motion between surfaces, producing a dynamic charge distribution as the materials slide relative to each other. The single‐electrode mode uses only one active electrode and relies on interaction with external surfaces or bodies, making it especially suitable for wearable or touch‐based applications. Lastly, the freestanding triboelectric‐layer mode employs a mobile charged layer moving between two fixed electrodes, enabling contact‐free operation and higher durability. Their performance depends on material properties, surface microstructure, motion dynamics, and environmental stability. Recent developments have focused on improving output efficiency and durability through nanostructured surfaces, advanced polymer composites (e.g., PDMS, Kapton), and effective encapsulation. Due to their lightweight construction, flexibility, and low fabrication cost, TENGs have emerged as a promising technology for powering wearable devices, health‐monitoring systems, and distributed sensor networks in self‐ sustained electronics.

#### 5.1.2 Smart Mask Application

Several recent solutions have proposed innovative facemask‐integrated systems based on TENGs, each offering unique functionalities, performance advantages, and targeted applications.

**FIGURE 18:**
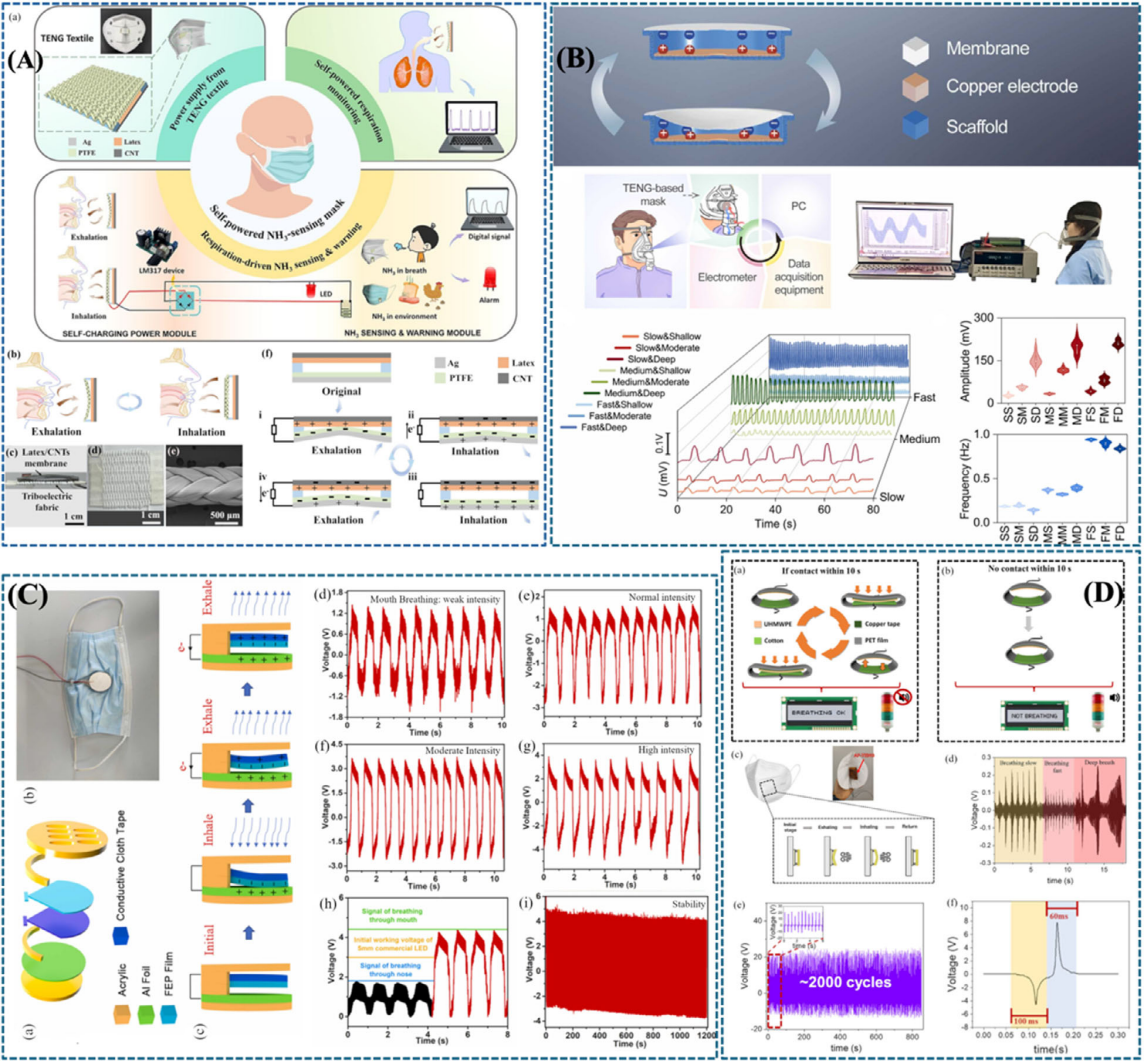
(A) Schematic abstract of a self‐powered smart mask by integrating fabric‐based TENG and Ti_3_C_2_T*_x_*/PANI‐based NH_3_ sensing module. Reproduced with permission [228]. Copyright 2025, Elsevier B.V. (B) Infographic of a self‐sensing respiratory ventilator mask consisting of a single‐electrode‐mode TENG valve core. Reproduced with permission [229]. Copyright 2024, Elsevier B.V. (C) Illustration of respiratory sensing TENG intelligent face mask based on Al foil/FEP film with apnea alarm system. Reproduced with permission [230]. Copyright 2021, Elsevier B.V. (D) Graphical abstract of all‐fabric TENG smart mask using UHMWPE and cotton fabric as triboelectric layers for remote long rate breathing monitoring and apnea alarm. Reproduced with permission [55]. Copyright 2023, American Chemical Society.

Huang et al. developed a respiration‐driven self‐powered smart mask centered on a textile TENG, in which triboelectric yarns and a latex/CNT membrane operate in contact–separation mode to convert breathing‐induced mechanical motion into electrical signals for autonomous respiration monitoring. This triboelectric architecture acts as both the energy‐harvesting unit and the sensing basis for distinguishing breathing states, while an integrated MXene‐based module enables ammonia detection. The device also showed strong NH_3_‐sensing performance, with a 550% response at 100 ppm, a detection limit of 50 ppb, high selectivity, and good long‐term stability (Figure 18A) [228].

In contrast, Hu et al. developed a triboelectrically self‐sensing ventilator mask that places greater emphasis on the TENG mechanism itself by designing a single‐electrode ventilator mask around a PlasCLEAR resin scaffold, a deformable PDMS membrane, and a Cu electrode. This single‐electrode triboelectric valve system converts breathing airflow into electrical outputs that track both respiratory rhythm and depth, thereby enabling real‐time monitoring of diverse breathing states while also supporting interactive functions such as voice‐based control (Figure 18B) [229].

Lu et al. reported a more straightforward triboelectric respiratory‐monitoring design of smart face mask that integrates a respiratory triboelectric nanogenerator (RS‐TENG) as a lightweight and wearable sensing unit. The device works by converting the airflow and deformation during breathing into electrical signals through the triboelectric effect. The sensor produces output signals up to about 8 V under different respiratory conditions and is able to differentiate between nose and mouth breathing, highlighting its sensitivity to subtle changes in respiration. In addition, the RS‐TENG functions without an external power supply and shows stable operation under different humidity levels. The mask design is relatively simple and low‐cost, making it a practical example of a self‐powered respiratory monitoring system with potential for health and daily‐life applications (Figure 18C) [230].

Alternatively, Vázquez‐López et al. developed fabric‐based TENG, desirable to be put inside face mask, using cotton fabric and ultrahigh molecular weight polyethylene (UHMWPE), acting as positive and negative triboelectric layers, respectively. This contact mode TENG is employed for respiration monitoring to analyze breath rhythm. This smart mask clearly demonstrates a strong link between generated voltage and breathing rate. It distinguishes fast, slow, and deep breathing by changes in pulse shape and frequency, highlighting the TENGs’ potential for advanced respiratory monitoring. Besides tracking breathing patterns, the smart mask also works as an alarm system. It can detect the absence of breathing and trigger a sound or display warning, offering a simple yet effective setup like hospital monitoring system (Figure 18C) [55].

These innovations showcase the remarkable versatility of TENG‐based smart facemasks and their potential for personalized, self‐powered healthcare and assistive technologies, more examples are discussed in Table 8.

**TABLE 8:** Summary of triboelectric respiratory sensors.

Materials used	Performances/detection limit	Primary application	TENG mode
MXene (Ti_3_C_2_T*_x_*) nanosheets, textile‐based TENG [228]	NH_3_ sensing: 550% at 100 ppm, 50 ppb detection limit, excellent long‐term stability, high selectivity	Real‐time respiration monitoring and NH_3_ detection	Vertical contact–separation
PlasCLEAR resin (3D‐printed scaffold), PDMS membrane, copper electrode [229]	Real‐time airflow control, deep‐learning voice recognition (87% accuracy), ergonomic adaptability	Intelligent closed‐loop respiratory therapy and emergency support	Single‐electrode
Ce‐doped ZnO, polyaniline (PANI) [231]	NH_3_ sensing: high sensitivity, excellent linearity (1–25 ppm), robust in humid conditions	Health monitoring (altered exhaled ammonia) and respiration sensing	Vertical contact–separation
FEP film and Al foil [230]	Generates up to 8 V and 0.8 µA through normal breathing, integrated apnea alarm, immune to humidity degradation	Sleep apnea and respiratory distress monitoring	Vertical contact–separation
Polyethylene (UHMWPE), cotton fabric [55]	Remote breathing monitoring, apnea alarm via Wi‐Fi/LoRa (up to 20 km), eco‐friendly	Remote breathing monitoring and apnea alarm	Vertical contact–separation
Polypropylene (PP), polyurethane (PU) [232]	Enhanced air filtration efficiency (up to 99% for 10 nm–100 µm particles), self‐powered	Enhanced air filtration	Vertical contact–separation
PDMS (polydimethylsiloxane), conductive fabric, silver nanowires, Ecoflex [233]	High sensitivity (detects subtle respiratory patterns), accuracy > 90% in sleep stage classification	Smart sleep monitoring systems (detecting sleep apnea, snoring, and sleep stages)	Single‐electrode
PVDF (polyvinylidene fluoride) nanofibers, conductive fabric, PET (polyethylene terephthalate) substrate [234]	Detects multiple respiratory indices (breathing rate, depth, patterns), real‐time monitoring with high sensitivity	Respiratory monitoring	Contact–separation
PTFE (polytetrafluoroethylene), nylon fabric, conductive textile, Cu electrodes [235]	High sensitivity (detects respiration rate, intensity, and patterns), real‐time monitoring, self‐powered operation	Wearable respiration monitoring (sleep apnea, asthma, COPD monitoring)	Contact–separation
PDMS, Ecoflex, graphene‐coated textiles, silver nanowire electrodes [236]	High sensitivity (detects respiratory rate, tidal volume, abnormal breathing patterns), low detection limit, long‐term stability	Self‐powered wearable respiratory monitoring (COPD, asthma, sleep disorders, fitness tracking)	Hybrid (contact–separation + sliding TENG)
Ag micromesh films, electrospun PVDF nanofiber membrane (PVDF NM), polyamide 6 nanofiber membrane [237]	‐ Electrothermal sterilization: heats to 60°C in 30 s at 3 V, 95.58% antibacterial efficiency ‐ Respiratory monitoring: detects tachypnea/apnea in real‐time with self‐powered alarm system	Reusable medical masks with dual‐function: 1. Self‐sterilization 2. Real‐time respiratory monitoring	Single‐electrode

## 6 Challenges and Future Prospects

Despite substantial progress, practical deployment still hinges on resolving persistent technical constraints in sensitivity, selectivity, and response time under real breathing conditions.

### 6.1 Cross‐Sensitivity

Cross‐sensitivity remains a major barrier for gas and humidity sensing in smart masks because real‐world operation introduces multiple overlapping interference channels. For gas sensors, fluctuations in humidity and temperature, together with coexisting VOCs in exhaled breath and the surrounding environment, can shift baselines, reduce biomarker specificity, and trigger false alarms. In addition, many mask‐integrated sensors rely on electrical readouts such as changes in resistance or capacitance, so routine user motion (talking, jaw movement, strap tension changes, mask flutter during breathing) can introduce unintended piezoresistive or contact‐related artifacts that can be misinterpreted as humidity or gas responses. Future designs can mitigate these effects by combining more selective sensing layers (e.g., functional coatings, catalytic or sorbent filters, and metal–organic framework (MOF)/MXene‐enabled interfaces) with system‐level compensation, such as dedicated temperature/humidity channels for correction, differential or reference sensing to subtract mechanical/common‐mode drift, and packaging that mechanically decouples the sensing element from deformation, supported by lightweight sensor‐fusion algorithms that separate rapid motion artifacts from slower adsorption/desorption dynamics.

### 6.2 Long‐Term Stability

Long‐term stability is equally critical for smart masks because performance must remain dependable over weeks to months of real wear, not only during short laboratory tests. In practice, sensors can drift due to aging of the active material and repeated humidity/temperature cycling, while condensation and fouling from saliva aerosols, ambient particulates, skin oils, and cosmetics can block active sites or create parasitic conduction paths that reduce sensitivity and slow response/recovery. Mechanical fatigue further accelerates degradation: daily don/doff cycles, folding or extrusion during storage, and continuous micro‐bending from breathing and speaking can crack films, fracture electrodes, or change contact resistance, leading to intermittent signals and reduced reliability. These durability issues are closely tied to comfort and safety, since accurate detection often requires strong coupling to respiratory airflow; however, integrating sensors can alter the mask's internal structure, worsen fit, increase breathing resistance, and cause discomfort or breathlessness, while prolonged contact may trigger skin reactions such as itching, redness, or swelling.

Mitigating these challenges will require packaging and materials strategies that protect electronics without blocking analyte transport, such as breathable encapsulation layers, hydrophobic/antifouling barriers, and moisture‐managed airflow pathways, alongside mechanically tolerant designs (strain relief, flexible interconnects, and placing rigid components away from high‐deformation regions). Because degradation is often gradual and hard to notice, practical systems should also incorporate periodic or self‐calibration, reference channels, and drift‐aware algorithms to detect baseline shifts and compensate them before they compromise interpretation. Finally, standardized validation protocols should explicitly include realistic wear cycles, temperature/humidity swings, repeated bending/folding, and cleaning or sterilization exposures, while also evaluating breathing resistance, skin compatibility, and electrical safety under worst‐case damage scenarios (e.g., cracked traces or exposed conductors).

### 6.3 Wearability and Safety

For smart masks to move from prototypes to routine clinical, occupational, or consumer use, design priorities must shift from “can it sense?” to “will people wear it, trust it, and afford it?”. Accurate biomarker detection often requires strong coupling between the sensor and the user's respiratory airflow, yet integrating biosensors can change the mask's internal structure and compromise its original fit. This can increase breathing resistance and lead to discomfort or even breathlessness (similar to breathing in a confined space), while prolonged wear may provoke adverse skin reactions such as itching, redness, and swelling due to direct or indirect contact between sensor components and the epidermis. Wearability factors include pressure on the nose and ears, thermal discomfort, moisture buildup, and speech or communication interference. These factors directly affect compliance and data quality. Leakage and inconsistent fit can alter airflow at the sensor interface and distort the measured signals.

Safety is equally central because the inner mask layer contacts the nose and mouth, among the body's most vulnerable regions. If sensors or circuits degrade during use through daily folding and extrusion, repeated don and doff cycles, or bending fatigue, electrodes and interconnects can crack or become exposed. This can cause intermittent performance or abnormal heating. In worst cases, current leakage may occur and potentially harm the user. These risks argue for mechanically tolerant layouts, electrical insulation and sealed contacts, and designs that keep rigid or failure‐prone electronics away from high‐deformation zones. From a hygiene and sustainability perspective, a modular approach is often most practical: an externally mounted or clip‐on reusable sensor pod (with sealed electrical interfaces) paired with replaceable low‐cost filter layers reduces waste, simplifies cleaning, and lowers cost, while still maintaining sensing performance. Antimicrobial/antiviral coatings or inherently biocidal substrates may support safer extended wear, but they must be selected and validated carefully to avoid sensor passivation, signal suppression, or accelerated material aging.

### 6.4 Power, Integration, and Cost‐Effective Manufacturing

Equally important for translation (as discussed above for wearability and long‐term reliability) are power management and end‐to‐end system engineering. Battery‐powered smart masks must trade off sampling rate, wireless transmission, and on‐device computation, because aggressive sensing and continuous streaming can quickly drain small form‐factor batteries and increase user burden. Practical routes to extend runtime include duty‐cycling, event‐triggered acquisition (e.g., higher sampling only during cough‐like events), low‐power BLE communication, and on‐device/edge inference to reduce raw‐data transmission while also improving privacy. Self‐powered options such as triboelectric nanogenerators are promising for maintenance‐free operation, but they require stable, repeatable output under variable breathing and motion, and they must interface reliably with power conditioning and signal‐processing electronics.

At the system‐integration level, multisensor platforms can introduce electrical and mechanical cross‐talk, added packaging complexity, and unit‐to‐unit variability. These issues often become more visible when moving toward scalable fabrication routes (printing, roll‐to‐roll, lamination), where small variations in layer thickness, alignment, and contact resistance can create large performance spread and complicate regulatory expectations for repeatability and safety. Cost‐effective commercialization will therefore depend on reducing bill‐of‐materials, simplifying assembly and interconnects, enabling automated calibration/testing, and using interoperable data formats for deployment across devices and clinical workflows. In parallel, secure handling of sensitive respiratory data remains essential, favoring encryption, minimal necessary data capture, and on‐device processing when feasible with transparent user consent.

### 6.5 AI and Machine Learning

AI and machine learning will become central to smart masks by turning complex, multisensor signals into reliable, actionable outputs. Multisensor fusion can cross‐validate humidity, flow/pressure, strain, temperature, and gas measurements to reduce false alarms, while artifact‐rejection models can distinguish true respiratory events from motion‐related disturbances caused by talking, walking, or mask readjustment. ML can also support long‐term use through drift compensation and personalized baselines, maintaining performance as sensors age and conditions change, and enabling earlier anomaly detection than fixed‐threshold approaches. To translate these benefits into real‐world tools, models should be optimized for low‐power edge inference to limit continuous data streaming, strengthen privacy, and reduce energy consumption, and they must be trained/validated on diverse, realistic datasets to ensure robust, clinically meaningful performance at scale.

### 6.6 Material Innovation

Looking ahead, materials innovation should be framed less as “new nanomaterials” and more as application‐driven material stacks that directly address the dominant failure modes in smart masks (cross‐sensitivity, drift, moisture, and low‐power operation). For example, MXenes are attractive because their high conductivity and surface terminations enable room‐temperature, low‐power chemiresistive sensing, and recent work continues to position them as strong candidates for breath‐biomarker monitoring under wearable constraints. In parallel, MOFs offer a fundamentally different advantage: their tunable pore size/chemistry and adsorption affinity make them useful as selective recognition layers or preconcentrators that can enrich trace VOCs and improve selectivity, including for clinically relevant targets such as ammonia. A practical future direction is therefore hybrid architectures (e.g., MXene transducers + MOF selective layers), where the MOF performs molecular sieving/enrichment while the conductive layer provides stable electrical transduction, explicitly targeting cross‐sensitivity reduction without increasing heater power.

Sustainability‐focused material choices should also be positioned as an enabling pathway for scalable deployment, especially when combined with the modular “reusable sensor + replaceable filter” concept discussed above. Biodegradable substrates based on cellulose and chitosan are increasingly explored for flexible biomedical devices because they can offer biocompatibility and environmental degradability while still supporting functional performance when engineered appropriately. The near‐term direction is to treat eco‐friendly substrates as the disposable component (filter layer) and reserve advanced materials (MXenes/MOFs/graphene composites) for a reusable sensing module, which reduces material cost, simplifies sterilization, and lowers lifecycle waste without sacrificing sensing capability.

## 7 Conclusions

Smart face masks represent a pivotal advancement in the field of wearable health technologies, shifting from passive protective equipment into intelligent, multifunctional platforms capable of continuously monitoring respiratory activity in real time. Unlike traditional masks, these next‐generation devices are engineered to detect subtle variations in breathing patterns, such as rhythm, frequency, and depth, providing valuable physiological insights that can aid in early disease detection, health monitoring, and even pandemic response strategies. In this review, we presented a comprehensive exploration of materials and sensor types used in the development of smart masks. We began by categorizing sensor‐compatible materials based on their dimensionality (0D, 1D, 2D, and 3D), emphasizing how their unique physicochemical and electrical properties influence performance, sensitivity, and feasibility of integration. Following this, we delved into six primary types of sensors, humidity, pressure, temperature, strain, triboelectric, and gas sensors, detailing their working principles and highlighting notable advancements. These sensors serve distinct yet complementary roles in capturing respiratory signals, and understanding their strengths and limitations is essential for tailoring them to specific applications. Table 9 summarizes their comparative performance, offering a valuable reference for researchers selecting optimal sensor configurations.

**TABLE 9:** Summary of sensor types used in smart masks with key parameters, advantages, and challenges.

Stimuli	Device types	Notable parameter	Advantages	Challenges
Humidity	Impedance	Sensitivity	Noninvasive and real‐time tracking High sensitivity to moisture changes Lightweight and wearable integration	Temperature–humidity trade‐off Instability at high RH Slow response/recovery time
	Capacitive			
Pressure	Piezoresistive	Response recovery time Detection range	High sensitivity Durability Quick response time Multimodal sensing Flexibility and wearability	Signal decoding complexity Drift and hysteresis Mechanical wear
	Capacitive			
	Piezoelectric			
Strain	—	Gauge factor	Flexible Lightweight Fast response Pattern recognition	Nonlinearity of response Sensitive to humidity and temperature Cross‐signal interference (talking, jaw movements)
Gas	Semiconductor	Selectivity of gases	Disease monitoring Hazard environment detection Versatility in target gas detection	Selectivity and cross‐sensitivity Low detection limits for trace biomarkers User comfort and biocompatibility
	Chemiresistive			
	Electrochemical			
Temperature	Thermistor	Sensitivity	Good reliability and Temperature retention Flu tracking High‐temperature resolution (thermistor based)	Temperature distribution Complexity in breath Signal strength for nasal respiration
	Thermocouple			
Triboelectric	—	Generated power	Self‐powered/autonomous operation Real‐time respiration monitoring capabilities Detection of abnormal patterns (sleep)	Influence of extrinsic conditions (jaw movements) Bulkiness of the system

## Author Contributions

**Negin Faramarzi**: Led the topic selection and scope definition; designed the structure of the review; coordinated literature collection and organization; contributed to critical analysis and manuscript structuring; drafted major sections of the paper; prepared visualizations; supervised the writing process; and led the final review and editing. **Naseeh Backer Kannanthodi**: Contributed to literature review and critical analysis; drafted general sections of the manuscript, prepared visualizations; and supported final review and editing. **Alice Nicole Casling**: Led the writing of the triboelectric sensor section; literature collection, contributed to literature analysis in that area; and participated in final review and editing. **Babar Ali**: Drafted the pressure sensor section; literature collection and conducted targeted literature investigation for that topic. **Samira Lakouraj Mansouri**: Drafted the gas, strain, and temperature sensor sections, literature collection, and conducted targeted literature investigation for those topics. **Hossein Cheraghi Bidsorkhi, Alessandro Giuseppe D'Aloia, and Maria Sabrina Sarto**: Provided overall supervision, academic guidance, review and editing, and institutional support.

## Conflicts of Interest

The authors declare no conflicts of interest.

## Data Availability

The authors have nothing to report.

## References

[1] F. Q. AL‐Khalidi , R. Saatchi , D. Burke , H. Elphick , and S. Tan , “Respiration Rate Monitoring Methods: A Review,” Pediatric Pulmonology 46 (2011): 523–529, 10.1002/ppul.21416. 21560260

[2] S. Shen , Q. Zhou , G. Chen , et al., “Advances in Wearable Respiration Sensors,” Materials Today 72: 140–162, 10.1016/j.mattod.2023.12.003.

[3] M. Song , Y. Ma , L. Li , et al., “Multiplexed Detection of SARS‐CoV‐2 Based on Upconversion Luminescence Nanoprobe/MXene Biosensing Platform for COVID‐19 Point‐of‐Care Diagnostics,” Materials & Design 223 (2022): 111249, 10.1016/j.matdes.2022.111249. 36248181 PMC9550287

[4] L. Xu , H. Zhai , X. Chen , et al., “Coolmax/Graphene‐Oxide Functionalized Textile Humidity Sensor With Ultrafast Response for human Activities Monitoring,” Chemical Engineering Journal 412 (2021): 128639, 10.1016/j.cej.2021.128639.

[5] G. Wang , Y. Zhang , H. Yang , et al., “Fast‐Response Humidity Sensor Based on Laser Printing for Respiration Monitoring,” RSC Advances 10 (2020): 8910–8916. 35496566 10.1039/c9ra10409g PMC9050045

[6] J. Dai , H. Zhao , X. Lin , et al., “Ultrafast Response Polyelectrolyte Humidity Sensor for Respiration Monitoring,” ACS Applied Materials & Interfaces 11 (2019): 6483–6490, 10.1021/acsami.8b18904. 30672684

[7] M. Liao , H. Liu , X. Wang , et al., “A Technical Review of Face Mask Wearing in Preventing Respiratory COVID‐19 Transmission,” Current Opinion in Colloid & Interface Science 52 (2021): 101417, 10.1016/j.cocis.2021.101417. 33642918 PMC7902177

[8] A. Vaquer , A. Alba‐Patiño , C. Adrover‐Jaume , et al., “Nanoparticle Transfer Biosensors for the Non‐Invasive Detection of SARS‐CoV‐2 Antigens Trapped in Surgical Face Masks,” Sensors and Actuators, B: Chemical 345 (2021): 130347, 10.1016/j.snb.2021.130347. 34188360 PMC8225299

[9] M. Lazaro , A. Lazaro , R. Villarino , and D. Girbau , “Smart Face Mask With an Integrated Heat Flux Sensor for Fast and Remote People's Healthcare Monitoring,” Sensors 21 (2021): 7472, 10.3390/s21227472. 34833547 PMC8623048

[10] T. M. Fagbola , F. I. Fagbola , O. J. Aroba , R. Doshi , K. K. Hiran , and S. C. Thakur , “Smart Face Masks for COVID‐19 Pandemic Management: A Concise Review of Emerging Architectures, Challenges and Future Research Directions,” IEEE Sensors Journal 23 (2023): 877–888, 10.1109/JSEN.2022.3225067.

[11] C. J. Worby and H.‐H. Chang , “Face Mask Use in the General Population and Optimal Resource Allocation during the COVID‐19 Pandemic,” Nature Communications 11 (2020): 4049, 10.1038/s41467-020-17922-x. PMC7426871 32792562

[12] S. Kwon , A. D. Joshi , C.‐H. Lo , et al., “Association of Social Distancing and Face Mask Use with Risk of COVID‐19,” Nature Communications 12 (2021): 3737. 10.1038/s41467-021-24115-7 PMC8213701 34145289

[13] Y. Song , X. Wang , L. Wang , L. Qu , and X. Zhang , “Functionalized Face Masks as Smart Wearable Sensors for Multiple Sensing,” ACS Sensors 9 (2024): 4520–4535. 39297358 10.1021/acssensors.4c01705

[14] M. Adeel , Y. Cotur , A. Naik , L. Gonzalez‐Macia , and F. Güder , “Face Masks as a Platform for Wearable Sensors,” Nature Electronics 5 (2022): 719–720, 10.1038/s41928-022-00871-2.

[15] M. Ma , Y. Shang , H. Shen , W. Li , and Q. Wang , “Highly Transparent Conductive Ionohydrogel for All‐Climate Wireless Human‐Motion Sensor,” Chemical Engineering Journal 420 (2021): 129865, 10.1016/j.cej.2021.129865.

[16] H. Wang , J. Xiang , X. Wen , et al., “Multifunctional Skin‐Inspired Resilient MXene‐Embedded Nanocomposite Hydrogels for Wireless Wearable Electronics,” Composites, Part A: Applied Science and Manufacturing 155 (2022): 106835, 10.1016/j.compositesa.2022.106835.

[17] Y. Sun , S. Wang , X. Du , Z. Du , H. Wang , and X. Cheng , “Skin‐conformal MXene‐Doped Wearable Sensors With Self‐Adhesive, Dual‐Mode Sensing, and High Sensitivity for Human Motions and Wireless Monitoring,” Journal of Materials Chemistry B 9 (2021): 8667–8675, 10.1039/D1TB01769A. 34610630

[18] W. Cao , Y. Luo , Y. Dai , et al., “Piezoresistive Pressure Sensor Based on a Conductive 3D Sponge Network for Motion Sensing and Human–Machine Interface,” ACS Applied Materials & Interfaces 15 (2023): 3131–3140, 10.1021/acsami.2c18203. 36603144

[19] Z. Sun , C. Dong , B. Chen , et al., “Strong, Tough, and Anti‐Swelling Supramolecular Conductive Hydrogels for Amphibious Motion Sensors,” Small 19 (2023): 2303612. 10.1002/smll.202303612 37394709

[20] J. Shin , S. Noh , J. Lee , et al., “Self‐powered Flexible Piezoelectric Motion Sensor With Spatially Aligned InN Nanowires,” Chemical Engineering Journal 486 (2024): 150205, 10.1016/j.cej.2024.150205.

[21] S. Shen , X. Xiao , X. Xiao , and J. Chen , “Wearable Triboelectric Nanogenerators for Heart Rate Monitoring,” Chemical Communications 57 (2021): 5871–5879, 10.1039/D1CC02091A. 34085071

[22] X. Cui , C. Zhang , W. Liu , et al., “Pulse Sensor Based on Single‐electrode Triboelectric Nanogenerator,” Sensors and Actuators, A: Physical 280 (2018): 326–331, 10.1016/j.sna.2018.07.051.

[23] X. Jin , L. Zha , F. Wang , Y. Wang , and X. Zhang , “Fully Integrated Wearable Humidity Sensor for Respiration Monitoring,” Frontiers in Bioengineering and Biotechnology 10 (2022): 1070855. 36532567 10.3389/fbioe.2022.1070855 PMC9755200

[24] J. Sun , K. Xiu , Z. Wang , et al., “Multifunctional Wearable Humidity and Pressure Sensors Based on Biocompatible Graphene/Bacterial Cellulose Bioaerogel for Wireless Monitoring and Early Warning of Sleep Apnea Syndrome,” Nano Energy 108 (2023): 108215, 10.1016/j.nanoen.2023.108215.

[25] G. Wu , H. Du , Y. L. Cha , et al., “A Wearable Mask Sensor Based on Polyaniline/CNT Nanocomposites for Monitoring Ammonia Gas and Human Breathing,” Sensors and Actuators, B: Chemical 375 (2023): 132858, 10.1016/j.snb.2022.132858.

[26] M. El Gharbi , R. Fernández‐García , and I. Gil , “Embroidered Wearable Antenna‐Based Sensor for Real‐Time Breath Monitoring,” Measurement 195 (2022): 111080, 10.1016/j.measurement.2022.111080.

[27] R. Sasikumar , B. Kim , and R. M. Bhattarai , “Dysprosium Tungstate Incorporated on Exfoliated Layered Molybdenum Disulfide‐Based a Flexible and Wearable Piezoelectric Nanogenerator for the Dual Purpose of Self‐powered Energy Harvesting and a Smart Mask for Human Breath Monitoring,” Nano Energy 118 (2023): 109024, 10.1016/j.nanoen.2023.109024.

[28] J. Dai , J. Meng , X. Zhao , et al., “A Wearable Self‐Powered Multi‐Parameter Respiration Sensor,” Advanced Materials Technologies 8 (2023): 2201535.

[29] P. Jiang , S. Zhao , and R. Zhu , “Smart Sensing Strip Using Monolithically Integrated Flexible Flow Sensor for Noninvasively Monitoring Respiratory Flow,” Sensors 15 (2015): 31738–31750, 10.3390/s151229881. 26694401 PMC4721800

[30] P.‐H. Lin , S.‐C. Sheu , C.‐W. Chen , S.‐C. Huang , and B.‐R. Li , “Wearable Hydrogel Patch With Noninvasive, Electrochemical Glucose Sensor for Natural Sweat Detection,” Talanta 241 (2022): 123187, 10.1016/j.talanta.2021.123187. 35030501

[31] C. Chen , R. Ran , Z. Yang , et al., “An Efficient Flexible Electrochemical Glucose Sensor Based on Carbon Nanotubes/Carbonized Silk Fabrics Decorated With Pt Microspheres,” Sensors and Actuators, B: Chemical 256 (2018): 63–70, 10.1016/j.snb.2017.10.067.

[32] Q. Cao , B. Liang , T. Tu , J. Wei , L. Fang , and X. Ye , “Three‐Dimensional Paper‐Based Microfluidic Electrochemical Integrated Devices (3D‐PMED) for Wearable Electrochemical Glucose Detection,” RSC Advances 9 (2019): 5674–5681. 35515907 10.1039/c8ra09157a PMC9060762

[33] G. Manasa , R. J. Mascarenhas , N. P. Shetti , et al., “Skin Patchable Sensor Surveillance for Continuous Glucose Monitoring,” ACS Applied Bio Materials 5 (2022): 945–970, 10.1021/acsabm.1c01289. 35170319

[34] H.‐J. Kil , S.‐R. Kim , and J.‐W. Park , “A Self‐Charging Supercapacitor for a Patch‐Type Glucose Sensor,” ACS Applied Materials & Interfaces 14 (2022): 3838–3848, 10.1021/acsami.1c21394. 35026107

[35] I. Shitanda , Y. Ozone , Y. Morishita , et al., “Air‐Bubble‐Insensitive Microfluidic Lactate Biosensor for Continuous Monitoring of Lactate in Sweat,” ACS Sensors 8 (2023): 2368–2374, 10.1021/acssensors.3c00490. 37216270 PMC10294251

[36] T. Minami , T. Sato , T. Minamiki , K. Fukuda , D. Kumaki , and S. Tokito , “A Novel OFET‐Based Biosensor for the Selective and Sensitive Detection of Lactate Levels,” Biosensors and Bioelectronics 74 (2015): 45–48, 10.1016/j.bios.2015.06.002. 26101795

[37] M. E. Payne , A. Zamarayeva , V. I. Pister , N. A. D. Yamamoto , and A. C. Arias , “Printed, Flexible Lactate Sensors: Design Considerations before Performing On‐Body Measurements,” Scientific Reports 9 (2019): 13720, 10.1038/s41598-019-49689-7. 31548553 PMC6757068

[38] Z. Ismail , W. F. W. Idris , and A. H. Abdullah , “Graphene‐Based Temperature, Humidity, and Strain Sensor: A Review on Progress, Characterization, and Potential Applications during Covid‐19 Pandemic,” Sensors International 3: 100183, 10.1016/j.sintl.2022.100183. PMC9126002 35633818

[39] S. Kano , N. Jarulertwathana , S. Mohd‐Noor , J. K. Hyun , R. Asahara , and H. Mekaru , “Respiratory Monitoring by Ultrafast Humidity Sensors With Nanomaterials: A Review,” Sensors 22 (2022): 1251, 10.3390/s22031251. 35161997 PMC8838830

[40] H. Jin , Y. S. Abu‐Raya , and H. Haick , “Advanced Materials for Health Monitoring With Skin‐Based Wearable Devices,” Advanced Healthcare Materials 6 (2017): 1700024. 10.1002/adhm.201700024 28371294

[41] Z. Lou , L. Wang , K. Jiang , Z. Wei , and G. Shen , “Reviews of Wearable Healthcare Systems: Materials, Devices and System Integration,” Materials Science and Engineering, R: Reports 140 (2020): 100523, 10.1016/j.mser.2019.100523.

[42] D. Kim , J. Lee , M. K. Park , and S. H. Ko , “Recent Developments in Wearable Breath Sensors for Healthcare Monitoring,” Communications Materials 5 (2024): 41, 10.1038/s43246-024-00480-w.

[43] H. C. Ates and C. Dincer , “Wearable Breath Analysis,” Nature Reviews Bioengineering 1 (2023): 80–82, 10.1038/s44222-022-00011-7.

[44] B. Hu , “Recent Advances in Facemask Devices for In Vivo Sampling of Human Exhaled Breath Aerosols and Inhalable Environmental Exposures,” TrAC, Trends in Analytical Chemistry 151 (2022): 116600, 10.1016/j.trac.2022.116600. 35310778 PMC8917876

[45] L. H. Kwong , R. Wilson , S. Kumar , et al., “Review of the Breathability and Filtration Efficiency of Common Household Materials for Face Masks,” ACS Nano 15 (2021): 5904–5924, 10.1021/acsnano.0c10146. 33822580 PMC8900768

[46] S. Choi , H. Jeon , M. Jang , et al., “Biodegradable, Efficient, and Breathable Multi‐Use Face Mask Filter,” Advanced Science 8 (2021): 2003155. 33747729 10.1002/advs.202003155 PMC7967051

[47] J. T. J. Ju , L. N. Boisvert , and Y. Y. Zuo , “Face Masks against COVID‐19: Standards, Efficacy, Testing and Decontamination Methods,” Advances in Colloid and Interface Science 292 (2021): 102435, 10.1016/j.cis.2021.102435. 33971389 PMC8084286

[48] L. Yang , X. Cao , X. Wang , Q. Wang , and L. Jiao , “Regulative Electronic Redistribution of CoTe_2_/CoP Heterointerfaces for Accelerating Water Splitting,” Applied Catalysis, B: Environmental 329 (2023): 122551, 10.1016/j.apcatb.2023.122551.

[49] J. T. Kim , J. Kwon , H. Lee , et al., “Sunlight‐Driven Self‐Cleaning Ultrafine Particulate Matter Filter With Antibacterial Activity,” ACS Nano 18 (2024): 6387–6397, 10.1021/acsnano.3c11284. 38364103

[50] H. Hameed , M. Usman , A. Tahir , et al., “Pushing the Limits of Remote RF Sensing by Reading Lips Under the Face Mask,” Nature Communications 13 (2022): 5168, 10.1038/s41467-022-32231-1. PMC9452506 36071056

[51] S. Chen , G. Qian , B. Ghanem , et al., “Quantitative and Real‐Time Evaluation of Human Respiration Signals With a Shape‐Conformal Wireless Sensing System,” Advanced Science 9 (2022): 2203460. 36089657 10.1002/advs.202203460 PMC9661834

[52] J. Zhong , Z. Li , M. Takakuwa , et al., “Smart Face Mask Based on an Ultrathin Pressure Sensor for Wireless Monitoring of Breath Conditions (Adv. Mater. 6/2022),” Advanced Materials 34 (2022): 2270048. 10.1002/adma.202107758 34706136

[53] L. Yang , H. Wang , W. Yuan , et al., “Wearable Pressure Sensors Based on MXene/Tissue Papers for Wireless Human Health Monitoring,” ACS Applied Materials & Interfaces 13 (2021): 60531–60543, 10.1021/acsami.1c22001. 34894673

[54] Z. Ye , Y. Ling , M. Yang , et al., “A Breathable, Reusable, and Zero‐Power Smart Face Mask for Wireless Cough and Mask‐Wearing Monitoring,” ACS Nano 16 (2022): 5874–5884, 10.1021/acsnano.1c11041. 35298138

[55] A. Vázquez‐López , J. S. del Río Saez , J. de la Vega , X. Ao , and D.‐Y. Wang , “All‐Fabric Triboelectric Nanogenerator (AF‐TENG) Smart Face Mask: Remote Long‐Rate Breathing Monitoring and Apnea Alarm,” ACS Sensors 8 (2023): 1684–1692. 36976959 10.1021/acssensors.2c02825

[56] Y. Fang , J. Xu , X. Xiao , et al., “A Deep‐Learning‐Assisted‐on‐Mask Sensor Network for Adaptive Respiratory Monitoring,” Advanced Materials 34 (2022): 2200252. 10.1002/adma.202200252 35306703

[57] S. Stankoski , I. Kiprijanovska , I. Mavridou , C. Nduka , H. Gjoreski , and M. Gjoreski , “Breathing Rate Estimation From Head‐Worn Photoplethysmography Sensor Data Using Machine Learning,” Sensors 22 (2022): 2079, 10.3390/s22062079. 35336250 PMC8951087

[58] K. Zhang , Z. Li , J. Zhang , et al., “Biodegradable Smart Face Masks for Machine Learning‐Assisted Chronic Respiratory Disease Diagnosis,” ACS Sensors 7 (2022): 3135–3143, 10.1021/acssensors.2c01628. 36196484

[59] M. Nahal , Y. Brown , and G. Knoesen , 2016 IEEE Wireless Health (WH) (IEEE, 2016), 1–6.

[60] S. Han , J. Kim , Y. Lee , et al., “Transparent Air Filters With Active Thermal Sterilization,” Nano Letters 22 (2022): 524–532, 10.1021/acs.nanolett.1c02737. 34665632

[61] S. Jeong , J. Shin , J. Kim , et al., “Human Circulatory/Respiratory‐Inspired Comprehensive Air Purification System,” Advanced Materials 36 (2024): 2405568. 10.1002/adma.202405568 39140643

[62] J. Shin , S. Jeong , J. Kim , et al., “Dynamic Pore Modulation of Stretchable Electrospun Nanofiber Filter for Adaptive Machine Learned Respiratory Protection,” ACS Nano 15 (2021): 15730–15740, 10.1021/acsnano.1c06204. 34585584

[63] K. Kwon , Y. J. Lee , Y. Jung , et al., “Smart Filtering Facepiece Respirator With Self‐Adaptive Fit and Wireless Humidity Monitoring,” Biomaterials 314 (2025): 122866, 10.1016/j.biomaterials.2024.122866. 39342918

[64] A. Zakrzewska , M. A. Haghighat Bayan , P. Nakielski , F. Petronella , L. De Sio , and F. Pierini , “Nanotechnology Transition Roadmap toward Multifunctional Stimuli‐Responsive Face Masks,” ACS Applied Materials & Interfaces 14 (2022): 46123–46144, 10.1021/acsami.2c10335. 36161869

[65] F. Seidi , C. Deng , Y. Zhong , et al., “Functionalized Masks: Powerful Materials Against COVID‐19 and Future Pandemics,” Small 17 (2021): 2102453. 34319644 10.1002/smll.202102453 PMC8420174

[66] W. Deng , Y. Sun , X. Yao , et al., “Masks for COVID‐19,” Advanced Science 9 (2022): 2102189. 34825783 10.1002/advs.202102189 PMC8787406

[67] A. Tuñón‐Molina , K. Takayama , E. M. Redwan , V. N. Uversky , J. Andrés , and Á. Serrano‐Aroca , “Protective Face Masks: Current Status and Future Trends,” ACS Applied Materials & Interfaces 13 (2021): 56725–56751, 10.1021/acsami.1c12227. 34797624

[68] H. Xie , J. Du , W. Han , J. Tang , X. Li , and J. Chen , “Occurrence and Health Risks of Semi‐Volatile Organic Compounds in Face Masks,” Science Bulletin 66 (2021): 1601–1603, 10.1016/j.scib.2021.04.009. 36408477 PMC9666228

[69] V. Palmieri , F. De Maio , M. De Spirito , and M. Papi , “Face Masks and Nanotechnology: Keep the Blue Side Up,” Nano Today 37 (2021): 101077, 10.1016/j.nantod.2021.101077. 33519950 PMC7833187

[70] J. Liang , C. Liu , and T. Xu , “Innovative Biosensing Smart Masks: Unveiling the Future of Respiratory Monitoring,” Materials Horizons 12 (2025): 5975–5993, 10.1039/D5MH00279F. 40384465

[71] Y. Yang , X. Yang , Y. Tan , and Q. Yuan , “Recent Progress in Flexible and Wearable Bio‐Electronics Based on Nanomaterials,” Nano Research 10 (2017): 1560–1583, 10.1007/s12274-017-1476-8.

[72] B. Peng , F. Zhao , J. Ping , and Y. Ying , “Recent Advances in Nanomaterial‐Enabled Wearable Sensors: Material Synthesis, Sensor Design, and Personal Health Monitoring,” Small 16 (2020): 2002681. 10.1002/smll.202002681 32893485

[73] Ö. Erdem , E. Derin , S. Zeibi Shirejini , et al., “Carbon‐Based Nanomaterials and Sensing Tools for Wearable Health Monitoring Devices,” Advanced Materials Technologies 7 (2022): 2100572.

[74] M. Hassan , G. Abbas , N. Li , et al., “Significance of Flexible Substrates for Wearable and Implantable Devices: Recent Advances and Perspectives,” Advanced Materials Technologies 7 (2022): 2100773.

[75] H. Yoon , J. Choi , J. Kim , et al., “Adaptive Epidermal Bioelectronics by Highly Breathable and Stretchable Metal Nanowire Bioelectrodes on Electrospun Nanofiber Membrane,” Advanced Functional Materials 34 (2024): 2313504.

[76] Y. Jung , S. Jeong , J. Ahn , J. Lee , and S. H. Ko , “High Efficiency Breathable Thermoelectric Skin Using Multimode Radiative Cooling/Solar Heating Assisted Large Thermal Gradient,” Small 20 (2024): 2304338. 10.1002/smll.202304338 37649174

[77] Y. Zhao , R. Chen , R. Ni , H. Liu , J. Li , and C. Huang , “Fabrication and Characterization of a Novel Facial Mask Substrates Based on Thermoplastic Polyester Elastomer Fibers,” Journal of The Textile Institute 111 (2020): 1231–1237, 10.1080/00405000.2019.1702612.

[78] A. Sinha , A. K. Stavrakis , M. Simić , and G. M. Stojanović , “Polymer‐Thread‐Based Fully Textile Capacitive Sensor Embroidered on a Protective Face Mask for Humidity Detection,” ACS Omega 7 (2022): 44928–44938, 10.1021/acsomega.2c05162. 36530326 PMC9753493

[79] R. Cao , X. Pu , X. Du , et al., “Screen‐Printed Washable Electronic Textiles as Self‐Powered Touch/Gesture Tribo‐Sensors for Intelligent Human–Machine Interaction,” ACS Nano 12 (2018): 5190–5196, 10.1021/acsnano.8b02477. 29771494

[80] Y. Hao , M. Tian , H. Zhao , et al., “High Efficiency Electrothermal Graphene/Tourmaline Composite Fabric Joule Heater With Durable Abrasion Resistance via a Spray Coating Route,” Industrial & Engineering Chemistry Research 57 (2018): 13437–13448, 10.1021/acs.iecr.8b03628.

[81] L. Liu , W. Chen , H. Zhang , Q. Wang , F. Guan , and Z. Yu , “Flexible and Multifunctional Silk Textiles with Biomimetic Leaf‐Like MXene/Silver Nanowire Nanostructures for Electromagnetic Interference Shielding, Humidity Monitoring, and Self‐Derived Hydrophobicity,” Advanced Functional Materials 29 (2019): 1905197.

[82] A. Chen , A. J. Halton , R. D. Rhoades , et al., “Wireless Wearable Ultrasound Sensor on a Paper Substrate to Characterize Respiratory Behavior,” ACS Sensors 4 (2019): 944–952. 30855133 10.1021/acssensors.9b00043

[83] M. Parrilla , T. Guinovart , J. Ferré , P. Blondeau , and F. J. Andrade , “A Wearable Paper‐Based Sweat Sensor for Human Perspiration Monitoring,” Advanced Healthcare Materials 8 (2019): 1900342. 10.1002/adhm.201900342 31293084

[84] J. Ahn , J.‐W. Seo , T.‐I. Lee , et al., “Extremely Robust and Patternable Electrodes for Copy‐Paper‐Based Electronics,” ACS Applied Materials & Interfaces 8 (2016): 19031–19037, 10.1021/acsami.6b05296. 27378213

[85] S. Chen , Y. Song , D. Ding , Z. Ling , and F. Xu , “Flexible and Anisotropic Strain Sensor Based on Carbonized Crepe Paper with Aligned Cellulose Fibers,” Advanced Functional Materials 28 (2018): 1802547.

[86] J. Kim , H. Roh , S. Moon , et al., “Wireless Breathable Face Mask Sensor for Spatiotemporal 2D Respiration Profiling and Respiratory Diagnosis,” Biomaterials 309 (2024): 122579, 10.1016/j.biomaterials.2024.122579. 38670033

[87] S. Wang , Y. Jiang , H. Tai , et al., “An Integrated Flexible Self‐Powered Wearable Respiration Sensor,” Nano Energy 63 (2019): 103829, 10.1016/j.nanoen.2019.06.025.

[88] J. Oliveira , V. Correia , H. Castro , P. Martins , and S. Lanceros‐Mendez , “Polymer‐Based Smart Materials by Printing Technologies: Improving Application and Integration,” Additive Manufacturing 21 (2018): 269.

[89] S. Gong , W. Schwalb , Y. Wang , et al., “A Wearable and Highly Sensitive Pressure Sensor With Ultrathin Gold Nanowires,” Nature Communications 5 (2014): 3132, 10.1038/ncomms4132. 24495897

[90] A. del Bosque , X. X. Fernández Sánchez‐Romate , Á. Da La Llana Calvo , et al., “Highly Flexible Strain Sensors Based on CNT‐Reinforced Ecoflex Silicone Rubber for Wireless Facemask Breathing Monitoring via Bluetooth,” ACS Applied Polymer Materials 5 (2023): 8589–8599.

[91] T. Hu and B. Sheng , “A Highly Sensitive Strain Sensor with Wide Linear Sensing Range Prepared on a Hybrid‐Structured CNT/Ecoflex Film via Local Regulation of Strain Distribution,” ACS Appl Mater Interfaces 16, no. 16 (2024): 21061–21072. 38603806 10.1021/acsami.4c00648

[92] H. Savari and A. Nikfarjam , “Design and Fabrication of Patterned Flexible Electrode Using DC Sputtering and Shadow Mask,” Engineering Research Express 6: 045318, 10.1088/2631-8695/ad833a.

[93] Y. Guo , W. Li , H. Yu , D. F. Perepichka , and H. Meng , “Flexible Asymmetric Supercapacitors via Spray Coating of a New Electrochromic Donor–Acceptor Polymer,” Advanced Energy Materials 7 (2017): 1601623.

[94] T.‐E. Lin , M.‐C. Chien , P.‐F. Chen , et al., “A Sensor‐Integrated Face Mask Using Au@SnO_2_ Nanoparticle Modified Fibers and Augmented Reality Technology,” ACS Omega 7 (2022): 42233–42241. 36440160 10.1021/acsomega.2c04655 PMC9685760

[95] Z. Humphreys Salas , A. F. Martínez Ávila , M. M. Hernández Orozco , et al., “Green Synthesis of Copper Nanoparticles and Their Formulation into Face Masks: An Antibacterial Study,” Polymer Composites 44 (2023): 907–916, 10.1002/pc.27142.

[96] T. Qi , C. Chen , Z. Yong , X. Gong , and S. Ramakrishna , “A Robust Bridge‐Type Airflow Sensor Based on Flexible Superhydrophobic Carbon Nanotube Fiber Thin Films,” Advanced Materials Interfaces 11 (2024): 2300077.

[97] C. Ding , Y. Liu , P. Xie , et al., “A Novel Carbon Aerogel Enabling respiratory Monitoring for Bio‐Facial Masks,” Journal of Materials Chemistry A 9 (2021): 13143–13150, 10.1039/D1TA00794G.

[98] J. Suo , Y. Liu , C. Wu , et al., “Wide‐Bandwidth Nanocomposite‐Sensor Integrated Smart Mask for Tracking Multiphase Respiratory Activities,” Advanced Science 9 (2022): 2203565. 35999427 10.1002/advs.202203565 PMC9631096

[99] G. Chen , R. Guan , M. Shi , et al., “A Nanoforest‐Based Humidity Sensor for Respiration Monitoring,” Microsystems & Nanoengineering 8 (2022): 44, 10.1038/s41378-022-00372-4. 35498335 PMC9023489

[100] X. Wang , Y. Deng , X. Chen , P. Jiang , Y. K. Cheung , and H. Yu , “An Ultrafast‐Response and Flexible Humidity Sensor for Human Respiration Monitoring and Noncontact Safety Warning,” Microsystems & Nanoengineering 7 (2021): 99. 34900333 10.1038/s41378-021-00324-4 PMC8628006

[101] M. Wang , N. Li , G.‐D. Wang , S. W. Lu , Q. Di Zhao , and X. L. Liu , “High‐Sensitive Flexural Sensors for Health Monitoring of Composite Materials Using Embedded Carbon Nanotube (CNT) Buckypaper,” Composite Structures 261 (2021): 113280, 10.1016/j.compstruct.2020.113280.

[102] P. V. Adhyapak , A. M. Kasabe , A. D. Bang , J. Ambekar , and S. K. Kulkarni , “Highly Sensitive, Room Temperature Operated Gold Nanowire‐based Humidity Sensor: Adoptable for Breath Sensing,” RSC Advances 12 (2022): 1157–1164. 35425134 10.1039/d1ra07510a PMC8978864

[103] A. Nag , R. B. V. B. Simorangkir , D. R. Gawade , et al., “Graphene‐Based Wearable Temperature Sensors: A Review,” Materials & Design 221 (2022): 110971, 10.1016/j.matdes.2022.110971.

[104] K.‐Y. Chen , Y.‐T. Xu , Y. Zhao , J.‐K. Li , X.‐P. Wang , and L.‐T. Qu , “Recent Progress in Graphene‐based Wearable Piezoresistive Sensors: From 1D to 3D Device Geometries,” Nano Materials Science 5 (2023): 247–264, 10.1016/j.nanoms.2021.11.003.

[105] Y. Wang , L. Zhang , Z. Zhang , P. Sun , and H. Chen , “High‐Sensitivity Wearable and Flexible Humidity Sensor Based on Graphene Oxide/Non‐Woven Fabric for Respiration Monitoring,” Langmuir 36 (2020): 9443–9448, 10.1021/acs.langmuir.0c01315. 32693594

[106] R. Zhao , D. Xie , S. Qing , et al., “Cellulose Acetate and Reduced Graphene Oxide (rGO)‐Based Flexible Humidity Sensor for Monitoring Human Respiration,” Sensors and Actuators, B: Chemical 429 (2025): 137291, 10.1016/j.snb.2025.137291.

[107] H. C. Bidsorkhi , N. Faramarzi , B. Ali , et al., “Wearable Graphene‐Based Smart Face Mask for Real‐Time human Respiration Monitoring,” Materials & Design 230 (2023): 111970, 10.1016/j.matdes.2023.111970. 37162811 PMC10151252

[108] L. Yang , H. Wang , A. M. Abdullah , et al., “Direct Laser Writing of the Porous Graphene Foam for Multiplexed Electrochemical Sweat Sensors,” ACS Applied Materials & Interfaces 15 (2023): 34332–34342, 10.1021/acsami.3c02485. 37433119

[109] S. Chowdhury , A. Roy , C. Liu , et al., “Two‐Step Growth of Uniform Monolayer MoS_2_ Nanosheets by Metal–Organic Chemical Vapor Deposition,” ACS Omega 6 (2021): 10343–10351. 34056187 10.1021/acsomega.1c00727 PMC8153749

[110] J. N. Coleman , M. Lotya , A. O'Neill , et al., “Two‐Dimensional Nanosheets Produced by Liquid Exfoliation of Layered Materials,” Science (1979). 331 (2011): 568–571, 10.1126/science.1194975. 21292974

[111] M. Xu , J. Gao , J. Song , et al., “Programmable Patterned MoS_2_ Film by Direct Laser Writing for Health‐Related Signals Monitoring,” IScience 24 (2021): 103313. 34755102 10.1016/j.isci.2021.103313 PMC8564106

[112] T. Afaneh , P. K. Sahoo , I. A. P. Nobrega , Y. Xin , and H. R. Gutiérrez , “Laser‐Assisted Chemical Modification of Monolayer Transition Metal Dichalcogenides,” Advanced Functional Materials 28 (2018): 1802949.

[113] J. Zhang , A. Yang , X. Wu , et al., “Reversible and Selective Ion Intercalation through the Top Surface of Few‐Layer MoS_2_ ,” Nature Communications 9 (2018): 5289, 10.1038/s41467-018-07710-z. PMC6290021 30538249

[114] E. Benavente , “Intercalation Chemistry of Molybdenum Disulfide,” Coordination Chemistry Reviews 224 (2002): 87–109, 10.1016/S0010-8545(01)00392-7.

[115] X. Zhan , C. Si , J. Zhou , and Z. Sun , “MXene and MXene‐Based Composites: Synthesis, Properties and Environment‐related Applications,” Nanoscale Horizons 5 (2020): 235–258, 10.1039/C9NH00571D.

[116] Y. Liang , Q. Ding , H. Wang , et al., “Humidity Sensing of Stretchable and Transparent Hydrogel Films for Wireless Respiration Monitoring,” Nano‐Micro Letters 14 (2022): 183. 36094761 10.1007/s40820-022-00934-1 PMC9468213

[117] J. Liu , H. Wang , T. Liu , et al., “Multimodal Hydrogel‐Based Respiratory Monitoring System for Diagnosing Obstructive Sleep Apnea Syndrome,” Advanced Functional Materials 32 (2022): 2204686.

[118] D. K. Rajak , D. D. Pagar , R. Kumar , and C. I. Pruncu , “Recent Progress of Reinforcement Materials: A Comprehensive Overview of Composite Materials,” Journal of Materials Research and Technology 8 (2019): 6354–6374, 10.1016/j.jmrt.2019.09.068.

[119] H.‐S. Kim , J.‐H. Kang , J.‐Y. Hwang , and U. S. Shin , “Wearable CNTs‐Based Humidity Sensors With High Sensitivity and Flexibility for Real‐Time Multiple respiratory Monitoring,” Nano Convergence 9 (2022): 35, 10.1186/s40580-022-00326-6. 35913549 PMC9343523

[120] L. Ma , Q. Liu , R. Wu , et al., “From Molecular Reconstruction of Mesoscopic Functional Conductive Silk Fibrous Materials to Remote Respiration Monitoring,” Small 16 (2020): 2000203. 10.1002/smll.202000203 32452630

[121] H. Hu , S. Shang , J. Liu , and P. Zhu , “Silk Fibroin Based Flexible and Self‐Powered Sensor for Real‐Time Monitoring of Abdominal Respiration,” International Journal of Biological Macromolecules 254 (2024): 127723, 10.1016/j.ijbiomac.2023.127723. 37907181

[122] M. Zhu , Y. Deng , Y. Zheng , et al., “Tribo‐Charge Enhanced and Cellulose Based Biodegradable Nanofibrous Membranes With Highly Fluffy Structure for Air Filtration and Self‐Powered Respiration Monitoring Systems,” Journal of Hazardous Materials 468 (2024): 133770, 10.1016/j.jhazmat.2024.133770. 38401212

[123] C. M. Clarkson , S. M. El Awad Azrak , E. S. Forti , G. T. Schueneman , R. J. Moon , and J. P. Youngblood , “Recent Developments in Cellulose Nanomaterial Composites,” Advanced Materials 33 (2021): 2000718. 10.1002/adma.202000718 32696496

[124] O. Nechyporchuk , M. N. Belgacem , and J. Bras , “Production of Cellulose Nanofibrils: A Review of Recent Advances,” Industrial Crops and Products 93 (2016): 2–25, 10.1016/j.indcrop.2016.02.016.

[125] S. Mekid and K. Chenaoua , “IoT‐Enabled Smart Mask for Monitoring Body Parameters and Location through Cloud,” Internet of Things 22: 100794, 10.1016/j.iot.2023.100794. PMC10121153 37266184

[126] J. Hyysalo , S. Dasanayake , J. Hannu , et al., “Smart Mask – Wearable IoT Solution for Improved Protection and Personal Health,” Internet of Things 18: 100511, 10.1016/j.iot.2022.100511. PMC8875770 37521492

[127] M. Amjadi , K. Kyung , I. Park , and M. Sitti , “Stretchable, Skin‐Mountable, and Wearable Strain Sensors and Their Potential Applications: A Review,” Advanced Functional Materials 26 (2016): 1678–1698, 10.1002/adfm.201504755.

[128] C. N. S. Micheal and J. Mcgrath , Sensor Technologies Healthcare, Wellness and Environmental Applications (Springer, 2013).

[129] S. Z. Homayounfar and T. L. Andrew , “Wearable Sensors for Monitoring Human Motion: A Review on Mechanisms, Materials, and Challenges,” SLAS Technology 25 (2020): 9–24. 31829083 10.1177/2472630319891128

[130] H. Farahani , R. Wagiran , and M. Hamidon , “Humidity Sensors Principle, Mechanism, and Fabrication Technologies: A Comprehensive Review,” Sensors 14 (2014): 7881–7939, 10.3390/s140507881. 24784036 PMC4063076

[131] T. Tatara and K. Tsuzaki , “An Apnea Monitor Using a Rapid‐Response Hygrometer,” Journal of Clinical Monitoring 13 (1997): 5–9, 10.1023/A:1007380021895. 9058247

[132] Y. Lu , G. Yang , Y. Shen , H. Yang , and K. Xu , “Multifunctional Flexible Humidity Sensor Systems Towards Noncontact Wearable Electronics,” Nano‐Micro Letters 14 (2022): 150. 35869398 10.1007/s40820-022-00895-5 PMC9307709

[133] D. Zhang , Z. Xu , Z. Yang , and X. Song , “High‐Performance Flexible Self‐Powered Tin Disulfide Nanoflowers/Reduced Graphene Oxide Nanohybrid‐based Humidity Sensor Driven by Triboelectric Nanogenerator,” Nano Energy 67 (2020): 104251, 10.1016/j.nanoen.2019.104251.

[134] D. Burman , S. Santra , P. Pramanik , and P. K. Guha , “Pt Decorated MoS_2_ Nanoflakes for Ultrasensitive Resistive Humidity Sensor,” Nanotechnology 29 (2018): 115504, 10.1088/1361-6528/aaa79d. 29408801

[135] D. Burman , R. Ghosh , S. Santra , and P. K. Guha , “Highly Proton Conducting MoS_2_/Graphene Oxide Nanocomposite Based Chemoresistive Humidity Sensor,” RSC Advances 6 (2016): 57424–57433.

[136] Q. Ding , H. Wang , Z. Zhou , et al., “Stretchable, Self‐Healable, and Breathable Biomimetic Iontronics with Superior Humidity‐Sensing Performance for Wireless Respiration Monitoring,” SmartMat 4 (2023): 1147.

[137] M. Zhang , M. Wang , M. Zhang , et al., “Flexible and Highly Sensitive Humidity Sensor Based on Sandwich‐Like Ag/Fe_3_O_4_ Nanowires Composite for Multiple Dynamic Monitoring,” Nanomaterials 9 (2019): 1399, 10.3390/nano9101399. 31581599 PMC6835934

[138] S. Kundu , R. Majumder , R. Ghosh , et al., “Relative Humidity Sensing Properties of Doped Polyaniline‐Encased Multiwall Carbon Nanotubes: Wearable and Flexible human Respiration Monitoring Application,” Journal of Materials Science 55 (2020): 3884–3901, 10.1007/s10853-019-04276-z.

[139] L. Lu , C. Jiang , G. Hu , J. Liu , and B. Yang , “Flexible Noncontact Sensing for Human–Machine Interaction,” Advanced Materials 33 (2021): 2100218. 10.1002/adma.202100218 33683745

[140] S. Ding , Y. Lou , Z. Niu , et al., “A Highly Sensitive, Breathable, and Biocompatible Wearable Sensor Based on Nanofiber Membrane for Pressure and Humidity Monitoring,” Macromolecular Materials and Engineering 307 (2022): 2200233.

[141] T. Liu , Y. Rong , G. Zhou , et al., “Direct‐Writing MXene/Polypropylene Composites for Wearable Humidity Sensors With Multiple Applications,” IEEE Sensors Journal 24 (2024): 15241–15251, 10.1109/JSEN.2024.3376355.

[142] H. Xing , X. Li , Y. Lu , et al., “MXene/MWCNT Electronic Fabric With Enhanced Mechanical Robustness on Humidity Sensing for Real‐time Respiration Monitoring,” Sensors and Actuators, B: Chemical 361 (2022): 131704, 10.1016/j.snb.2022.131704.

[143] A. Sinha , A. K. Stavrakis , M. Simić , and G. M. Stojanović , “Wearable Humidity Sensor Embroidered on a Commercial Face Mask and Its Electrical Properties,” Journal of Materials Science 58 (2023): 1680–1693, 10.1007/s10853-022-08135-2. 36687141 PMC9838397

[144] P. Srikrishnarka , R. M. Dasi , S. K. Jana , et al., “Toward Continuous Breath Monitoring on a Mobile Phone Using a Frugal Conducting Cloth‐Based Smart Mask,” ACS Omega 7 (2022): 42926–42938, 10.1021/acsomega.2c05017. 36467907 PMC9713799

[145] C.‐T. Li , M.‐X. Chong , L.‐X. Zhang , B. Tang , and L.‐J. Bie , “All‐inorganic Lead‐Free Halide Perovskite Cs_2_TeBr_6_ Enables Real‐Time Touchless human Breath and Finger Related Humidity Monitoring,” Sensors and Actuators, B: Chemical 379 (2023): 133240, 10.1016/j.snb.2022.133240.

[146] L. K. Allison , S. Rostaminia , A. Kiaghadi , D. Ganesan , and T. L. Andrew , “Enabling Longitudinal Respiration Monitoring Using Vapor‐Coated Conducting Textiles,” ACS Omega 6 (2021): 31869–31875. 34870009 10.1021/acsomega.1c04616 PMC8638004

[147] S. Yu , C. Chen , H. Zhang , J. Zhang , and J. Liu , “Design of High Sensitivity Graphite Carbon Nitride/Zinc Oxide Humidity Sensor for Breath Detection,” Sensors and Actuators, B: Chemical 332 (2021): 129536, 10.1016/j.snb.2021.129536.

[148] Y. Wang , L. Zhang , J. Zhou , and A. Lu , “Flexible and Transparent Cellulose‐Based Ionic Film as a Humidity Sensor,” ACS Applied Materials & Interfaces 12 (2020): 7631–7638, 10.1021/acsami.9b22754. 31961643

[149] C.‐H. Su , H.‐L. Chiu , Y.‐C. Chen , et al., “Highly Responsive PEG/Gold Nanoparticle Thin‐Film Humidity Sensor via Inkjet Printing Technology,” Langmuir 35 (2019): 3256–3264, 10.1021/acs.langmuir.8b03433. 30607954

[150] X. Huang , B. Li , L. Wang , X. Lai , H. Xue , and J. Gao , “Superhydrophilic, Underwater Superoleophobic, and Highly Stretchable Humidity and Chemical Vapor Sensors for Human Breath Detection,” ACS Applied Materials & Interfaces 11 (2019): 24533–24543, 10.1021/acsami.9b04304. 31246404

[151] R. Xie , Q. Du , B. Zou , et al., “Wearable Leather‐Based Electronics for Respiration Monitoring,” ACS Applied Bio Materials 2 (2019): 1427–1431, 10.1021/acsabm.9b00082. 35026917

[152] Z. Duan , Y. Jiang , M. Yan , et al., “Facile, Flexible, Cost‐Saving, and Environment‐Friendly Paper‐Based Humidity Sensor for Multifunctional Applications,” ACS Applied Materials & Interfaces 11 (2019): 21840–21849, 10.1021/acsami.9b05709. 31135126

[153] J. Zhang , X.‐X. Wang , B. Zhang , et al., “In Situ Assembly of Well‐Dispersed Ag Nanoparticles throughout Electrospun Alginate Nanofibers for Monitoring Human Breath—Smart Fabrics,” ACS Applied Materials & Interfaces 10 (2018): 19863–19870, 10.1021/acsami.8b01718. 29782141

[154] J. He , P. Xiao , J. Shi , et al., “High Performance Humidity Fluctuation Sensor for Wearable Devices via a Bioinspired Atomic‐Precise Tunable Graphene–Polymer Heterogeneous Sensing Junction,” Chemistry of Materials 30 (2018): 4343–4354, 10.1021/acs.chemmater.8b01587.

[155] A. Beniwal , G. Khandelwal , R. Mukherjee , D. M. Mulvihill , and C. Li , “Eco‐Friendly Textile‐Based Wearable Humidity Sensor With Multinode Wireless Connectivity for Healthcare Applications,” ACS Applied Bio Materials 7 (2024): 4772–4784, 10.1021/acsabm.4c00593. PMC11253092 38963128

[156] T. Liu , D. Qu , L. Guo , et al., “MXene/TPU Composite Film for Humidity Sensing and Human Respiration Monitoring,” Advanced Sensor Research 3 (2024): 2300014.

[157] S. Yu , C. Chen , P. Li , H. Zhang , and H. Zhang , “Highly Sensitive Ti_3_C_2_T* _x_ * MXenes–RGO Humidity Sensor for human Non‐Contact Respiratory Monitoring,” Sensors and Actuators, B: Chemical 401 (2024): 135014, 10.1016/j.snb.2023.135014.

[158] A. M. Ramesh , M. Rajesh , A. Chandran , and K. P. Surendran , “Gold‐Nanoparticle‐Based Flexible Humidity Sensor for Breath Monitoring and Smart Irrigation Systems,” ACS Applied Nano Materials 7 (2024): 15593–15605.

[159] M. Deb , M.‐Y. Chen , P.‐Y. Chang , et al., “SnO_2_‐Based Ultra‐Flexible Humidity/Respiratory Sensor for Analysis of Human Breath,” Biosensors 13 (2023): 81, 10.3390/bios13010081. 36671916 PMC9856198

[160] J. Qin , X. Yang , C. Shen , et al., “Carbon Nanodot‐based Humidity Sensor for Self‐Powered Respiratory Monitoring,” Nano Energy 101 (2022): 107549, 10.1016/j.nanoen.2022.107549.

[161] Y. Jiang , L. Wu , Q. Chen , N. Li , and J. Tian , “High‐Performance Capacitive Humidity Sensor Based on Flower‐Like SnS_2_/Ti_3_C_2_ MXene for Respiration Monitoring and Non‐Contact Sensing,” Sensors and Actuators, B: Chemical 426 (2025): 137012, 10.1016/j.snb.2024.137012.

[162] L. Ma , A. Patil , R. Wu , et al., “A Capacitive Humidity Sensor Based on All‐Protein Embedded With Gold Nanoparticles@Carbon Composite for Human Respiration Detection,” Nanotechnology 32 (2021): 19LT01. 10.1088/1361-6528/abe32d 33540394

[163] D.‐L. Wen , Y.‐X. Pang , P. Huang , et al., “Silk Fibroin‐Based Wearable All‐Fiber Multifunctional Sensor for Smart Clothing,” Advanced Fiber Materials 4 (2022): 873–884, 10.1007/s42765-022-00150-x.

[164] A. Ullah , M. H. Zulfiqar , M. A. Khan , Y. Massoud , M. Zubair , and M. Q. Mehmood , “Fast‐Response Humidity Sensor‐Based Smart Face Mask for Multifunctional Applications,” Sensing and Imaging 25 (2024): 43, 10.1007/s11220-024-00498-x.

[165] L. Ma , R. Wu , A. Patil , et al., “Full‐Textile Wireless Flexible Humidity Sensor for Human Physiological Monitoring,” Advanced Functional Materials 29 (2019): 1904549.

[166] D. Shen , Y. Liu , M. Yu , F. Kong , B. Xin , and Y. Liu , “Bioinspired Flexible and Highly Responsive PVDF‐Based Humidity Sensors for Respiratory Monitoring,” Polymer 254 (2022): 125103, 10.1016/j.polymer.2022.125103.

[167] R. Ghosh , M. S. Song , J. Park , et al., “Fabrication of Piezoresistive Si Nanorod‐Based Pressure Sensor Arrays: A Promising Candidate for Portable Breath Monitoring Devices,” Nano Energy 80 (2021): 105537, 10.1016/j.nanoen.2020.105537.

[168] A. DeHennis , J. Chae , and A. Baroutaji , Reference Module in Materials Science and Materials Engineering (Elsevier, 2016).

[169] 2019 BIPM.LeSystèmeinternationald'unités/TheInternationalSystemofUnits(‘TheSIBrochure’).Bureauinternationaldespoidsetmesures,Ninthedition, 2019. http://www.bipm.org/en/si/si_brochure/, ISBN978‐92‐822‐2272‐0.

[170] A. M. Baig , “Computing the Effects of SARS‐CoV‐2 on Respiration Regulatory Mechanisms in COVID‐19,” ACS Chemical Neuroscience 11 (2020): 2416–2421, 10.1021/acschemneuro.0c00349. 32600045 PMC7422910

[171] T. Zhang , Y. Zhao , Q. Long , et al., “Graphene/MXene/Cellulose Cellulosic Paper‐Based Flexible Bifunctional Sensors Utilizing Molecular Bridge Strategy With Tunable Piezoresistive Effect for Temperature‐Pressure Sensing,” Chemical Engineering Journal 497 (2024): 154972, 10.1016/j.cej.2024.154972.

[172] X. Zheng , S. Zhang , M. Zhou , et al., “MXene Functionalized, Highly Breathable and Sensitive Pressure Sensors with Multi‐Layered Porous Structure,” Advanced Functional Materials 33 (2023): 2214880, 10.1002/adfm.202214880.

[173] Y. Zhou , L. Zhao , W. Tao , et al., “All‐Nanofiber Network Structure for Ultrasensitive Piezoresistive Pressure Sensors,” ACS Applied Materials & Interfaces 14 (2022): 19949–19957, 10.1021/acsami.1c24257. 35446539

[174] H. Choi , J. Sun , B. Ren , et al., “3D Textile Structure‐Induced Local Strain for a Highly Amplified Piezoresistive Performance of Carbonized Cellulose Fabric Based Pressure Sensor for human Healthcare Monitoring,” Chemical Engineering Journal 450 (2022): 138193, 10.1016/j.cej.2022.138193.

[175] J. Hwang , Y. Kim , H. Yang , and J. H. Oh , “Fabrication of Hierarchically Porous Structured PDMS Composites and Their Application as a Flexible Capacitive Pressure Sensor,” Composites, Part B: Engineering 211 (2021): 108607, 10.1016/j.compositesb.2021.108607.

[176] R. Qin , M. Hu , X. Li , et al., “A New Strategy for the Fabrication of a Flexible and Highly Sensitive Capacitive Pressure Sensor,” Microsystems & Nanoengineering 7 (2021): 100, 10.1038/s41378-021-00327-1. 34868631 PMC8630520

[177] M. Ren , J. Li , L. Lv , et al., “A Wearable and High‐Performance Capacitive Pressure Sensor Based on a Biocompatible PVP Nanofiber Membrane via Electrospinning and UV Treatment,” Journal of Materials Chemistry C: Materials for Optical and Electronic Devices 10 (2022): 10491–10499, 10.1039/D2TC00955B.

[178] X. Weng , C. Zhang , C. Feng , and H. Jiang , “Facile Fabrication of an Ultrasensitive All‐Fabric Wearable Pressure Sensor Based on Phosphorene‐Gold Nanocomposites,” Advanced Materials Interfaces 9 (2022): 2102588, 10.1002/admi.202102588.

[179] Z. Zhang , Y. Zhang , X. Jiang , et al., “Simple and Efficient Pressure Sensor Based on PDMS Wrapped CNT Arrays,” Carbon 155 (2019): 71–76, 10.1016/j.carbon.2019.08.018.

[180] L. Liao , S. Zhou , J. Yang , et al., “A Flexible Pressure Sensor Based on an Interlocked Micropillars Array With Secondary Nanoprotrusions for Health Monitoring,” IEEE Transactions on Instrumentation and Measurement 73 (2024): 1, 10.1109/TIM.2024.3378283.

[181] C. C. Vu and J. Kim , “Waterproof, Thin, High‐performance Pressure Sensors‐Hand Drawing for Underwater Wearable Applications,” Science and Technology of Advanced Materials 22 (2021): 718–728, 10.1080/14686996.2021.1961100. 34434076 PMC8381950

[182] S. Sharma , A. Chhetry , P. Maharjan , et al., “Polyaniline‐Nanospines Engineered Nanofibrous Membrane Based Piezoresistive Sensor for High‐Performance Electronic Skins,” Nano Energy 95 (2022): 106970, 10.1016/j.nanoen.2022.106970.

[183] W. Yang , N.‐W. Li , S. Zhao , et al., “A Breathable and Screen‐Printed Pressure Sensor Based on Nanofiber Membranes for Electronic Skins,” Advanced Materials Technologies 3 (2018): 1700241, 10.1002/admt.201700241.

[184] S. Lu , T. Nie , Y. Li , et al., “A Highly Sensitive Flexible Pressure Sensor Based on Inter‐Comb Structured Graphene Electrodes,” IEEE Transactions on Electron Devices 70 (2023): 1865–1870, 10.1109/TED.2023.3248000.

[185] V. Palaniappan , M. Panahi , D. Maddipatla , et al., “Flexible M‐Tooth Hybrid Micro‐Structure‐Based Capacitive Pressure Sensor With High Sensitivity and Wide Sensing Range,” IEEE Sensors Journal 21 (2021): 26261–26268, 10.1109/JSEN.2021.3064451.

[186] H. Jiang , X. Zou , P. Li , Z. Lin , X. Liu , and X. Weng , “Flexible Capacitive Pressure Sensor Enhanced by Black Phosphorus–Au Nanocomposites/Ecoflex Sponge for Pressure and Proximity Detection,” IEEE Sensors Journal 24 (2024): 17543–17550, 10.1109/JSEN.2024.3391340.

[187] W. Asghar , F. Li , Y. Zhou , et al., “Piezocapacitive Flexible E‐Skin Pressure Sensors Having Magnetically Grown Microstructures,” Advanced Materials Technologies 5 (2020): 1900934, 10.1002/admt.201900934.

[188] J. Zhong , Z. Li , M. Takakuwa , et al., “Smart Face Mask Based on an Ultrathin Pressure Sensor for Wireless Monitoring of Breath Conditions,” Advanced Materials 34 (2022): 2107758, 10.1002/adma.202107758. 34706136

[189] Z. Yin , Y. Yang , C. Hu , J. Li , B. Qin , and X. Yang , “Wearable respiratory Sensors for Health Monitoring,” NPG Asia Materials 16 (2024): 8.

[190] F. Zheng , H.‐Y. Jiang , X.‐T. Yang , et al., “Reviews of Wearable Healthcare Systems Based on Flexible Gas Sensors,” Chemical Engineering Journal 490 (2024): 151874, 10.1016/j.cej.2024.151874.

[191] X. Zhou , X. Cheng , Y. Zhu , et al., “Ordered Porous Metal Oxide Semiconductors for Gas Sensing,” Chinese Chemical Letters 29 (2018): 405–416, 10.1016/j.cclet.2017.06.021.

[192] X. Liu , Y. Jin , C. Yin , et al., “Fabrication of Microplastic‐Free Biomass‐Based Masks: Enhanced Multi‐Functionality With All‐Natural Fibers,” Journal of Hazardous Materials 484 (2025): 136801, 10.1016/j.jhazmat.2024.136801. 39644846

[193] Z. Feng , J. Wen , F. Meng , et al., “In Situ‐Polymerized PANI/WS_2_ Nanocomposites for Highly Sensitive Flexible Ammonia Gas Sensors and Respiration Monitoring Devices,” ACS Applied Nano Materials 7 (2024): 3385–3393.

[194] Z. Wu , Q. Ding , H. Wang , et al., “A Humidity‐Resistant, Sensitive, and Stretchable Hydrogel‐Based Oxygen Sensor for Wireless Health and Environmental Monitoring,” Advanced Functional Materials 34 (2024): 2308280, 10.1002/adfm.202308280.

[195] X. Kang , H. Yu , X. Ma , et al., “Fast Detection of NO_2_ by in_2_O_3_@ZnO Nanofibers Synthesized by Coaxial Electrospinning: Real‐Time Monitoring Application of Smart Mask,” Chemical Engineering Journal 504 (2025): 158872, 10.1016/j.cej.2024.158872.

[196] A. Verma and B. C. Yadav , “Development and Integration of a Hierarchical Pd/WO_3_ Acetone‐Sensing Device for Real‐Time Exhaled Breath Monitoring With Disposable Face Mask,” Journal of Hazardous Materials 463 (2024): 132872, 10.1016/j.jhazmat.2023.132872. 37924704

[197] G. Wu , H. Du , K. Pakravan , et al., “Wearable Room‐Temperature Ethanol Sensor Based on Ti_3_C_2_T* _x_ */Polypyrrole Functionalized Face Mask for Drunk Driving Monitoring,” Carbon 216 (2024): 118565, 10.1016/j.carbon.2023.118565.

[198] H. Liu , C. Fang , J. Zhao , Q. Zhou , Y. Dong , and L. Lin , “The Detection of Acetone in Exhaled Breath Using Gas Pre‐Concentrator by Modified Metal‐Organic Framework Nanoparticles,” Chemical Engineering Journal 498 (2024): 155309, 10.1016/j.cej.2024.155309.

[199] Q. Ma , H. Wang , Y. Sun , J.‐H. Ahn , and B. Wang , “Nanofibrous Metal Oxide Semiconductor for Sensory Face Masks,” Wearable Electronics 1: 189–194, 10.1016/j.wees.2024.09.001.

[200] G. Wu , H. Du , K. Pakravan , et al., “Polyaniline/Ti_3_C_2_T* _x_ * Functionalized Mask Sensors for Monitoring of CO_2_ and human Respiration Rate,” Chemical Engineering Journal 475 (2023): 146228, 10.1016/j.cej.2023.146228.

[201] S. Ma , J. Tang , T. Yan , and Z. Pan , “Performance of Flexible Strain Sensors With Different Transition Mechanisms: A Review,” IEEE Sensors Journal 22 (2022): 7475–7498, 10.1109/JSEN.2022.3156286.

[202] M. A. U. Khalid and S. H. Chang , “Flexible Strain Sensors for Wearable Applications Fabricated Using Novel Functional Nanocomposites: A Review,” Composite Structures 284 (2022): 115214, 10.1016/j.compstruct.2022.115214.

[203] A. del Bosque , X. F. Sánchez‐Romate , D. Patrizi , et al., “Ultrasensitive Flexible Strain Sensors Based on Graphene Nanoplatelets Doped Poly(ethylene glycol) Diglycidyl Ether: Mask Breathing Monitoring for the Internet of Things,” Sensors and Actuators, A: Physical 358 (2023): 114448, 10.1016/j.sna.2023.114448.

[204] B. Xu , F. Ye , R. Chen , et al., “A Supersensitive Wearable Sensor Constructed With PDMS Porous Foam and Multi‐Integrated Conductive Pathways Structure,” Ceramics International 49 (2023): 4641–4649, 10.1016/j.ceramint.2022.09.351.

[205] S. Yang , W. Yang , R. Yin , et al., “Waterproof Conductive Fiber With Microcracked Synergistic Conductive Layer for High‐performance Tunable Wearable Strain Sensor,” Chemical Engineering Journal 453 (2023): 139716, 10.1016/j.cej.2022.139716.

[206] S. Nuthalapati , V. Kedambaimoole , V. Shirhatti , et al., “Flexible Strain Sensor With High Sensitivity, Fast Response, and Good Sensing Range for Wearable Applications,” Nanotechnology 32 (2021): 505506, 10.1088/1361-6528/ac2649. 34517349

[207] K. Xia , X. Chen , X. Shen , et al., “Carbonized Chinese Art Paper‐Based High‐Performance Wearable Strain Sensor for Human Activity Monitoring,” ACS Applied Electronic Materials 1 (2019): 2415–2421.

[208] W. K. Min , C. Won , D. H. Kim , et al., “Strain‐Driven Negative Resistance Switching of Conductive Fibers with Adjustable Sensitivity for Wearable Healthcare Monitoring Systems with Near‐Zero Standby Power,” Advanced Materials 35 (2023): 2303556, 10.1002/adma.202303556. 37177845

[209] S. Yan , M. F. Saleem , H. Ma , et al., “An Ultra‐Sensitive, Rapidly Responsive Strain Sensor Based on Silver Microflakes by Simple Process,” ChemistrySelect 4 (2019): 4407–4415, 10.1002/slct.201900558.

[210] Y. Huang , Y. Zhao , Y. Wang , et al., “Highly Stretchable Strain Sensor Based on Polyurethane Substrate Using Hydrogen Bond‐assisted Laminated Structure for Monitoring of Tiny human Motions,” Smart Materials and Structures 27 (2018): 035013.

[211] C. L. Lim , C. Byrne , and J. K. Lee , “Human Thermoregulation and Measurement of Body Temperature in Exercise and Clinical Settings,” Annals of the Academy of Medicine, Singapore 37 (2008): 347–353, 10.47102/annals-acadmedsg.V37N4p347. 18461221

[212] Q. Li , L. Zhang , X. Tao , and X. Ding , “Review of Flexible Temperature Sensing Networks for Wearable Physiological Monitoring,” Advanced Healthcare Materials 6 (2017): 1601371, 10.1002/adhm.201601371. 28547895

[213] S. Bielska , M. Sibinski , and A. Lukasik , “Polymer Temperature Sensor for Textronic Applications,” Materials Science and Engineering: B 165 (2009): 50–52, 10.1016/j.mseb.2009.07.014.

[214] B. Arman Kuzubasoglu and S. Kursun Bahadir , “Flexible Temperature Sensors: A Review,” Sensors and Actuators, A: Physical 315 (2020): 112282, 10.1016/j.sna.2020.112282.

[215] Y.‐F. Wang , T. Sekine , Y. Takeda , et al., “Fully Printed PEDOT:PSS‐Based Temperature Sensor With High Humidity Stability for Wireless Healthcare Monitoring,” Scientific Reports 10 (2020): 2467, 10.1038/s41598-020-59432-2. 32051489 PMC7016104

[216] R. Liu , L. He , M. Cao , Z. Sun , R. Zhu , and Y. Li , “Flexible Temperature Sensors,” Frontiers in Chemistry 9 (2021), 10.3389/fchem.2021.539678. PMC8492987 34631655

[217] J. Goldsmid , The Physics of Thermoelectric Energy Conversion (Morgan & Claypool Publishers, 2017), 10.1088/978-1-6817-4641-8.

[218] C. Chen , “Evaluation of Resistance–temperature Calibration Equations for NTC Thermistors,” Measurement 42 (2009): 1103–1111, 10.1016/j.measurement.2009.04.004.

[219] Z. Liu , B. Tian , B. Zhang , et al., “A Thin‐Film Temperature Sensor Based on a Flexible Electrode and Substrate,” Microsystems & Nanoengineering 7 (2021): 42, 10.1038/s41378-021-00271-0. 34094587 PMC8166532

[220] T. Zhao , X. Xiao , Y. Wu , et al., “Tracing the Flu Symptom Progression via a Smart Face Mask,” Nano Letters 23 (2023): 8960–8969, 10.1021/acs.nanolett.3c02492. 37750614

[221] S. A. Behera , K. R. Kaja , S. Hajra , et al., “Self‐Powered Wind Flow Monitoring Unit Using Lead‐Free Composites‐Based Triboelectric Nanogenerator,” ACS Applied Energy Materials 8 (2025): 6688–6698.

[222] S. Hajra , S. Panda , K. R. Kaja , M. A. Belal , V. Vivekananthan , and H. J. Kim , “Self‐Powered Fire Safety Indicator Based on Fabric‐Based Triboelectric Nanogenerator,” Energy Technology 13 (2025): 2402488, 10.1002/ente.202402488.

[223] N. Mohamadbeigi , L. Shooshtari , S. Fardindoost , M. Vafaiee , A. Iraji zad , and R. Mohammadpour , “Self‐Powered Triboelectric Nanogenerator Sensor for Detecting Humidity Level and Monitoring Ethanol Variation in a Simulated Exhalation Environment,” Scientific Reports 14 (2024): 1562. 38238422 10.1038/s41598-024-51862-6 PMC10796746

[224] M. Mariello , A. Qualtieri , G. Mele , and M. De Vittorio , “Metal‐Free Multilayer Hybrid PENG Based on Soft Electrospun/‐Sprayed Membranes With Cardanol Additive for Harvesting Energy From Surgical Face Masks,” ACS Applied Materials & Interfaces 13 (2021): 20606–20621, 10.1021/acsami.1c01740. 33896167

[225] S. Pan and Z. Zhang , “Fundamental Theories and Basic Principles of Triboelectric Effect: A Review,” Friction 7 (2019): 2–17, 10.1007/s40544-018-0217-7.

[226] H. Zou , L. Guo , H. Xue , et al., “Quantifying and Understanding the Triboelectric Series of Inorganic Non‐Metallic Materials,” Nature Communications 11 (2020): 2093, 10.1038/s41467-020-15926-1. PMC7190865 32350259

[227] V. Kumar , P. Kumar , R. Deka , Z. Abbas , and S. M. Mobin , “Recent Development of Morphology‐Controlled Hybrid Nanomaterials for Triboelectric Nanogenerator: A Review,” Chemical Record 22 (2022): 202200067, 10.1002/tcr.202200067. 35686889

[228] J. Huang , Y. Huang , T. Li , H. Xu , H. Wu , and Z. Su , “Respiration‐Driven Ammonia Sensing Mask for Multifunctional Self‐Powered Monitoring Application,” Chemical Engineering Journal 507 (2025): 160598, 10.1016/j.cej.2025.160598.

[229] S. Hu , B. Zhang , T. Han , et al., “Triboelectrically Self‐Sensing respiratory Ventilator Masks for Monitoring, Diagnosis, Therapy, and Human–Machine Interaction,” Nano Energy 124 (2024): 109516, 10.1016/j.nanoen.2024.109516.

[230] Q. Lu , H. Chen , Y. Zeng , et al., “Intelligent Facemask Based on Triboelectric Nanogenerator for Respiratory Monitoring,” Nano Energy 91 (2022): 106612, 10.1016/j.nanoen.2021.106612. 34660183 PMC8505024

[231] S. Wang , H. Tai , B. Liu , et al., “A Facile Respiration‐Driven Triboelectric Nanogenerator for Multifunctional Respiratory Monitoring,” Nano Energy 58 (2019): 312–321, 10.1016/j.nanoen.2019.01.042.

[232] M. Karimi Kisomi , S. Seddighi , R. Mohammadpour , and A. Rezaniakolaei , “Enhancing Air Filtration Efficiency With Triboelectric Nanogenerators in Face Masks and Industrial Filters,” Nano Energy 112 (2023): 108514, 10.1016/j.nanoen.2023.108514.

[233] J. Yun , J. Park , S. Jeong , D. Hong , and D. Kim , “A Mask‐Shaped Respiration Sensor Using Triboelectricity and a Machine Learning Approach Toward Smart Sleep Monitoring Systems,” Polymers 14 (2022): 3549, 10.3390/polym14173549. 36080623 PMC9460850

[234] H. He , J. Guo , B. Illés , et al., “Monitoring Multi‐Respiratory Indices via a Smart Nanofibrous Mask Filter Based on a Triboelectric Nanogenerator,” Nano Energy 89 (2021): 106418, 10.1016/j.nanoen.2021.106418.

[235] Y. Su , G. Chen , C. Chen , et al., “Self‐Powered Respiration Monitoring Enabled by a Triboelectric Nanogenerator,” Advanced Materials 33 (2021): 2101262, 10.1002/adma.202101262. 34240473

[236] W. Kwak , J. Yin , S. Wang , and J. Chen , “Advances in Triboelectric Nanogenerators for Self‐Powered Wearable Respiratory Monitoring,” FlexMat 1 (2024): 5–22, 10.1002/flm2.10.

[237] W. Liu , Y. Sun , A. Cui , et al., “Electrothermal Sterilization and Self‐Powered Real‐Time Respiratory Monitoring of Reusable Mask Based on Ag Micro‐Mesh Films,” Nano Energy 105 (2023): 107987, 10.1016/j.nanoen.2022.107987. 36373076 PMC9636608

[238] Y. Liu , J. Mo , Q. Fu , et al., “Enhancement of Triboelectric Charge Density by Chemical Functionalization,” Advanced Functional Materials 30 (2020): 2004714, 10.1002/adfm.202004714.

